# Increasing biodiversity knowledge through social media: A case study from tropical Bangladesh

**DOI:** 10.1093/biosci/biad042

**Published:** 2023-06-08

**Authors:** Shawan Chowdhury, Upama Aich, Md Rokonuzzaman, Shofiul Alam, Priyanka Das, Asma Siddika, Sultan Ahmed, Mahzabin Muzahid Labi, Moreno Di Marco, Richard A Fuller, Corey T Callaghan

**Affiliations:** School of Biological Sciences, University of Queensland, in Saint Lucia, Queensland, Australia; Institute of Biodiversity, Friedrich Schiller University Jena, in Jena, Germany; Helmholtz Centre for Environmental Research—UFZ, Department of Ecosystem Services, in Leipzig, Germany; German Centre for Integrative Biodiversity Research, in Leipzig, Germany; School of Biological Sciences, Monash University, in Clayton, Victoria, Australia; Department of Zoology, University of Dhaka, in Dhaka, Bangladesh; Department of Zoology, University of Dhaka, in Dhaka, Bangladesh; Department of Zoology, University of Dhaka, in Dhaka, Bangladesh; Department of Zoology, University of Dhaka, in Dhaka, Bangladesh; Department of Zoology, University of Dhaka, in Dhaka, Bangladesh; Department of Zoology, University of Dhaka, in Dhaka, Bangladesh; Department of Biology and Biotechnologies, Sapienza University of Rome, in Rome, Italy; School of Biological Sciences, University of Queensland, in Saint Lucia, Queensland, Australia; Department of Wildlife Ecology and Conservation, Fort Lauderdale, Florida, United States; Research and Education Center, University of Florida, Davie, Florida, United States

**Keywords:** citizen science, biodiversity conservation, Facebook, social media data, Wallacean shortfall

## Abstract

Citizen science programs are becoming increasingly popular among naturalists but remain heavily biased taxonomically and geographically. However, with the explosive popularity of social media and the near-ubiquitous availability of smartphones, many post wildlife photographs on social media. Here, we illustrate the potential of harvesting these data to enhance our biodiversity understanding using Bangladesh, a tropical biodiverse country, as a case study. We compared biodiversity records extracted from Facebook with those from the Global Biodiversity Information Facility (GBIF), collating geospatial records for 1013 unique species, including 970 species from Facebook and 712 species from GBIF. Although most observation records were biased toward major cities, the Facebook records were more evenly spatially distributed. About 86% of the Threatened species records were from Facebook, whereas the GBIF records were almost entirely Of Least Concern species. To reduce the global biodiversity data shortfall, a key research priority now is the development of mechanisms for extracting and interpreting social media biodiversity data.

Human pressure on the environment, including climate change, agricultural expansion, overexploitation of natural resources, and habitat loss are causing many species to decline globally (Butchart et al. 2010, Maxwell et al. 2016, Isbell et al. 2022, Chowdhury et al. 2022a, Chowdhury 2023). However, monitoring of biodiversity and its trends is heavily biased toward the developed world (Sala et al. 2000, Fraixedas et al. 2022). Assessing temporal biodiversity trends requires long-term time-series data (Dornelas et al. 2013), which are unavailable from most of the world (Collen et al. 2008, Hughes et al. 2021). The Kunming–Montreal Biodiversity Framework of the Convention on Biological Diversity prioritizes area-based conservation, aiming to halt global biodiversity threats, and to achieve 30% protected area coverage by 2030 (CBD 2022). Achieving such ambitious targets requires detailed knowledge of biodiversity distribution, especially to identify where to designate protected areas and to inform conservation decisions and monitoring (Maxwell et al. 2020). Most of the biodiversity hotspots are distributed in tropical forests; although these areas occupy less than 2% of the Earth's land surface, they harbor more than 50% of the global biodiversity (Myers et al. 2000, Collen et al. 2008). Investing special attention toward tropical regions is essential to effectively arrest biodiversity loss (Mittermeier et al. 2011), but our current understanding of the status and trends of tropical biodiversity remains scarce (Collen et al. 2008, Chowdhury et al. 2021a).

Although systematic recording of biodiversity data has been widely practiced in the developed world for centuries, our understanding of species distribution remains vastly limited from many developing countries (Schmeller et al. 2017). Citizen science (also known as *community science*) is helping to address this global biodiversity data gap, and the concept is rapidly advancing globally (Bonney et al. 2014, Prudic et al. 2017, Flockhart et al. 2019, Yue et al. 2019, Jarić et al. 2020, Callaghan et al. 2021). Citizen science initiatives such as eBird and iNaturalist are becoming popular among both amateur and professional naturalists (Pocock et al. 2018, Callaghan et al. 2021); however, most users are from Europe and North America (Amano et al. 2016). As a result, the available data of the largest global biodiversity repository—the Global Biodiversity Information Facility (GBIF)—is highly biased toward the global north (Chandler et al. 2017, Troudet et al. 2017).

Although GBIF is the largest biodiversity aggregator, there still exists much data that do not make it into GBIF. With increasing popularity of social media and the wider availability of digital cameras, many people post biodiversity photographs on social media channels such as Facebook, Twitter, or Flickr (Barve 2014, Toivonen et al. 2019, Marcenò et al. 2021), representing a source of biodiversity data not customarily indexed in GBIF. Previous work has demonstrated the wealth of photographs on social media of global biodiversity, including many Threatened species (Hausmann et al. 2019, Steven et al. 2019, Mohd Rameli et al. 2020, Coram et al. 2021, Sbragaglia et al. 2021, Lin et al. 2022). Among a broad array of social media providers, Facebook has become the largest network in the world (Anderson et al. 2012). Facebook contains thousands of biodiversity groups globally, each specialized on different taxa. In these Facebook groups, the group moderators or administrators help to correctly identify species photographed in the field, including many records of narrow-ranged endemics or new country occurrences (Marcenò et al. 2021, Chowdhury et al. 2021b, Bergman et al. 2022). However, Facebook data have rarely been used in national or international biodiversity assessments (Chowdhury et al. 2021b). This is mainly because of the difficulties in mobilizing such data.

In the present article, we illustrate the potential of mobilizing biodiversity data from Facebook, using Bangladesh—a highly biodiverse South Asian developing country—as a case study. Bangladesh is home to several hundred species of vertebrates and invertebrates, of which 60 species are globally Threatened (IUCN Bangladesh 2015, Mukul et al. 2018). We collated data from both Facebook and GBIF for Bangladeshi IUCN Red List-listed species (1013 of 1619 species; 606 species were not listed in either database) and tested the potential value of mobilizing (i.e., extracting and getting into a useful format) such biodiversity data from Facebook for biodiversity conservation assessments. Specifically, we report how social media archives can provide biodiversity data that complements and often surpasses those available from formal databases, discuss the challenges in obtaining biodiversity data from social media, including possible biases in their interpretation, and provide potential solutions on how future work in conservation science and practice could benefit from incorporating them.

## Spatial occurrence records

We collected a complete checklist of animal diversity in Bangladesh from the most recent national Red List data book (1619 species; IUCN Bangladesh 2015). We followed a range of approaches to collate spatial data. First, we downloaded spatial occurrence records from the GBIF (www.gbif.org; GBIF 2022) using the rgbif package (Chamberlain et al. 2022) in R (R version 4.0.4; R Core Team 2020). GBIF is a global data infrastructure network that compiles species occurrence records from a range of sources, including museum specimens and citizen science projects (Heberling et al. 2021), so to avoid repetition, we did not collect data from other biodiversity repositories (Chowdhury et al. 2021b, 2021c). Furthermore, we searched for species distribution records in seven Facebook groups (Amphibians and Reptiles of Bangladesh 2021, Biodiversity of Bangladesh 2021, Biodiversity of Greater Kushtia 2021, Birds Bangladesh 2021, Butterfly Bangladesh 2021, Deep Ecology And Snake Rescue Foundation 2021, Mammals of Bangladesh 2021), following the method described by Chowdhury and colleagues (2021b). These are the seven most popular Facebook groups for biodiversity in Bangladesh; therefore, we did not search other Facebook groups. Besides, each of these Facebook group is regularly moderated by regional international taxonomic experts who verify the taxonomic information for each photograph. In each group, we searched by the species’s English common name, obtained from IUCN Bangladesh (2015) and double-checked the identification in each photograph. When sharing photographs in these groups, photographers need to specify location information, which the moderators regularly cross-check. We extracted the location information from each post and georeferenced the observations using Google Maps (using the locality information from the photographs; https://maps.google.com). On average, it took approximately 33 minutes to harvest the data for each species. We excluded photographs if the identification was incomplete (not up to species level) or wrong if the photograph did not allow clear taxonomic identification or if the location was unspecified or could not be accurately determined (Chowdhury et al. 2021b, 2021c).

## Data cleaning

We performed a cleaning process for GBIF data using the CoordinateCleaner package (Zizka et al. 2019) in R. We removed duplicate records, those with precision uncertainty over 10 kilometers, those with imprecise coordinates (zero coordinates, integers, records in oceans), and those with invalid coordinates (where the specified locality was incompatible with the coordinates given; Chowdhury et al. 2021b, 2021d).

To control for sampling bias, we followed the spatial thinning approach from the spThin R package (Aiello-Lammens et al. 2015), and we only considered a single occurrence record at 1 square Kilometer (km^2^) resolution for each species. Our final database contained 182,383 unique geospatial records.

## Estimating the extent of occurrence

We estimated the extent of occurrence (EOO) of each species as the minimum convex polygon around their occurrence records (Joppa et al. 2016), using the rgeos package in R (Bivand and Rundel 2020). We measure EOO using two data sets for each species: records only from Facebook and records only from GBIF. After creating the range polygons, we converted these polygons into raster format for computational efficiency. After creating the occurrence maps for each species, we rasterized each map at 1-km^2^ resolution and reprojected the map into the World Behrmann coordinate system (ESRI: 54,017). Given that Bangladesh is surrounded by several countries and open oceans, we masked each species range polygon by the terrestrial map of Bangladesh, to define country-level estimates of species EOO (i.e., as would be used for national extinction risk assessments).

## Distribution of spatial data

**Figure 1.**
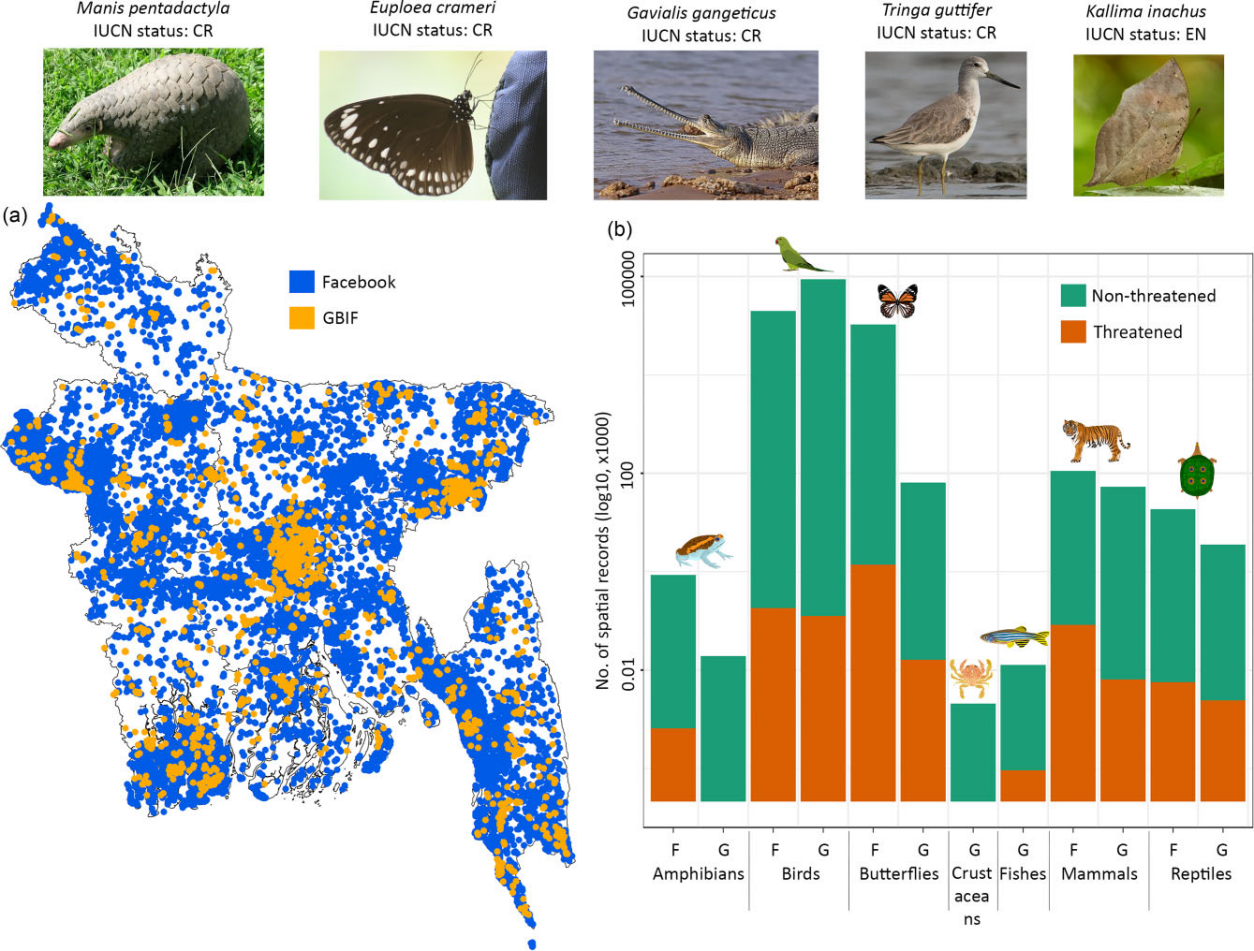
Distribution (a) and taxonomic group-wise number (b) of geospatial records for Bangladeshi animals using records from both Facebook (F) and GBIF (G). The photographs are some species for which we only obtained distribution records from Facebook. The source of these photographs are the following: Ms Sarita Jnawali (*Manis pentadactyla*), Gerard Chartier (*Euploea crameri*), Charles J Sharp (*Gavialis gangeticus*), Jason Thompson (*Tringa guttifer*), and Shawan Chowdhury (*Kallima inachus*).

Overall, our combined data set included 182,383 geospatial records for 1013 species. Nearly 25% (44,740) of the records were from Facebook, and 75% (137,643) were from GBIF. The combined spatial data were heavily biased toward major cities. About 53% of the geospatial records were from the central part of the country (figure 1, supplemental figure S1).

We obtained occurrence data for 970 and 712 species from Facebook and GBIF, respectively. There were 346 species unique to only one of the data sets: 302 species were found only on Facebook, whereas 44 species were found only on GBIF. There was marked variation across taxonomic groups and Threatened status combinations (figure 1). Although 30% (25 of 83 species) of the unique bird species were from GBIF, there was no unique amphibian species in GBIF, which means that all the amphibian species recorded in GBIF were also found on Facebook. Of the other taxa, 143 of the 145 species of butterflies, 38 of 39 species of reptiles, and 35 of 37 species of mammals were from Facebook. Moreover, the percentages of nonthreatened unique species were higher in all groups, except for butterflies, where 77% of unique species (112 species) were Threatened, of which 110 species were from Facebook.

We observed striking differences among taxa, both in the spatial distribution of records and the number of species records in each data set. For example, Facebook yielded only 20% of all bird records but 93% of all butterfly records. In contrast, we did not obtain any records of crustaceans or fishes from Facebook (supplemental table S1). Facebook almost always surpassed GBIF in terms of the number of species recorded of a given taxon, with the only exception being crustaceans and fishes. Although we obtained only a slightly higher number of bird species from Facebook (497 species versus 464 species from GBIF), there were remarkable differences for amphibians (45 species from Facebook, 17 species from GBIF) and butterflies (265 species from Facebook, 124 species from GBIF).

We obtained substantially more records of Threatened species than nonthreatened species from Facebook. Even though GBIF contained three times more spatial distribution records than Facebook, 86% of the available distribution records on Threatened species were from Facebook. In contrast, 99% of GBIF records were Of Least Concern species.

## Differences in species range size

**Figure 2.**
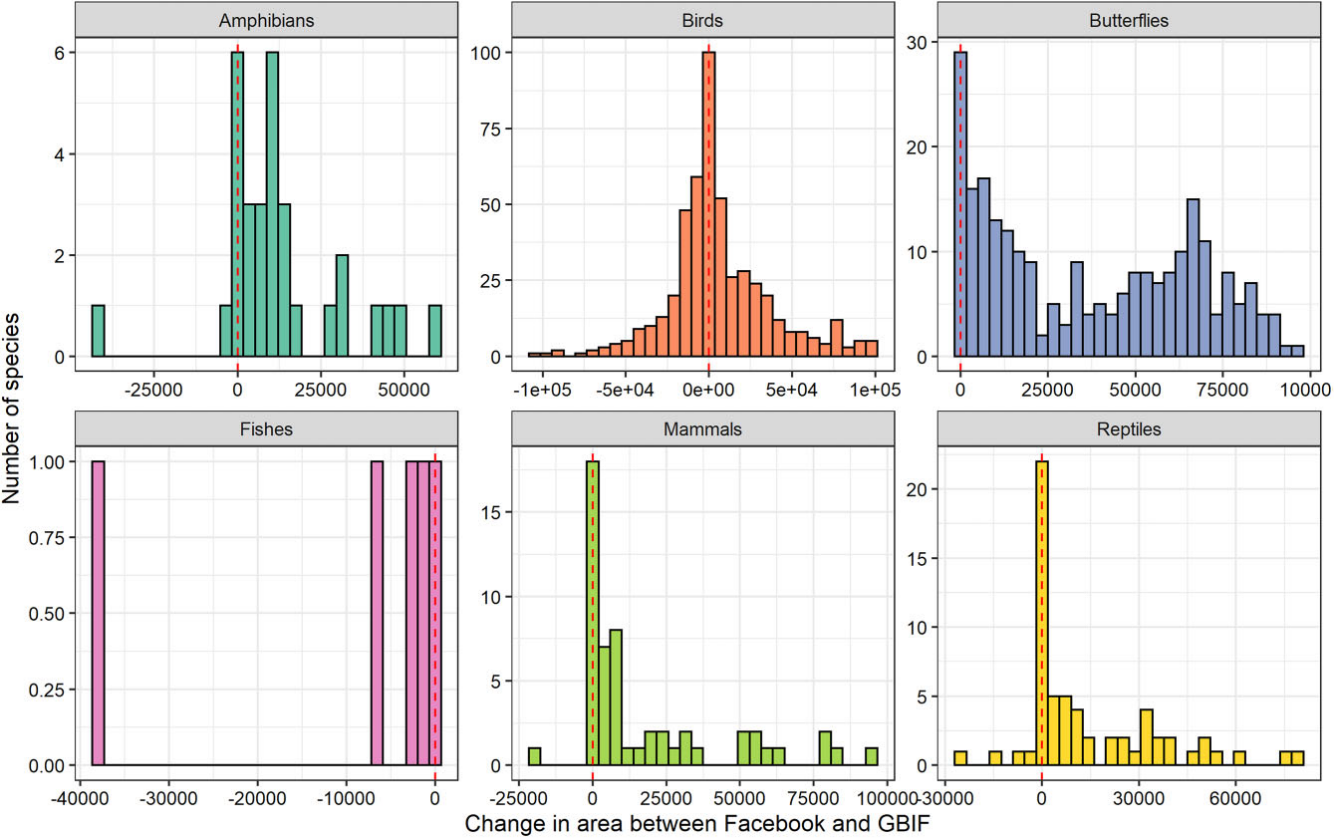
The difference in extent of occurrence (EOO, in square kilometers) for Bangladeshi animals using geospatial records from Facebook and GBIF. The dashed line indicates where the EOO estimates from Facebook and GBIF were identical.

We evaluated the effect of measuring species distribution size using minimum convex polygons around occurrences (EOO; Joppa et al. 2016) for each species, using spatial distribution records from Facebook and GBIF. We obtained the EOO for 865 species, including 222 Threatened and 643 nonthreatened species, and found that the mean EOO size was 25,409 km^2^ using GBIF and 41,877 km^2^ using Facebook. The EOO obtained from Facebook data was larger than the EOO from GBIF for more than 72% of species, but the difference in EOO varied markedly across species (figure 2). For example, the EOO of *Spilopelia chinensis* (spotted dove) was 100,000 km^2^ larger in GBIF than in Facebook, whereas for *Pernis ptilorhyncus* (crested honey buzzard), it was 100,000 km^2^ smaller (figure 2).

The difference in EOO measured between data sets was typically higher for Threatened species. We obtained larger EOO using Facebook records for about 95% of the Threatened species (*n* = 210). Although the EOO was always larger using Facebook records (except for fishes), the percentages varied across taxa. The EOO for butterflies was always larger when using Facebook data, whereas for birds only 55% of species had a larger EOO from Facebook (figure 2).

## The importance of Facebook records in reducing the Wallacean gap

We showed that photographs harvested from Facebook can play a vital role in reducing the biodiversity knowledge gap in developing tropical countries rich in biodiversity, such as Bangladesh. The yearly growth in the number of species and occurrence records indicates that, even though records from GBIF started much earlier (GBIF started in 2001, but it contains museum records from a few centuries ago, Heberling et al. 2021; Facebook only launched in 2004, Anderson et al. 2012). Both sources of records increased exponentially after 2010 and Facebook data are becoming proportionally more numerous. In addition to containing thousands of records, Facebook can provide data for many otherwise undersampled Threatened species and areas. For 30% of species, many of which are nationally listed as Threatened, we obtained records only from Facebook. For example, without considering Facebook data, we would not have any distribution information on several Critically Endangered species (e.g., *Manis pentadactyla, Euploea crameri, Gavialis gangeticus, Tringa guttifer*), given their distribution (in Bangladesh) is unknown in the global repository (GBIF).

We obtained striking differences between the EOO estimated with the two data sets because, for most species, the Facebook records were much more widespread than the GBIF records were (figure 1, figure S1). This result was confirmed for all well-sampled groups but birds and demonstrate the risk of underestimating EOO and potentially overestimating the extinction risk when occurrence data disregard large parts of the species distribution. In fact, we found an average EOO size of 25,000 km^2^ using GBIF data, which is close to the threshold than can trigger the category of Vulnerable under IUCN Red List criterion B1 (20,000 km^2^, if concurring threat conditions are met). Furthermore, the proportion of Threatened species records was higher in Facebook than in GBIF, emphasizing the feasibility and importance of extracting biodiversity data from social media sites. A combination of citizen science data with global data sources, which are freely accessible and available online, can help large scale quantitative ecosystem assessments, including global and continental level ecosystem accounting and ecosystem modelling (Willemen et al. 2015). We hypothesize that the importance of social media repositories such as Facebook, as is shown in the present article, are important in other developing countries where citizen science is less mobilized (especially in Asia). Instead, in developed countries where iNaturalist and other platforms are heavily used, such as Australia (Mesaglio and Callaghan 2021), the potential value of adding Facebook records may be reduced.

## Difficulties in obtaining Facebook records and the way forward

Although Facebook offers the potential to increase the availability of biodiversity data, acquiring such data can be challenging (Lin et al. 2015, Chamberlain 2018, Chowdhury et al. 2021b, Giovos et al. 2018, Liberatore et al. 2018). The biodiversity data collected from Facebook can often result in a reduced sample size because of the multistep process associated with data collection from social media, lowering the quality and quantity of data (Liberatore et al. 2018, Cloutier et al. 2021, Marcenò et al. 2021). For example, we had to manually georeference each biodiversity record obtained from Facebook, which was time consuming and not exact because of not having precise geolocation information. Because citizen science data do not typically follow a particular collection strategy, information tends to be biased toward urban and semiurban areas because of higher population density, higher tech use, education, and awareness (Di Minin et al. 2015). For instance, areas near Dhaka, Chittagong, and Sylhet division are largely oversampled compared with the northern and southwestern regions of Bangladesh. Besides, less charismatic and common species (e.g., small insects) or species that require substantial effort to document (e.g., nocturnal or aquatic animals) are scarcely represented in either GBIF or Facebook (Di Marco et al. 2017, Giovos et al. 2018, Hausmann et al. 2019, Marcenò et al. 2021). Promoting standardized international hashtags for nongovernment organizations, institutions, and citizen scientists could allow researchers to quickly access and consolidate data (Abreo et al. 2019, Kelly et al. 2020, von Gönner et al. 2022).

Because of not having adequate taxonomic details, we removed some distribution records, given that identifying closely related or morphologically cryptic species can be challenging because of a lack of expertise (Aravind 2013, Abreo et al. 2019, Coram et al. 2021). However, social media data can be used to teach and learn more about species identification, spatiotemporal patterns of species occurrence, values, and activities related to biodiversity conservation of different groups of people (Di Minin et al. 2015, Walden-Schreiner et al. 2018). In addition to the potential to increase biodiversity data, social media can also be leveraged to spread awareness and raise conservation actions, often reaching a wider audience than citizen science (Bergman et al. 2022). Artificial intelligence (AI) can substantially affect data scraping (Di Minin et al. 2018, Christin et al. 2019, Lamba et al. 2019, Jarić et al. 2020, Høye et al. 2021). Although most AI systems (e.g., iNaturalist's Computer Vision) can only detect or recognize already seen (or learned) objects or concepts, benchmark data sets of images can be organized to precisely assess the limits of AI systems’ ability and reduce these, highlighting areas where human expertise is still required (August et al. 2020, Lawu et al. 2021). Deep learning models can be developed with training data sets that capture discriminant visual patterns (August et al. 2020, Kirkhope et al. 2010, Marcenò et al. 2021). AI could also be used to help sort and georeference the data to save time. This can result in better identification of species, documentation of critical information, and increased data reliability.

## Conservation implications

Effective area-based conservation planning, as it was envisaged in the Kunming–Montreal Biodiversity Framework of the UN Convention on Biological Diversity, requires the identification of important biodiversity areas (Maxwell et al. 2020, Allan et al. 2022, Jetz et al. 2022, Chowdhury et al. 2023). Having adequate knowledge of species distribution is essential to identify such areas, but this information is often missing in many parts of the world including biodiversity rich tropical countries (Collen et al. 2008, Meyer et al. 2015, Troudet et al. 2017). We showed that extracting biodiversity data from social media can reduce this global gap in biodiversity knowledge. Even though extracting data from social media is not currently a straightforward process and can be time consuming, the results can often be worth the effort. Although the correlations between Facebook and other social media data sources can vary substantially, comparing and compiling a broader range of data could provide a holistic view of citizen science data sources (Wilkins et al. 2021). Collating data from multiple repositories will help inform efficient conservation planning at the local and international levels and assess protected area performance (Amano et al. 2016, Tulloch et al. 2018, Chowdhury et al. 2021b, 2023).

## Supplementary Material

biad042_Supplemental_File

## Author Biographical

Shawan Chowdhury (s.chowdhury@uqconnect.edu.au;) and Richard A. Fuller are affiliated with the School of Biological Sciences at the University of Queensland, in Saint Lucia, Queensland, Australia. Shawan Chowdhury is also affiliated with the Institute of Biodiversity at Friedrich Schiller University Jena, in Jena, Germany; with the Helmholtz Centre for Environmental Research—UFZ, Department of Ecosystem Services, in Leipzig, Germany; and with the German Centre for Integrative Biodiversity Research, in Leipzig, Germany. Upama Aich is affiliated with the School of Biological Sciences at Monash University, in Clayton, Victoria, Australia. Md Rokonuzzaman, Shofiul Alam, Priyanka Das, Asma Siddika, Sultan Ahmed, and Mahzabin Muzahid Labi are affiliated with the Department of Zoology, at the University of Dhaka, in Dhaka, Bangladesh. Moreno Di Marco is affiliated with the Department of Biology and Biotechnologies at Sapienza University of Rome, in Rome, Italy. Corey T. Callaghan is affiliated with the Department of Wildlife Ecology and Conservation at the Fort Lauderdale Research and Education Center, at the University of Florida, in Davie, Florida, in the United States.
