# Supplementary material for: Increasing biodiversity knowledge through social media: A case study from tropical Bangladesh
Source: Bioscience. 2023 Jun 8;73(6):453–9. doi: 10.1093/biosci/biad042 (PMC10308356; doi:10.1093/biosci/biad042)

**Table S1.** Species-wise number of records obtained from Facebook and GBIF

| group | species | source | iucn | n_rec |
| --- | --- | --- | --- | --- |
| Amphibians | Amolops_marmoratus | Facebook | VU | 2 |
| Amphibians | Amolops_marmoratus | Overall | VU | 2 |
| Amphibians | Chikila_fulleri | Facebook | CR | 2 |
| Amphibians | Chikila_fulleri | Overall | CR | 2 |
| Amphibians | Chiromantis_doriae | Facebook | EN | 1 |
| Amphibians | Chiromantis_doriae | Overall | EN | 1 |
| Amphibians | Chiromantis_simus | Facebook | LC | 5 |
| Amphibians | Chiromantis_simus | Overall | LC | 5 |
| Amphibians | Chiromantis_vittatus | Facebook | LC | 10 |
| Amphibians | Chiromantis_vittatus | Overall | LC | 10 |
| Amphibians | Clinotarsus_alticola | Facebook | LC | 7 |
| Amphibians | Clinotarsus_alticola | GBIF | LC | 2 |
| Amphibians | Clinotarsus_alticola | Overall | LC | 9 |
| Amphibians | Duttaphrynus_melanostictus | Facebook | LC | 14 |
| Amphibians | Duttaphrynus_melanostictus | GBIF | LC | 40 |
| Amphibians | Duttaphrynus_melanostictus | Overall | LC | 54 |
| Amphibians | Duttaphrynus_stomaticus | Facebook | LC | 1 |
| Amphibians | Duttaphrynus_stomaticus | GBIF | LC | 2 |
| Amphibians | Duttaphrynus_stomaticus | Overall | LC | 3 |
| Amphibians | Euphlyctis_cyanophlyctis | Facebook | LC | 10 |
| Amphibians | Euphlyctis_cyanophlyctis | GBIF | LC | 40 |
| Amphibians | Euphlyctis_cyanophlyctis | Overall | LC | 50 |
| Amphibians | Euphlyctis_hexadactylus | Facebook | LC | 1 |
| Amphibians | Euphlyctis_hexadactylus | GBIF | LC | 18 |
| Amphibians | Euphlyctis_hexadactylus | Overall | LC | 19 |
| Amphibians | Fejervarya_asmati | Facebook | LC | 6 |
| Amphibians | Fejervarya_asmati | Overall | LC | 6 |
| Amphibians | Fejervarya_cancrivora | Facebook | LC | 1 |
| Amphibians | Fejervarya_cancrivora | Overall | LC | 1 |
| Amphibians | Fejervarya_nepalensis | Facebook | LC | 7 |
| Amphibians | Fejervarya_nepalensis | Overall | LC | 7 |
| Amphibians | Fejervarya_pierrei | Facebook | LC | 9 |
| Amphibians | Fejervarya_pierrei | Overall | LC | 9 |
| Amphibians | Fejervarya_syhadrensis | Facebook | LC | 6 |
| Amphibians | Fejervarya_syhadrensis | Overall | LC | 6 |
| Amphibians | Fejervarya_teraiensis | Facebook | LC | 8 |
| Amphibians | Fejervarya_teraiensis | Overall | LC | 8 |
| Amphibians | Hoplobatrachus_crassus | Facebook | NT | 2 |
| Amphibians | Hoplobatrachus_crassus | GBIF | NT | 1 |
| Amphibians | Hoplobatrachus_crassus | Overall | NT | 3 |
| Amphibians | Hoplobatrachus_litoralis | Facebook | NT | 3 |
| Amphibians | Hoplobatrachus_litoralis | Overall | NT | 3 |
| Amphibians | Hoplobatrachus_tigerinus | Facebook | LC | 10 |
| Amphibians | Hoplobatrachus_tigerinus | GBIF | LC | 26 |
| Amphibians | Hoplobatrachus_tigerinus | Overall | LC | 36 |
| Amphibians | Humerana_humeralis | Facebook | LC | 6 |
| Amphibians | Humerana_humeralis | GBIF | LC | 1 |
| Amphibians | Humerana_humeralis | Overall | LC | 7 |
| Amphibians | Hylarana_leptoglossa | Facebook | LC | 9 |
| Amphibians | Hylarana_leptoglossa | Overall | LC | 9 |
| Amphibians | Hylarana_nicobariensis | Facebook | DD | 1 |
| Amphibians | Hylarana_nicobariensis | Overall | DD | 1 |
| Amphibians | Hylarana_taipehensis | Facebook | DD | 7 |
| Amphibians | Hylarana_taipehensis | GBIF | DD | 4 |
| Amphibians | Hylarana_taipehensis | Overall | DD | 11 |
| Amphibians | Hylarana_tytleri | Facebook | LC | 12 |
| Amphibians | Hylarana_tytleri | GBIF | LC | 4 |
| Amphibians | Hylarana_tytleri | Overall | LC | 16 |
| Amphibians | Ichthyophis_garoensis | Facebook | DD | 1 |
| Amphibians | Ichthyophis_garoensis | Overall | DD | 1 |
| Amphibians | Kalophrynus_interlineatus | Facebook | DD | 1 |
| Amphibians | Kalophrynus_interlineatus | Overall | DD | 1 |
| Amphibians | Kaloula_pulchra | Facebook | NT | 10 |
| Amphibians | Kaloula_pulchra | GBIF | NT | 4 |
| Amphibians | Kaloula_pulchra | Overall | NT | 14 |
| Amphibians | Kaloula_taprobanica | Facebook | VU | 1 |
| Amphibians | Kaloula_taprobanica | GBIF | VU | 1 |
| Amphibians | Kaloula_taprobanica | Overall | VU | 2 |
| Amphibians | Leptobrachium_smithi | Facebook | LC | 6 |
| Amphibians | Leptobrachium_smithi | Overall | LC | 6 |
| Amphibians | Limnonectes_laticeps | Facebook | VU | 1 |
| Amphibians | Limnonectes_laticeps | Overall | VU | 1 |
| Amphibians | Microhyla_berdmorei | Facebook | LC | 12 |
| Amphibians | Microhyla_berdmorei | GBIF | LC | 2 |
| Amphibians | Microhyla_berdmorei | Overall | LC | 14 |
| Amphibians | Microhyla_mukhlesuri | Facebook | DD | 1 |
| Amphibians | Microhyla_mukhlesuri | Overall | DD | 1 |
| Amphibians | Microhyla_mymensinghensis | Facebook | LC | 5 |
| Amphibians | Microhyla_mymensinghensis | GBIF | LC | 1 |
| Amphibians | Microhyla_mymensinghensis | Overall | LC | 6 |
| Amphibians | Microhyla_ornata | Facebook | LC | 8 |
| Amphibians | Microhyla_ornata | GBIF | LC | 11 |
| Amphibians | Microhyla_ornata | Overall | LC | 19 |
| Amphibians | Microhyla_rubra | Facebook | LC | 6 |
| Amphibians | Microhyla_rubra | Overall | LC | 6 |
| Amphibians | Occidozyga_lima | Facebook | LC | 3 |
| Amphibians | Occidozyga_lima | Overall | LC | 3 |
| Amphibians | Odorrana_chloronota | Facebook | DD | 1 |
| Amphibians | Odorrana_chloronota | Overall | DD | 1 |
| Amphibians | Polypedates_leucomystax | Facebook | LC | 14 |
| Amphibians | Polypedates_leucomystax | GBIF | LC | 4 |
| Amphibians | Polypedates_leucomystax | Overall | LC | 18 |
| Amphibians | Polypedates_maculatus | Facebook | LC | 3 |
| Amphibians | Polypedates_maculatus | Overall | LC | 3 |
| Amphibians | Pterorana_khare | Facebook | CR | 1 |
| Amphibians | Pterorana_khare | Overall | CR | 1 |
| Amphibians | Raorchestes_parvulus | Facebook | NT | 1 |
| Amphibians | Raorchestes_parvulus | Overall | NT | 1 |
| Amphibians | Rhacophorus_bipunctatus | Facebook | LC | 7 |
| Amphibians | Rhacophorus_bipunctatus | GBIF | LC | 4 |
| Amphibians | Rhacophorus_bipunctatus | Overall | LC | 11 |
| Amphibians | Theloderma_asperum | Facebook | EN | 2 |
| Amphibians | Theloderma_asperum | Overall | EN | 2 |
| Amphibians | Uperodon_globulosus | Facebook | VU | 3 |
| Amphibians | Uperodon_globulosus | Overall | VU | 3 |
| Amphibians | Xenophrys_parva | Facebook | NT | 4 |
| Amphibians | Xenophrys_parva | Overall | NT | 4 |
| Birds | Abroscopus_superciliaris | Facebook | NT | 7 |
| Birds | Abroscopus_superciliaris | GBIF | NT | 3 |
| Birds | Abroscopus_superciliaris | Overall | NT | 10 |
| Birds | Accipiter_badius | Facebook | LC | 95 |
| Birds | Accipiter_badius | GBIF | LC | 405 |
| Birds | Accipiter_badius | Overall | LC | 500 |
| Birds | Accipiter_gentilis | GBIF | DD | 4 |
| Birds | Accipiter_gentilis | Overall | DD | 4 |
| Birds | Accipiter_nisus | Facebook | LC | 9 |
| Birds | Accipiter_nisus | GBIF | LC | 7 |
| Birds | Accipiter_nisus | Overall | LC | 16 |
| Birds | Accipiter_trivirgatus | Facebook | LC | 65 |
| Birds | Accipiter_trivirgatus | GBIF | LC | 52 |
| Birds | Accipiter_trivirgatus | Overall | LC | 117 |
| Birds | Accipiter_virgatus | Facebook | LC | 10 |
| Birds | Accipiter_virgatus | Overall | LC | 10 |
| Birds | Acridotheres_fuscus | Facebook | LC | 175 |
| Birds | Acridotheres_fuscus | GBIF | LC | 3596 |
| Birds | Acridotheres_fuscus | Overall | LC | 3771 |
| Birds | Acridotheres_ginginianus | Facebook | LC | 94 |
| Birds | Acridotheres_ginginianus | GBIF | LC | 416 |
| Birds | Acridotheres_ginginianus | Overall | LC | 510 |
| Birds | Acridotheres_grandis | GBIF | LC | 8 |
| Birds | Acridotheres_grandis | Overall | LC | 8 |
| Birds | Acridotheres_tristis | Facebook | LC | 267 |
| Birds | Acridotheres_tristis | GBIF | LC | 4241 |
| Birds | Acridotheres_tristis | Overall | LC | 4508 |
| Birds | Acrocephalus_aedon | Facebook | LC | 54 |
| Birds | Acrocephalus_aedon | GBIF | LC | 3 |
| Birds | Acrocephalus_aedon | Overall | LC | 57 |
| Birds | Acrocephalus_agricola | Facebook | LC | 35 |
| Birds | Acrocephalus_agricola | GBIF | LC | 98 |
| Birds | Acrocephalus_agricola | Overall | LC | 133 |
| Birds | Acrocephalus_bistrigiceps | Facebook | LC | 15 |
| Birds | Acrocephalus_bistrigiceps | GBIF | LC | 12 |
| Birds | Acrocephalus_bistrigiceps | Overall | LC | 27 |
| Birds | Acrocephalus_dumetorum | Facebook | LC | 91 |
| Birds | Acrocephalus_dumetorum | GBIF | LC | 610 |
| Birds | Acrocephalus_dumetorum | Overall | LC | 701 |
| Birds | Acrocephalus_orientalis | GBIF | LC | 1 |
| Birds | Acrocephalus_orientalis | Overall | LC | 1 |
| Birds | Acrocephalus_orinus | Facebook | DD | 15 |
| Birds | Acrocephalus_orinus | GBIF | DD | 1 |
| Birds | Acrocephalus_orinus | Overall | DD | 16 |
| Birds | Acrocephalus_stentoreus | Facebook | LC | 96 |
| Birds | Acrocephalus_stentoreus | GBIF | LC | 231 |
| Birds | Acrocephalus_stentoreus | Overall | LC | 327 |
| Birds | Actitis_hypoleucos | Facebook | LC | 90 |
| Birds | Actitis_hypoleucos | GBIF | LC | 440 |
| Birds | Actitis_hypoleucos | Overall | LC | 530 |
| Birds | Aegithina_tiphia | Facebook | LC | 326 |
| Birds | Aegithina_tiphia | GBIF | LC | 1092 |
| Birds | Aegithina_tiphia | Overall | LC | 1418 |
| Birds | Aegypius_monachus | Facebook | NT | 1 |
| Birds | Aegypius_monachus | Overall | NT | 1 |
| Birds | Aerodramus_brevirostris | Facebook | LC | 7 |
| Birds | Aerodramus_brevirostris | GBIF | LC | 10 |
| Birds | Aerodramus_brevirostris | Overall | LC | 17 |
| Birds | Aethopyga_siparaja | Facebook | LC | 98 |
| Birds | Aethopyga_siparaja | GBIF | LC | 116 |
| Birds | Aethopyga_siparaja | Overall | LC | 214 |
| Birds | Alauda_gulgula | Facebook | LC | 31 |
| Birds | Alauda_gulgula | GBIF | LC | 89 |
| Birds | Alauda_gulgula | Overall | LC | 120 |
| Birds | Alcedo_atthis | Facebook | LC | 719 |
| Birds | Alcedo_atthis | GBIF | LC | 1761 |
| Birds | Alcedo_atthis | Overall | LC | 2480 |
| Birds | Alcedo_meninting | Facebook | LC | 127 |
| Birds | Alcedo_meninting | GBIF | LC | 100 |
| Birds | Alcedo_meninting | Overall | LC | 227 |
| Birds | Alcippe_nipalensis | Facebook | NT | 9 |
| Birds | Alcippe_nipalensis | GBIF | NT | 18 |
| Birds | Alcippe_nipalensis | Overall | NT | 27 |
| Birds | Alcippe_poioicephala | Facebook | NT | 9 |
| Birds | Alcippe_poioicephala | GBIF | NT | 17 |
| Birds | Alcippe_poioicephala | Overall | NT | 26 |
| Birds | Alophoixus_flaveolus | Facebook | LC | 82 |
| Birds | Alophoixus_flaveolus | GBIF | LC | 150 |
| Birds | Alophoixus_flaveolus | Overall | LC | 232 |
| Birds | Amandava_amandava | Facebook | LC | 86 |
| Birds | Amandava_amandava | GBIF | LC | 118 |
| Birds | Amandava_amandava | Overall | LC | 204 |
| Birds | Amaurornis_phoenicurus | Facebook | LC | 89 |
| Birds | Amaurornis_phoenicurus | GBIF | LC | 965 |
| Birds | Amaurornis_phoenicurus | Overall | LC | 1054 |
| Birds | Anas_acuta | Facebook | LC | 81 |
| Birds | Anas_acuta | GBIF | LC | 135 |
| Birds | Anas_acuta | Overall | LC | 216 |
| Birds | Anas_crecca | Facebook | LC | 62 |
| Birds | Anas_crecca | GBIF | LC | 92 |
| Birds | Anas_crecca | Overall | LC | 154 |
| Birds | Anas_platyrhynchos | Facebook | LC | 17 |
| Birds | Anas_platyrhynchos | GBIF | LC | 21 |
| Birds | Anas_platyrhynchos | Overall | LC | 38 |
| Birds | Anas_poecilorhyncha | Facebook | LC | 64 |
| Birds | Anas_poecilorhyncha | GBIF | LC | 56 |
| Birds | Anas_poecilorhyncha | Overall | LC | 120 |
| Birds | Anastomus_oscitans | Facebook | LC | 87 |
| Birds | Anastomus_oscitans | GBIF | LC | 697 |
| Birds | Anastomus_oscitans | Overall | LC | 784 |
| Birds | Anhinga_melanogaster | Facebook | NT | 103 |
| Birds | Anhinga_melanogaster | GBIF | NT | 79 |
| Birds | Anhinga_melanogaster | Overall | NT | 182 |
| Birds | Anser_anser | Facebook | LC | 31 |
| Birds | Anser_anser | GBIF | LC | 17 |
| Birds | Anser_anser | Overall | LC | 48 |
| Birds | Anser_erythropus | Facebook | VU | 2 |
| Birds | Anser_erythropus | Overall | VU | 2 |
| Birds | Anser_indicus | Facebook | LC | 97 |
| Birds | Anser_indicus | GBIF | LC | 44 |
| Birds | Anser_indicus | Overall | LC | 141 |
| Birds | Anthracoceros_albirostris | Facebook | LC | 188 |
| Birds | Anthracoceros_albirostris | GBIF | LC | 79 |
| Birds | Anthracoceros_albirostris | Overall | LC | 267 |
| Birds | Anthreptes_singalensis | Facebook | LC | 99 |
| Birds | Anthreptes_singalensis | GBIF | LC | 3 |
| Birds | Anthreptes_singalensis | Overall | LC | 102 |
| Birds | Anthus_cervinus | Facebook | LC | 1 |
| Birds | Anthus_cervinus | GBIF | LC | 7 |
| Birds | Anthus_cervinus | Overall | LC | 8 |
| Birds | Anthus_hodgsoni | Facebook | LC | 86 |
| Birds | Anthus_hodgsoni | GBIF | LC | 245 |
| Birds | Anthus_hodgsoni | Overall | LC | 331 |
| Birds | Anthus_richardi | Facebook | LC | 29 |
| Birds | Anthus_richardi | GBIF | LC | 107 |
| Birds | Anthus_richardi | Overall | LC | 136 |
| Birds | Anthus_roseatus | Facebook | LC | 27 |
| Birds | Anthus_roseatus | GBIF | LC | 63 |
| Birds | Anthus_roseatus | Overall | LC | 90 |
| Birds | Anthus_rufulus | Facebook | LC | 100 |
| Birds | Anthus_rufulus | GBIF | LC | 433 |
| Birds | Anthus_rufulus | Overall | LC | 533 |
| Birds | Aplonis_panayensis | Facebook | LC | 48 |
| Birds | Aplonis_panayensis | GBIF | LC | 23 |
| Birds | Aplonis_panayensis | Overall | LC | 71 |
| Birds | Apus_nipalensis | Facebook | LC | 25 |
| Birds | Apus_nipalensis | GBIF | LC | 3264 |
| Birds | Apus_nipalensis | Overall | LC | 3289 |
| Birds | Apus_pacificus | Facebook | LC | 10 |
| Birds | Apus_pacificus | GBIF | LC | 27 |
| Birds | Apus_pacificus | Overall | LC | 37 |
| Birds | Aquila_heliaca | Facebook | VU | 7 |
| Birds | Aquila_heliaca | GBIF | VU | 5 |
| Birds | Aquila_heliaca | Overall | VU | 12 |
| Birds | Aquila_nipalensis | Facebook | LC | 26 |
| Birds | Aquila_nipalensis | GBIF | LC | 12 |
| Birds | Aquila_nipalensis | Overall | LC | 38 |
| Birds | Arachnothera_longirostra | Facebook | LC | 93 |
| Birds | Arachnothera_longirostra | GBIF | LC | 246 |
| Birds | Arachnothera_longirostra | Overall | LC | 339 |
| Birds | Arachnothera_magna | Facebook | LC | 41 |
| Birds | Arachnothera_magna | GBIF | LC | 31 |
| Birds | Arachnothera_magna | Overall | LC | 72 |
| Birds | Arborophila_atrogularis | Facebook | LC | 1 |
| Birds | Arborophila_atrogularis | GBIF | LC | 27 |
| Birds | Arborophila_atrogularis | Overall | LC | 28 |
| Birds | Ardea_alba | Facebook | LC | 278 |
| Birds | Ardea_alba | GBIF | LC | 680 |
| Birds | Ardea_alba | Overall | LC | 958 |
| Birds | Ardea_cinerea | Facebook | LC | 210 |
| Birds | Ardea_cinerea | GBIF | LC | 379 |
| Birds | Ardea_cinerea | Overall | LC | 589 |
| Birds | Ardea_goliath | GBIF | DD | 1 |
| Birds | Ardea_goliath | Overall | DD | 1 |
| Birds | Ardea_intermedia | Facebook | LC | 79 |
| Birds | Ardea_intermedia | GBIF | LC | 339 |
| Birds | Ardea_intermedia | Overall | LC | 418 |
| Birds | Ardea_purpurea | Facebook | LC | 118 |
| Birds | Ardea_purpurea | GBIF | LC | 88 |
| Birds | Ardea_purpurea | Overall | LC | 206 |
| Birds | Ardeola_bacchus | Facebook | LC | 4 |
| Birds | Ardeola_bacchus | GBIF | LC | 3 |
| Birds | Ardeola_bacchus | Overall | LC | 7 |
| Birds | Ardeola_grayii | Facebook | LC | 94 |
| Birds | Ardeola_grayii | GBIF | LC | 2664 |
| Birds | Ardeola_grayii | Overall | LC | 2758 |
| Birds | Arenaria_interpres | Facebook | LC | 17 |
| Birds | Arenaria_interpres | GBIF | LC | 16 |
| Birds | Arenaria_interpres | Overall | LC | 33 |
| Birds | Artamus_fuscus | Facebook | LC | 170 |
| Birds | Artamus_fuscus | GBIF | LC | 413 |
| Birds | Artamus_fuscus | Overall | LC | 583 |
| Birds | Asarcornis_scutulata | Facebook | LC | 1 |
| Birds | Asarcornis_scutulata | Overall | LC | 1 |
| Birds | Asio_flammeus | Facebook | LC | 66 |
| Birds | Asio_flammeus | GBIF | LC | 24 |
| Birds | Asio_flammeus | Overall | LC | 90 |
| Birds | Athene_brama | Facebook | LC | 93 |
| Birds | Athene_brama | GBIF | LC | 407 |
| Birds | Athene_brama | Overall | LC | 500 |
| Birds | Aviceda_jerdoni | Facebook | LC | 61 |
| Birds | Aviceda_jerdoni | GBIF | LC | 24 |
| Birds | Aviceda_jerdoni | Overall | LC | 85 |
| Birds | Aviceda_leuphotes | Facebook | LC | 41 |
| Birds | Aviceda_leuphotes | GBIF | LC | 30 |
| Birds | Aviceda_leuphotes | Overall | LC | 71 |
| Birds | Aythya_baeri | Facebook | CR | 6 |
| Birds | Aythya_baeri | GBIF | CR | 3 |
| Birds | Aythya_baeri | Overall | CR | 9 |
| Birds | Aythya_ferina | Facebook | LC | 19 |
| Birds | Aythya_ferina | GBIF | LC | 43 |
| Birds | Aythya_ferina | Overall | LC | 62 |
| Birds | Aythya_fuligula | Facebook | LC | 58 |
| Birds | Aythya_fuligula | GBIF | LC | 52 |
| Birds | Aythya_fuligula | Overall | LC | 110 |
| Birds | Aythya_marila | GBIF | DD | 5 |
| Birds | Aythya_marila | Overall | DD | 5 |
| Birds | Aythya_nyroca | Facebook | NT | 35 |
| Birds | Aythya_nyroca | GBIF | NT | 88 |
| Birds | Aythya_nyroca | Overall | NT | 123 |
| Birds | Batrachostomus_hodgsoni | GBIF | DD | 1 |
| Birds | Batrachostomus_hodgsoni | Overall | DD | 1 |
| Birds | Blythipicus_pyrrhotis | Facebook | LC | 6 |
| Birds | Blythipicus_pyrrhotis | GBIF | LC | 12 |
| Birds | Blythipicus_pyrrhotis | Overall | LC | 18 |
| Birds | Botaurus_stellaris | Facebook | LC | 2 |
| Birds | Botaurus_stellaris | GBIF | LC | 2 |
| Birds | Botaurus_stellaris | Overall | LC | 4 |
| Birds | Brachypteryx_leucophrys | Facebook | LC | 10 |
| Birds | Brachypteryx_leucophrys | Overall | LC | 10 |
| Birds | Bradypterus_davidi | Facebook | LC | 3 |
| Birds | Bradypterus_davidi | Overall | LC | 3 |
| Birds | Bradypterus_thoracicus | Facebook | LC | 5 |
| Birds | Bradypterus_thoracicus | Overall | LC | 5 |
| Birds | Bubo_coromandus | Facebook | LC | 8 |
| Birds | Bubo_coromandus | GBIF | LC | 15 |
| Birds | Bubo_coromandus | Overall | LC | 23 |
| Birds | Bubo_nipalensis | Facebook | LC | 6 |
| Birds | Bubo_nipalensis | GBIF | LC | 1 |
| Birds | Bubo_nipalensis | Overall | LC | 7 |
| Birds | Bubulcus_ibis | Facebook | LC | 371 |
| Birds | Bubulcus_ibis | GBIF | LC | 1241 |
| Birds | Bubulcus_ibis | Overall | LC | 1612 |
| Birds | Buceros_bicornis | Facebook | VU | 8 |
| Birds | Buceros_bicornis | GBIF | VU | 8 |
| Birds | Buceros_bicornis | Overall | VU | 16 |
| Birds | Burhinus_indicus | Facebook | LC | 32 |
| Birds | Burhinus_indicus | GBIF | LC | 35 |
| Birds | Burhinus_indicus | Overall | LC | 67 |
| Birds | Butastur_teesa | Facebook | LC | 28 |
| Birds | Butastur_teesa | GBIF | LC | 27 |
| Birds | Butastur_teesa | Overall | LC | 55 |
| Birds | Buteo_japonicus | Facebook | LC | 67 |
| Birds | Buteo_japonicus | GBIF | LC | 17 |
| Birds | Buteo_japonicus | Overall | LC | 84 |
| Birds | Buteo_rufinus | Facebook | LC | 185 |
| Birds | Buteo_rufinus | GBIF | LC | 160 |
| Birds | Buteo_rufinus | Overall | LC | 345 |
| Birds | Butorides_striata | Facebook | LC | 99 |
| Birds | Butorides_striata | GBIF | LC | 578 |
| Birds | Butorides_striata | Overall | LC | 677 |
| Birds | Cacomantis_merulinus | Facebook | LC | 97 |
| Birds | Cacomantis_merulinus | GBIF | LC | 277 |
| Birds | Cacomantis_merulinus | Overall | LC | 277 |
| Birds | Cacomantis_merulinus | Overall | LC | 97 |
| Birds | Cacomantis_passerinus | Facebook | LC | 13 |
| Birds | Cacomantis_passerinus | GBIF | LC | 2 |
| Birds | Cacomantis_passerinus | Overall | LC | 15 |
| Birds | Cacomantis_sonneratii | Facebook | LC | 17 |
| Birds | Cacomantis_sonneratii | GBIF | LC | 21 |
| Birds | Cacomantis_sonneratii | Overall | LC | 38 |
| Birds | Calandrella_brachydactyla | Facebook | DD | 1 |
| Birds | Calandrella_brachydactyla | GBIF | DD | 14 |
| Birds | Calandrella_brachydactyla | Overall | DD | 15 |
| Birds | Calandrella_raytal | GBIF | LC | 147 |
| Birds | Calandrella_raytal | Overall | LC | 147 |
| Birds | Calidris_alba | Facebook | LC | 26 |
| Birds | Calidris_alba | GBIF | LC | 11 |
| Birds | Calidris_alba | Overall | LC | 37 |
| Birds | Calidris_alpina | Facebook | LC | 42 |
| Birds | Calidris_alpina | GBIF | LC | 39 |
| Birds | Calidris_alpina | Overall | LC | 81 |
| Birds | Calidris_canutus | Facebook | NT | 5 |
| Birds | Calidris_canutus | GBIF | NT | 8 |
| Birds | Calidris_canutus | Overall | NT | 13 |
| Birds | Calidris_falcinellus | Facebook | LC | 16 |
| Birds | Calidris_falcinellus | GBIF | LC | 17 |
| Birds | Calidris_falcinellus | Overall | LC | 33 |
| Birds | Calidris_ferruginea | Facebook | LC | 29 |
| Birds | Calidris_ferruginea | GBIF | LC | 31 |
| Birds | Calidris_ferruginea | Overall | LC | 60 |
| Birds | Calidris_minuta | Facebook | LC | 59 |
| Birds | Calidris_minuta | GBIF | LC | 136 |
| Birds | Calidris_minuta | Overall | LC | 195 |
| Birds | Calidris_pugnax | Facebook | LC | 74 |
| Birds | Calidris_pugnax | GBIF | LC | 73 |
| Birds | Calidris_pugnax | Overall | LC | 147 |
| Birds | Calidris_pygmaea | Facebook | CR | 8 |
| Birds | Calidris_pygmaea | GBIF | CR | 2 |
| Birds | Calidris_pygmaea | Overall | CR | 10 |
| Birds | Calidris_ruficollis | GBIF | LC | 7 |
| Birds | Calidris_ruficollis | Overall | LC | 7 |
| Birds | Calidris_subminuta | Facebook | NT | 1 |
| Birds | Calidris_subminuta | GBIF | NT | 1 |
| Birds | Calidris_subminuta | Overall | NT | 2 |
| Birds | Calidris_temminckii | Facebook | LC | 95 |
| Birds | Calidris_temminckii | GBIF | LC | 170 |
| Birds | Calidris_temminckii | Overall | LC | 265 |
| Birds | Calidris_tenuirostris | Facebook | EN | 15 |
| Birds | Calidris_tenuirostris | GBIF | EN | 15 |
| Birds | Calidris_tenuirostris | Overall | EN | 30 |
| Birds | Caprimulgus_affinis | GBIF | DD | 8 |
| Birds | Caprimulgus_affinis | Overall | DD | 8 |
| Birds | Caprimulgus_asiaticus | Facebook | LC | 12 |
| Birds | Caprimulgus_asiaticus | GBIF | LC | 10 |
| Birds | Caprimulgus_asiaticus | Overall | LC | 22 |
| Birds | Caprimulgus_jotaka | Facebook | LC | 1 |
| Birds | Caprimulgus_jotaka | GBIF | LC | 1 |
| Birds | Caprimulgus_jotaka | Overall | LC | 2 |
| Birds | Caprimulgus_macrurus | Facebook | LC | 95 |
| Birds | Caprimulgus_macrurus | GBIF | LC | 115 |
| Birds | Caprimulgus_macrurus | Overall | LC | 210 |
| Birds | Carpodacus_erythrinus | Facebook | LC | 32 |
| Birds | Carpodacus_erythrinus | GBIF | LC | 21 |
| Birds | Carpodacus_erythrinus | Overall | LC | 53 |
| Birds | Celeus_brachyurus | Facebook | LC | 139 |
| Birds | Celeus_brachyurus | Overall | LC | 139 |
| Birds | Centropus_bengalensis | Facebook | LC | 54 |
| Birds | Centropus_bengalensis | GBIF | LC | 131 |
| Birds | Centropus_bengalensis | Overall | LC | 185 |
| Birds | Centropus_sinensis | Facebook | LC | 98 |
| Birds | Centropus_sinensis | GBIF | LC | 804 |
| Birds | Centropus_sinensis | Overall | LC | 902 |
| Birds | Ceryle_rudis | Facebook | LC | 342 |
| Birds | Ceryle_rudis | GBIF | LC | 549 |
| Birds | Ceryle_rudis | Overall | LC | 891 |
| Birds | Ceyx_erithaca | Facebook | EN | 11 |
| Birds | Ceyx_erithaca | GBIF | EN | 10 |
| Birds | Ceyx_erithaca | Overall | EN | 21 |
| Birds | Chaetornis_striata | Facebook | EN | 21 |
| Birds | Chaetornis_striata | GBIF | EN | 4 |
| Birds | Chaetornis_striata | Overall | EN | 25 |
| Birds | Chaimarrornis_leucocephalus | Facebook | LC | 18 |
| Birds | Chaimarrornis_leucocephalus | GBIF | LC | 2 |
| Birds | Chaimarrornis_leucocephalus | Overall | LC | 20 |
| Birds | Chalcophaps_indica | Facebook | LC | 91 |
| Birds | Chalcophaps_indica | GBIF | LC | 137 |
| Birds | Chalcophaps_indica | Overall | LC | 228 |
| Birds | Charadrius_alexandrinus | Facebook | LC | 72 |
| Birds | Charadrius_alexandrinus | GBIF | LC | 116 |
| Birds | Charadrius_alexandrinus | Overall | LC | 188 |
| Birds | Charadrius_dubius | Facebook | LC | 97 |
| Birds | Charadrius_dubius | GBIF | LC | 354 |
| Birds | Charadrius_dubius | Overall | LC | 451 |
| Birds | Charadrius_leschenaultii | Facebook | LC | 30 |
| Birds | Charadrius_leschenaultii | GBIF | LC | 25 |
| Birds | Charadrius_leschenaultii | Overall | LC | 55 |
| Birds | Charadrius_mongolus | Facebook | LC | 67 |
| Birds | Charadrius_mongolus | GBIF | LC | 38 |
| Birds | Charadrius_mongolus | Overall | LC | 105 |
| Birds | Chlidonias_hybrida | Facebook | LC | 98 |
| Birds | Chlidonias_hybrida | GBIF | LC | 135 |
| Birds | Chlidonias_hybrida | Overall | LC | 233 |
| Birds | Chlidonias_leucopterus | Facebook | DD | 10 |
| Birds | Chlidonias_leucopterus | GBIF | DD | 2 |
| Birds | Chlidonias_leucopterus | Overall | DD | 12 |
| Birds | Chloropsis_aurifrons | Facebook | LC | 96 |
| Birds | Chloropsis_aurifrons | GBIF | LC | 175 |
| Birds | Chloropsis_aurifrons | Overall | LC | 271 |
| Birds | Chloropsis_cochinchinensis | Facebook | LC | 11 |
| Birds | Chloropsis_cochinchinensis | GBIF | LC | 10 |
| Birds | Chloropsis_cochinchinensis | Overall | LC | 21 |
| Birds | Chloropsis_hardwickii | Facebook | LC | 20 |
| Birds | Chloropsis_hardwickii | GBIF | LC | 5 |
| Birds | Chloropsis_hardwickii | Overall | LC | 25 |
| Birds | Chrysococcyx_maculatus | Facebook | LC | 15 |
| Birds | Chrysococcyx_maculatus | GBIF | LC | 1 |
| Birds | Chrysococcyx_maculatus | Overall | LC | 16 |
| Birds | Chrysococcyx_xanthorhynchus | Facebook | LC | 30 |
| Birds | Chrysococcyx_xanthorhynchus | GBIF | LC | 19 |
| Birds | Chrysococcyx_xanthorhynchus | Overall | LC | 49 |
| Birds | Chrysocolaptes_guttacristatus | Facebook | LC | 84 |
| Birds | Chrysocolaptes_guttacristatus | GBIF | LC | 131 |
| Birds | Chrysocolaptes_guttacristatus | Overall | LC | 215 |
| Birds | Chrysomma_sinense | Facebook | VU | 23 |
| Birds | Chrysomma_sinense | GBIF | VU | 12 |
| Birds | Chrysomma_sinense | Overall | VU | 35 |
| Birds | Ciconia_episcopus | Facebook | CR | 11 |
| Birds | Ciconia_episcopus | GBIF | CR | 20 |
| Birds | Ciconia_episcopus | Overall | CR | 31 |
| Birds | Ciconia_nigra | Facebook | VU | 62 |
| Birds | Ciconia_nigra | GBIF | VU | 44 |
| Birds | Ciconia_nigra | Overall | VU | 106 |
| Birds | Cinclidium_leucurum | Facebook | LC | 31 |
| Birds | Cinclidium_leucurum | Overall | LC | 31 |
| Birds | Cinnyris_asiaticus | Facebook | LC | 81 |
| Birds | Cinnyris_asiaticus | GBIF | LC | 1739 |
| Birds | Cinnyris_asiaticus | Overall | LC | 1820 |
| Birds | Circaetus_gallicus | Facebook | LC | 8 |
| Birds | Circaetus_gallicus | GBIF | LC | 4 |
| Birds | Circaetus_gallicus | Overall | LC | 12 |
| Birds | Circus_aeruginosus | Facebook | LC | 32 |
| Birds | Circus_aeruginosus | GBIF | LC | 43 |
| Birds | Circus_aeruginosus | Overall | LC | 75 |
| Birds | Circus_cyaneus | Facebook | DD | 27 |
| Birds | Circus_cyaneus | GBIF | DD | 30 |
| Birds | Circus_cyaneus | Overall | DD | 57 |
| Birds | Circus_macrourus | Facebook | DD | 6 |
| Birds | Circus_macrourus | GBIF | DD | 1 |
| Birds | Circus_macrourus | Overall | DD | 7 |
| Birds | Circus_melanoleucos | Facebook | LC | 65 |
| Birds | Circus_melanoleucos | GBIF | LC | 69 |
| Birds | Circus_melanoleucos | Overall | LC | 134 |
| Birds | Circus_spilonotus | Facebook | LC | 54 |
| Birds | Circus_spilonotus | GBIF | LC | 33 |
| Birds | Circus_spilonotus | Overall | LC | 87 |
| Birds | Cissa_chinensis | Facebook | LC | 85 |
| Birds | Cissa_chinensis | GBIF | LC | 49 |
| Birds | Cissa_chinensis | Overall | LC | 134 |
| Birds | Cisticola_exilis | Facebook | LC | 5 |
| Birds | Cisticola_exilis | GBIF | LC | 2 |
| Birds | Cisticola_exilis | Overall | LC | 7 |
| Birds | Cisticola_juncidis | Facebook | LC | 92 |
| Birds | Cisticola_juncidis | GBIF | LC | 537 |
| Birds | Cisticola_juncidis | Overall | LC | 629 |
| Birds | Clamator_coromandus | Facebook | LC | 33 |
| Birds | Clamator_coromandus | GBIF | LC | 14 |
| Birds | Clamator_coromandus | Overall | LC | 47 |
| Birds | Clamator_jacobinus | Facebook | LC | 106 |
| Birds | Clamator_jacobinus | GBIF | LC | 98 |
| Birds | Clamator_jacobinus | Overall | LC | 204 |
| Birds | Clanga_clanga | Facebook | VU | 65 |
| Birds | Clanga_clanga | GBIF | VU | 56 |
| Birds | Clanga_clanga | Overall | VU | 121 |
| Birds | Clanga_hastata | Facebook | EN | 54 |
| Birds | Clanga_hastata | GBIF | EN | 81 |
| Birds | Clanga_hastata | Overall | EN | 135 |
| Birds | Columba_livia | Facebook | LC | 42 |
| Birds | Columba_livia | GBIF | LC | 4213 |
| Birds | Columba_livia | Overall | LC | 4255 |
| Birds | Columba_punicea_Blyth | Facebook | LC | 1 |
| Birds | Columba_punicea_Blyth | GBIF | LC | 3 |
| Birds | Columba_punicea_Blyth | Overall | LC | 4 |
| Birds | Copsychus_malabaricus | Facebook | LC | 38 |
| Birds | Copsychus_malabaricus | GBIF | LC | 125 |
| Birds | Copsychus_malabaricus | Overall | LC | 163 |
| Birds | Coracias_affinis | Facebook | DD | 65 |
| Birds | Coracias_affinis | GBIF | DD | 40 |
| Birds | Coracias_affinis | Overall | DD | 105 |
| Birds | Coracias_benghalensis | Facebook | LC | 289 |
| Birds | Coracias_benghalensis | GBIF | LC | 32 |
| Birds | Coracias_benghalensis | Overall | LC | 321 |
| Birds | Coracina_macei | Facebook | LC | 95 |
| Birds | Coracina_macei | GBIF | LC | 127 |
| Birds | Coracina_macei | Overall | LC | 222 |
| Birds | Coracina_melanoptera | Facebook | LC | 32 |
| Birds | Coracina_melanoptera | GBIF | LC | 147 |
| Birds | Coracina_melanoptera | Overall | LC | 179 |
| Birds | Coracina_melaschistos | Facebook | LC | 4 |
| Birds | Coracina_melaschistos | GBIF | LC | 69 |
| Birds | Coracina_melaschistos | Overall | LC | 73 |
| Birds | Corvus_levaillantii | Facebook | LC | 42 |
| Birds | Corvus_levaillantii | GBIF | LC | 6 |
| Birds | Corvus_levaillantii | Overall | LC | 48 |
| Birds | Corvus_splendens | Facebook | LC | 64 |
| Birds | Corvus_splendens | GBIF | LC | 5576 |
| Birds | Corvus_splendens | Overall | LC | 5640 |
| Birds | Coturnix_chinensis | GBIF | DD | 10 |
| Birds | Coturnix_chinensis | Overall | DD | 10 |
| Birds | Coturnix_coromandelica | GBIF | DD | 22 |
| Birds | Coturnix_coromandelica | Overall | DD | 22 |
| Birds | Coturnix_coturnix | GBIF | DD | 7 |
| Birds | Coturnix_coturnix | Overall | DD | 7 |
| Birds | Cuculus_canorus | GBIF | DD | 52 |
| Birds | Cuculus_canorus | Overall | DD | 52 |
| Birds | Cuculus_micropterus | Facebook | LC | 84 |
| Birds | Cuculus_micropterus | GBIF | LC | 198 |
| Birds | Cuculus_micropterus | Overall | LC | 282 |
| Birds | Culicicapa_ceylonensis | Facebook | LC | 123 |
| Birds | Culicicapa_ceylonensis | GBIF | LC | 173 |
| Birds | Culicicapa_ceylonensis | Overall | LC | 296 |
| Birds | Cyornis_poliogenys | Facebook | LC | 63 |
| Birds | Cyornis_poliogenys | GBIF | LC | 82 |
| Birds | Cyornis_poliogenys | Overall | LC | 145 |
| Birds | Cyornis_rubeculoides | Facebook | LC | 94 |
| Birds | Cyornis_rubeculoides | GBIF | LC | 77 |
| Birds | Cyornis_rubeculoides | Overall | LC | 171 |
| Birds | Cyornis_unicolor | GBIF | LC | 5 |
| Birds | Cyornis_unicolor | Overall | LC | 5 |
| Birds | Cyornis_unicolor_Blyth | Facebook | LC | 2 |
| Birds | Cyornis_unicolor_Blyth | GBIF | LC | 5 |
| Birds | Cyornis_unicolor_Blyth | Overall | LC | 7 |
| Birds | Cypsiurus_balasiensis | Facebook | LC | 36 |
| Birds | Cypsiurus_balasiensis | GBIF | LC | 2311 |
| Birds | Cypsiurus_balasiensis | Overall | LC | 2347 |
| Birds | Delichon_nipalensis | Facebook | LC | 5 |
| Birds | Delichon_nipalensis | Overall | LC | 5 |
| Birds | Dendrocitta_formosae | Facebook | LC | 4 |
| Birds | Dendrocitta_formosae | GBIF | LC | 44 |
| Birds | Dendrocitta_formosae | Overall | LC | 48 |
| Birds | Dendrocitta_vagabunda | Facebook | LC | 81 |
| Birds | Dendrocitta_vagabunda | GBIF | LC | 1475 |
| Birds | Dendrocitta_vagabunda | Overall | LC | 1556 |
| Birds | Dendrocopos_canicapillus | Facebook | LC | 102 |
| Birds | Dendrocopos_canicapillus | GBIF | LC | 1 |
| Birds | Dendrocopos_canicapillus | Overall | LC | 103 |
| Birds | Dendrocopos_macei | Facebook | LC | 291 |
| Birds | Dendrocopos_macei | GBIF | LC | 1180 |
| Birds | Dendrocopos_macei | Overall | LC | 1471 |
| Birds | Dendrocygna_bicolor | Facebook | LC | 22 |
| Birds | Dendrocygna_bicolor | GBIF | LC | 35 |
| Birds | Dendrocygna_bicolor | Overall | LC | 57 |
| Birds | Dendrocygna_javanica | Facebook | LC | 93 |
| Birds | Dendrocygna_javanica | GBIF | LC | 931 |
| Birds | Dendrocygna_javanica | Overall | LC | 1024 |
| Birds | Dicaeum_agile | Facebook | LC | 16 |
| Birds | Dicaeum_agile | GBIF | LC | 10 |
| Birds | Dicaeum_agile | Overall | LC | 26 |
| Birds | Dicaeum_chrysorrheum | Facebook | LC | 31 |
| Birds | Dicaeum_chrysorrheum | GBIF | LC | 9 |
| Birds | Dicaeum_chrysorrheum | Overall | LC | 40 |
| Birds | Dicaeum_concolor | Facebook | LC | 16 |
| Birds | Dicaeum_concolor | GBIF | LC | 1 |
| Birds | Dicaeum_concolor | Overall | LC | 17 |
| Birds | Dicaeum_cruentatum | Facebook | LC | 97 |
| Birds | Dicaeum_cruentatum | GBIF | LC | 231 |
| Birds | Dicaeum_cruentatum | Overall | LC | 328 |
| Birds | Dicaeum_erythrorhynchos | Facebook | LC | 97 |
| Birds | Dicaeum_erythrorhynchos | GBIF | LC | 599 |
| Birds | Dicaeum_erythrorhynchos | Overall | LC | 696 |
| Birds | Dicaeum_trigonostigma | Facebook | LC | 29 |
| Birds | Dicaeum_trigonostigma | GBIF | LC | 12 |
| Birds | Dicaeum_trigonostigma | Overall | LC | 41 |
| Birds | Dicrurus_aeneus | Facebook | LC | 84 |
| Birds | Dicrurus_aeneus | GBIF | LC | 470 |
| Birds | Dicrurus_aeneus | Overall | LC | 554 |
| Birds | Dicrurus_annectans | Facebook | DD | 3 |
| Birds | Dicrurus_annectans | Overall | DD | 3 |
| Birds | Dicrurus_hottentottus | Facebook | LC | 87 |
| Birds | Dicrurus_hottentottus | GBIF | LC | 211 |
| Birds | Dicrurus_hottentottus | Overall | LC | 298 |
| Birds | Dicrurus_leucophaeus | Facebook | LC | 90 |
| Birds | Dicrurus_leucophaeus | GBIF | LC | 241 |
| Birds | Dicrurus_leucophaeus | Overall | LC | 331 |
| Birds | Dicrurus_macrocercus | Facebook | LC | 94 |
| Birds | Dicrurus_macrocercus | GBIF | LC | 3051 |
| Birds | Dicrurus_macrocercus | Overall | LC | 3145 |
| Birds | Dicrurus_paradiseus | Facebook | LC | 94 |
| Birds | Dicrurus_paradiseus | GBIF | LC | 186 |
| Birds | Dicrurus_paradiseus | Overall | LC | 280 |
| Birds | Dicrurus_remifer | Facebook | LC | 49 |
| Birds | Dicrurus_remifer | GBIF | LC | 48 |
| Birds | Dicrurus_remifer | Overall | LC | 97 |
| Birds | Dinopium_benghalense | Facebook | LC | 379 |
| Birds | Dinopium_benghalense | GBIF | LC | 2302 |
| Birds | Dinopium_benghalense | Overall | LC | 2681 |
| Birds | Dinopium_javanense | Facebook | LC | 13 |
| Birds | Dinopium_javanense | GBIF | LC | 4 |
| Birds | Dinopium_javanense | Overall | LC | 17 |
| Birds | Ducula_aenea | Facebook | LC | 31 |
| Birds | Ducula_aenea | GBIF | LC | 58 |
| Birds | Ducula_aenea | Overall | LC | 89 |
| Birds | Ducula_badia | Facebook | LC | 9 |
| Birds | Ducula_badia | GBIF | LC | 7 |
| Birds | Ducula_badia | Overall | LC | 16 |
| Birds | Egretta_garzetta | Facebook | LC | 93 |
| Birds | Egretta_garzetta | GBIF | LC | 1122 |
| Birds | Egretta_garzetta | Overall | LC | 1215 |
| Birds | Elanus_caeruleus | Facebook | LC | 142 |
| Birds | Elanus_caeruleus | GBIF | LC | 342 |
| Birds | Elanus_caeruleus | Overall | LC | 484 |
| Birds | Emberiza_aureola | Facebook | VU | 7 |
| Birds | Emberiza_aureola | GBIF | VU | 6 |
| Birds | Emberiza_aureola | Overall | VU | 13 |
| Birds | Emberiza_fucata | Facebook | LC | 20 |
| Birds | Emberiza_fucata | GBIF | LC | 23 |
| Birds | Emberiza_fucata | Overall | LC | 43 |
| Birds | Emberiza_pusilla | Facebook | LC | 20 |
| Birds | Emberiza_pusilla | GBIF | LC | 9 |
| Birds | Emberiza_pusilla | Overall | LC | 29 |
| Birds | Emberiza_spodocephala | Facebook | LC | 13 |
| Birds | Emberiza_spodocephala | GBIF | LC | 20 |
| Birds | Emberiza_spodocephala | Overall | LC | 33 |
| Birds | Enicurus_immaculatus | Facebook | LC | 40 |
| Birds | Enicurus_immaculatus | GBIF | LC | 39 |
| Birds | Enicurus_immaculatus | Overall | LC | 79 |
| Birds | Ephippiorhynchus_asiaticus | Facebook | EN | 16 |
| Birds | Ephippiorhynchus_asiaticus | Overall | EN | 16 |
| Birds | Eremopterix_grisea | Facebook | LC | 5 |
| Birds | Eremopterix_grisea | Overall | LC | 5 |
| Birds | Erpornis_zantholeuca | Facebook | LC | 14 |
| Birds | Erpornis_zantholeuca | GBIF | LC | 16 |
| Birds | Erpornis_zantholeuca | Overall | LC | 30 |
| Birds | Esacus_recurvirostris | Facebook | NT | 30 |
| Birds | Esacus_recurvirostris | GBIF | NT | 7 |
| Birds | Esacus_recurvirostris | Overall | NT | 37 |
| Birds | Eudynamys_scolopaceus | Facebook | LC | 110 |
| Birds | Eudynamys_scolopaceus | GBIF | LC | 2063 |
| Birds | Eudynamys_scolopaceus | Overall | LC | 2173 |
| Birds | Eumyias_thalassina | Facebook | LC | 138 |
| Birds | Eumyias_thalassina | Overall | LC | 138 |
| Birds | Eurystomus_orientalis | Facebook | LC | 43 |
| Birds | Eurystomus_orientalis | GBIF | LC | 31 |
| Birds | Eurystomus_orientalis | Overall | LC | 74 |
| Birds | Falco_amurensis | Facebook | LC | 48 |
| Birds | Falco_amurensis | GBIF | LC | 62 |
| Birds | Falco_amurensis | Overall | LC | 110 |
| Birds | Falco_chicquera | Facebook | LC | 71 |
| Birds | Falco_chicquera | GBIF | LC | 128 |
| Birds | Falco_chicquera | Overall | LC | 199 |
| Birds | Falco_jugger | GBIF | VU | 3 |
| Birds | Falco_jugger | Overall | VU | 3 |
| Birds | Falco_peregrinus | Facebook | LC | 137 |
| Birds | Falco_peregrinus | GBIF | LC | 332 |
| Birds | Falco_peregrinus | Overall | LC | 469 |
| Birds | Falco_subbuteo | Facebook | LC | 31 |
| Birds | Falco_subbuteo | GBIF | LC | 32 |
| Birds | Falco_subbuteo | Overall | LC | 63 |
| Birds | Falco_tinnunculus | Facebook | LC | 242 |
| Birds | Falco_tinnunculus | GBIF | LC | 415 |
| Birds | Falco_tinnunculus | Overall | LC | 657 |
| Birds | Ficedula_albicilla | Facebook | LC | 379 |
| Birds | Ficedula_albicilla | GBIF | LC | 931 |
| Birds | Ficedula_albicilla | Overall | LC | 1310 |
| Birds | Ficedula_hyperythra | Facebook | LC | 41 |
| Birds | Ficedula_hyperythra | GBIF | LC | 41 |
| Birds | Ficedula_hyperythra | Overall | LC | 82 |
| Birds | Ficedula_strophiata | Facebook | NT | 1 |
| Birds | Ficedula_strophiata | GBIF | NT | 3 |
| Birds | Ficedula_strophiata | Overall | NT | 4 |
| Birds | Ficedula_westermanni | Facebook | LC | 42 |
| Birds | Ficedula_westermanni | GBIF | LC | 42 |
| Birds | Ficedula_westermanni | Overall | LC | 84 |
| Birds | Francolinus_francolinus | Facebook | EN | 24 |
| Birds | Francolinus_francolinus | GBIF | EN | 22 |
| Birds | Francolinus_francolinus | Overall | EN | 46 |
| Birds | Francolinus_pondicerianus | Facebook | RE | 11 |
| Birds | Francolinus_pondicerianus | GBIF | RE | 29 |
| Birds | Francolinus_pondicerianus | Overall | RE | 40 |
| Birds | Fulica_atra | Facebook | LC | 70 |
| Birds | Fulica_atra | GBIF | LC | 59 |
| Birds | Fulica_atra | Overall | LC | 129 |
| Birds | Gallicrex_cinerea | Facebook | LC | 55 |
| Birds | Gallicrex_cinerea | GBIF | LC | 46 |
| Birds | Gallicrex_cinerea | Overall | LC | 101 |
| Birds | Gallinago_gallinago | Facebook | LC | 68 |
| Birds | Gallinago_gallinago | GBIF | LC | 138 |
| Birds | Gallinago_gallinago | Overall | LC | 206 |
| Birds | Gallinago_stenura | Facebook | LC | 33 |
| Birds | Gallinago_stenura | GBIF | LC | 99 |
| Birds | Gallinago_stenura | Overall | LC | 132 |
| Birds | Gallinula_chloropus | Facebook | LC | 89 |
| Birds | Gallinula_chloropus | GBIF | LC | 237 |
| Birds | Gallinula_chloropus | Overall | LC | 326 |
| Birds | Gallus_gallus | Facebook | LC | 63 |
| Birds | Gallus_gallus | GBIF | LC | 143 |
| Birds | Gallus_gallus | Overall | LC | 206 |
| Birds | Gampsorhynchus_rufulus | Facebook | LC | 1 |
| Birds | Gampsorhynchus_rufulus | GBIF | LC | 3 |
| Birds | Gampsorhynchus_rufulus | Overall | LC | 4 |
| Birds | Garrulax_galbanus | Facebook | LC | 1 |
| Birds | Garrulax_galbanus | Overall | LC | 1 |
| Birds | Garrulax_leucolophus | Facebook | LC | 11 |
| Birds | Garrulax_leucolophus | GBIF | LC | 11 |
| Birds | Garrulax_leucolophus | Overall | LC | 22 |
| Birds | Garrulax_monileger | Facebook | LC | 34 |
| Birds | Garrulax_monileger | GBIF | LC | 29 |
| Birds | Garrulax_monileger | Overall | LC | 63 |
| Birds | Garrulax_pectoralis | Facebook | LC | 45 |
| Birds | Garrulax_pectoralis | GBIF | LC | 4 |
| Birds | Garrulax_pectoralis | Overall | LC | 49 |
| Birds | Garrulax_ruficollis | Facebook | LC | 51 |
| Birds | Garrulax_ruficollis | Overall | LC | 51 |
| Birds | Gecinulus_grantia | GBIF | LC | 1 |
| Birds | Gecinulus_grantia | Overall | LC | 1 |
| Birds | Gelochelidon_nilotica | GBIF | LC | 26 |
| Birds | Gelochelidon_nilotica | Overall | LC | 26 |
| Birds | Glareola_lactea | Facebook | LC | 149 |
| Birds | Glareola_lactea | GBIF | LC | 118 |
| Birds | Glareola_lactea | Overall | LC | 267 |
| Birds | Glareola_maldivarum | Facebook | LC | 46 |
| Birds | Glareola_maldivarum | GBIF | LC | 36 |
| Birds | Glareola_maldivarum | Overall | LC | 82 |
| Birds | Glaucidium_cuculoides | Facebook | LC | 75 |
| Birds | Glaucidium_cuculoides | GBIF | LC | 100 |
| Birds | Glaucidium_cuculoides | Overall | LC | 175 |
| Birds | Gorsachius_melanolophus | Facebook | LC | 26 |
| Birds | Gorsachius_melanolophus | GBIF | LC | 3 |
| Birds | Gorsachius_melanolophus | Overall | LC | 29 |
| Birds | Gyps_bengalensis | Facebook | CR | 18 |
| Birds | Gyps_bengalensis | GBIF | CR | 11 |
| Birds | Gyps_bengalensis | Overall | CR | 29 |
| Birds | Gyps_himalayensis | Facebook | LC | 20 |
| Birds | Gyps_himalayensis | GBIF | LC | 12 |
| Birds | Gyps_himalayensis | Overall | LC | 32 |
| Birds | Haematopus_ostralegus | Facebook | VU | 4 |
| Birds | Haematopus_ostralegus | GBIF | VU | 1 |
| Birds | Haematopus_ostralegus | Overall | VU | 5 |
| Birds | Halcyon_coromanda | Facebook | LC | 61 |
| Birds | Halcyon_coromanda | GBIF | LC | 18 |
| Birds | Halcyon_coromanda | Overall | LC | 79 |
| Birds | Halcyon_pileata | Facebook | LC | 116 |
| Birds | Halcyon_pileata | GBIF | LC | 53 |
| Birds | Halcyon_pileata | Overall | LC | 169 |
| Birds | Halcyon_smyrnensis | Facebook | LC | 613 |
| Birds | Halcyon_smyrnensis | GBIF | LC | 2481 |
| Birds | Halcyon_smyrnensis | Overall | LC | 3094 |
| Birds | Haliaeetus_leucogaster | Facebook | LC | 75 |
| Birds | Haliaeetus_leucogaster | GBIF | LC | 21 |
| Birds | Haliaeetus_leucogaster | Overall | LC | 96 |
| Birds | Haliaeetus_leucoryphus | Facebook | EN | 78 |
| Birds | Haliaeetus_leucoryphus | GBIF | EN | 52 |
| Birds | Haliaeetus_leucoryphus | Overall | EN | 130 |
| Birds | Haliastur_indus | Facebook | LC | 259 |
| Birds | Haliastur_indus | GBIF | LC | 1619 |
| Birds | Haliastur_indus | Overall | LC | 1878 |
| Birds | Harpactes_erythrocephalus | Facebook | LC | 41 |
| Birds | Harpactes_erythrocephalus | GBIF | LC | 24 |
| Birds | Harpactes_erythrocephalus | Overall | LC | 65 |
| Birds | Heliopais_personatus | Facebook | EN | 37 |
| Birds | Heliopais_personatus | GBIF | EN | 8 |
| Birds | Heliopais_personatus | Overall | EN | 45 |
| Birds | Hemicircus_canente | Facebook | LC | 1 |
| Birds | Hemicircus_canente | GBIF | LC | 1 |
| Birds | Hemicircus_canente | Overall | LC | 2 |
| Birds | Hemipus_picatus | Facebook | LC | 96 |
| Birds | Hemipus_picatus | GBIF | LC | 77 |
| Birds | Hemipus_picatus | Overall | LC | 173 |
| Birds | Hemixos_flavala | Facebook | LC | 18 |
| Birds | Hemixos_flavala | GBIF | LC | 15 |
| Birds | Hemixos_flavala | Overall | LC | 33 |
| Birds | Hieraaetus_pennatus | Facebook | LC | 85 |
| Birds | Hieraaetus_pennatus | GBIF | LC | 110 |
| Birds | Hieraaetus_pennatus | Overall | LC | 195 |
| Birds | Hierococcyx_sparverioides | Facebook | LC | 15 |
| Birds | Hierococcyx_sparverioides | GBIF | LC | 7 |
| Birds | Hierococcyx_sparverioides | Overall | LC | 22 |
| Birds | Hierococcyx_varius | Facebook | LC | 92 |
| Birds | Hierococcyx_varius | GBIF | LC | 604 |
| Birds | Hierococcyx_varius | Overall | LC | 696 |
| Birds | Himantopus_himantopus | Facebook | LC | 95 |
| Birds | Himantopus_himantopus | GBIF | LC | 51 |
| Birds | Himantopus_himantopus | Overall | LC | 146 |
| Birds | Hirundapus_giganteus | Facebook | LC | 12 |
| Birds | Hirundapus_giganteus | GBIF | LC | 19 |
| Birds | Hirundapus_giganteus | Overall | LC | 31 |
| Birds | Hirundo_daurica | Facebook | LC | 41 |
| Birds | Hirundo_daurica | Overall | LC | 41 |
| Birds | Hirundo_rustica | Facebook | LC | 95 |
| Birds | Hirundo_rustica | GBIF | LC | 1522 |
| Birds | Hirundo_rustica | Overall | LC | 1617 |
| Birds | Hydrophasianus_chirurgus | Facebook | LC | 100 |
| Birds | Hydrophasianus_chirurgus | GBIF | LC | 152 |
| Birds | Hydrophasianus_chirurgus | Overall | LC | 252 |
| Birds | Hydroprogne_caspia | Facebook | LC | 6 |
| Birds | Hydroprogne_caspia | GBIF | LC | 2 |
| Birds | Hydroprogne_caspia | Overall | LC | 8 |
| Birds | Hypothymis_azurea | Facebook | LC | 97 |
| Birds | Hypothymis_azurea | GBIF | LC | 377 |
| Birds | Hypothymis_azurea | Overall | LC | 474 |
| Birds | Hypsipetes_leucocephalus | Facebook | LC | 22 |
| Birds | Hypsipetes_leucocephalus | GBIF | LC | 10 |
| Birds | Hypsipetes_leucocephalus | Overall | LC | 32 |
| Birds | Ichthyophaga_ichthyaetus | Facebook | NT | 288 |
| Birds | Ichthyophaga_ichthyaetus | Overall | NT | 288 |
| Birds | Ictinaetus_malayensis | Facebook | DD | 27 |
| Birds | Ictinaetus_malayensis | GBIF | DD | 8 |
| Birds | Ictinaetus_malayensis | Overall | DD | 35 |
| Birds | Irena_puella | Facebook | LC | 42 |
| Birds | Irena_puella | GBIF | LC | 42 |
| Birds | Irena_puella | Overall | LC | 84 |
| Birds | Ixobrychus_cinnamomeus | Facebook | LC | 86 |
| Birds | Ixobrychus_cinnamomeus | GBIF | LC | 142 |
| Birds | Ixobrychus_cinnamomeus | Overall | LC | 228 |
| Birds | Ixobrychus_flavicollis | Facebook | NT | 35 |
| Birds | Ixobrychus_flavicollis | GBIF | NT | 84 |
| Birds | Ixobrychus_flavicollis | Overall | NT | 119 |
| Birds | Ixobrychus_sinensis | Facebook | LC | 93 |
| Birds | Ixobrychus_sinensis | GBIF | LC | 331 |
| Birds | Ixobrychus_sinensis | Overall | LC | 424 |
| Birds | Jynx_torquilla | Facebook | LC | 176 |
| Birds | Jynx_torquilla | GBIF | LC | 189 |
| Birds | Jynx_torquilla | Overall | LC | 365 |
| Birds | Ketupa_ketupu | Facebook | DD | 29 |
| Birds | Ketupa_ketupu | GBIF | DD | 3 |
| Birds | Ketupa_ketupu | Overall | DD | 32 |
| Birds | Ketupa_zeylonensis | Facebook | LC | 95 |
| Birds | Ketupa_zeylonensis | GBIF | LC | 71 |
| Birds | Ketupa_zeylonensis | Overall | LC | 166 |
| Birds | Lanius_collurioides | Facebook | LC | 28 |
| Birds | Lanius_collurioides | GBIF | LC | 9 |
| Birds | Lanius_collurioides | Overall | LC | 37 |
| Birds | Lanius_cristatus | Facebook | LC | 370 |
| Birds | Lanius_cristatus | GBIF | LC | 986 |
| Birds | Lanius_cristatus | Overall | LC | 1356 |
| Birds | Lanius_schach | Facebook | LC | 430 |
| Birds | Lanius_schach | GBIF | LC | 1202 |
| Birds | Lanius_schach | Overall | LC | 1632 |
| Birds | Lanius_tephronotus | Facebook | LC | 140 |
| Birds | Lanius_tephronotus | GBIF | LC | 160 |
| Birds | Lanius_tephronotus | Overall | LC | 300 |
| Birds | Larus_brunnicephalus | Facebook | LC | 93 |
| Birds | Larus_brunnicephalus | Overall | LC | 93 |
| Birds | Larus_fuscus | Facebook | LC | 20 |
| Birds | Larus_fuscus | GBIF | LC | 30 |
| Birds | Larus_fuscus | Overall | LC | 50 |
| Birds | Larus_ichthyaetus | Facebook | LC | 101 |
| Birds | Larus_ichthyaetus | Overall | LC | 101 |
| Birds | Larus_ridibundus | Facebook | LC | 69 |
| Birds | Larus_ridibundus | GBIF | LC | 1 |
| Birds | Larus_ridibundus | Overall | LC | 70 |
| Birds | Leiopicus_mahrattensis | Facebook | LC | 1 |
| Birds | Leiopicus_mahrattensis | Overall | LC | 1 |
| Birds | Leptoptilos_javanicus | Facebook | VU | 59 |
| Birds | Leptoptilos_javanicus | GBIF | VU | 41 |
| Birds | Leptoptilos_javanicus | Overall | VU | 100 |
| Birds | Lewinia_striatus | Facebook | LC | 56 |
| Birds | Lewinia_striatus | Overall | LC | 56 |
| Birds | Limnodromus_semipalmatus | Facebook | EN | 3 |
| Birds | Limnodromus_semipalmatus | Overall | EN | 3 |
| Birds | Limosa_lapponica | Facebook | NT | 8 |
| Birds | Limosa_lapponica | GBIF | NT | 15 |
| Birds | Limosa_lapponica | Overall | NT | 23 |
| Birds | Limosa_limosa | Facebook | NT | 70 |
| Birds | Limosa_limosa | GBIF | NT | 63 |
| Birds | Limosa_limosa | Overall | NT | 133 |
| Birds | Locustella_certhiola | Facebook | LC | 6 |
| Birds | Locustella_certhiola | GBIF | LC | 18 |
| Birds | Locustella_certhiola | Overall | LC | 24 |
| Birds | Locustella_lanceolata | Facebook | LC | 1 |
| Birds | Locustella_lanceolata | GBIF | LC | 3 |
| Birds | Locustella_lanceolata | Overall | LC | 4 |
| Birds | Lonchura_atricapilla | Facebook | LC | 88 |
| Birds | Lonchura_atricapilla | GBIF | LC | 349 |
| Birds | Lonchura_atricapilla | Overall | LC | 437 |
| Birds | Lonchura_malabarica | Facebook | LC | 106 |
| Birds | Lonchura_malabarica | Overall | LC | 106 |
| Birds | Lonchura_malacca | Facebook | LC | 90 |
| Birds | Lonchura_malacca | GBIF | LC | 182 |
| Birds | Lonchura_malacca | Overall | LC | 272 |
| Birds | Lonchura_punctulata | Facebook | LC | 94 |
| Birds | Lonchura_punctulata | GBIF | LC | 560 |
| Birds | Lonchura_punctulata | Overall | LC | 654 |
| Birds | Lonchura_striata | Facebook | LC | 85 |
| Birds | Lonchura_striata | GBIF | LC | 184 |
| Birds | Lonchura_striata | Overall | LC | 269 |
| Birds | Lophotriorchis_kienerii | Facebook | VU | 10 |
| Birds | Lophotriorchis_kienerii | GBIF | VU | 5 |
| Birds | Lophotriorchis_kienerii | Overall | VU | 15 |
| Birds | Lophura_leucumelanos | Facebook | VU | 17 |
| Birds | Lophura_leucumelanos | Overall | VU | 17 |
| Birds | Loriculus_vernalis | Facebook | LC | 183 |
| Birds | Loriculus_vernalis | GBIF | LC | 87 |
| Birds | Loriculus_vernalis | Overall | LC | 270 |
| Birds | Luscinia_brunnea | Facebook | LC | 11 |
| Birds | Luscinia_brunnea | GBIF | LC | 10 |
| Birds | Luscinia_brunnea | Overall | LC | 21 |
| Birds | Luscinia_calliope | Facebook | LC | 83 |
| Birds | Luscinia_calliope | GBIF | LC | 94 |
| Birds | Luscinia_calliope | Overall | LC | 177 |
| Birds | Luscinia_cyane | Facebook | LC | 14 |
| Birds | Luscinia_cyane | GBIF | LC | 15 |
| Birds | Luscinia_cyane | Overall | LC | 29 |
| Birds | Luscinia_pectardens | Facebook | NT | 5 |
| Birds | Luscinia_pectardens | GBIF | NT | 1 |
| Birds | Luscinia_pectardens | Overall | NT | 6 |
| Birds | Luscinia_pectoralis | Facebook | NT | 2 |
| Birds | Luscinia_pectoralis | Overall | NT | 2 |
| Birds | Luscinia_svecica | Facebook | LC | 104 |
| Birds | Luscinia_svecica | GBIF | LC | 103 |
| Birds | Luscinia_svecica | Overall | LC | 207 |
| Birds | Lymnocryptes_minimus | Facebook | DD | 2 |
| Birds | Lymnocryptes_minimus | GBIF | DD | 1 |
| Birds | Lymnocryptes_minimus | Overall | DD | 3 |
| Birds | Lyncornis_macrotis | GBIF | NT | 3 |
| Birds | Lyncornis_macrotis | Overall | NT | 3 |
| Birds | Macronous_gularis | Facebook | LC | 58 |
| Birds | Macronous_gularis | Overall | LC | 58 |
| Birds | Malacocincla_abbotti | Facebook | LC | 85 |
| Birds | Malacocincla_abbotti | GBIF | LC | 11 |
| Birds | Malacocincla_abbotti | Overall | LC | 96 |
| Birds | Mareca_falcata | Facebook | NT | 13 |
| Birds | Mareca_falcata | GBIF | NT | 30 |
| Birds | Mareca_falcata | Overall | NT | 43 |
| Birds | Mareca_penelope | Facebook | LC | 53 |
| Birds | Mareca_penelope | GBIF | LC | 60 |
| Birds | Mareca_penelope | Overall | LC | 113 |
| Birds | Mareca_strepera | Facebook | LC | 125 |
| Birds | Mareca_strepera | GBIF | LC | 150 |
| Birds | Mareca_strepera | Overall | LC | 275 |
| Birds | Megaceryle_lugubris | Facebook | DD | 3 |
| Birds | Megaceryle_lugubris | Overall | DD | 3 |
| Birds | Megalurus_palustris | Facebook | LC | 92 |
| Birds | Megalurus_palustris | GBIF | LC | 469 |
| Birds | Megalurus_palustris | Overall | LC | 561 |
| Birds | Merops_leschenaulti | Facebook | LC | 192 |
| Birds | Merops_leschenaulti | GBIF | LC | 214 |
| Birds | Merops_leschenaulti | Overall | LC | 406 |
| Birds | Merops_orientalis | Facebook | LC | 540 |
| Birds | Merops_orientalis | GBIF | LC | 987 |
| Birds | Merops_orientalis | Overall | LC | 1527 |
| Birds | Merops_philippinus | Facebook | LC | 254 |
| Birds | Merops_philippinus | GBIF | LC | 196 |
| Birds | Merops_philippinus | Overall | LC | 450 |
| Birds | Metopidius_indicus | Facebook | LC | 90 |
| Birds | Metopidius_indicus | GBIF | LC | 605 |
| Birds | Metopidius_indicus | Overall | LC | 695 |
| Birds | Microcarbo_niger | Facebook | LC | 87 |
| Birds | Microcarbo_niger | GBIF | LC | 2896 |
| Birds | Microcarbo_niger | Overall | LC | 2983 |
| Birds | Milvus_migrans | Facebook | LC | 69 |
| Birds | Milvus_migrans | GBIF | LC | 5130 |
| Birds | Milvus_migrans | Overall | LC | 5199 |
| Birds | Mirafra_assamica | Facebook | LC | 90 |
| Birds | Mirafra_assamica | GBIF | LC | 323 |
| Birds | Mirafra_assamica | Overall | LC | 413 |
| Birds | Monticola_solitarius | Facebook | LC | 75 |
| Birds | Monticola_solitarius | GBIF | LC | 42 |
| Birds | Monticola_solitarius | Overall | LC | 117 |
| Birds | Motaciila_flava | Facebook | LC | 79 |
| Birds | Motaciila_flava | Overall | LC | 79 |
| Birds | Motacilla_alba | Facebook | LC | 298 |
| Birds | Motacilla_alba | GBIF | LC | 1345 |
| Birds | Motacilla_alba | Overall | LC | 1643 |
| Birds | Motacilla_cinerea | Facebook | LC | 86 |
| Birds | Motacilla_cinerea | GBIF | LC | 210 |
| Birds | Motacilla_cinerea | Overall | LC | 296 |
| Birds | Motacilla_citreola | Facebook | LC | 192 |
| Birds | Motacilla_citreola | GBIF | LC | 517 |
| Birds | Motacilla_citreola | Overall | LC | 709 |
| Birds | Motacilla_madaraspatensis | Facebook | LC | 166 |
| Birds | Motacilla_madaraspatensis | Overall | LC | 166 |
| Birds | Mulleripicus_pulverulentus | Facebook | NT | 20 |
| Birds | Mulleripicus_pulverulentus | GBIF | NT | 11 |
| Birds | Mulleripicus_pulverulentus | Overall | NT | 31 |
| Birds | Muscicapa_dauurica | Facebook | LC | 36 |
| Birds | Muscicapa_dauurica | GBIF | LC | 22 |
| Birds | Muscicapa_dauurica | Overall | LC | 58 |
| Birds | Muscicapa_muttui | Facebook | LC | 49 |
| Birds | Muscicapa_muttui | GBIF | LC | 20 |
| Birds | Muscicapa_muttui | Overall | LC | 69 |
| Birds | Muscicapa_sibirica | Facebook | LC | 35 |
| Birds | Muscicapa_sibirica | GBIF | LC | 15 |
| Birds | Muscicapa_sibirica | Overall | LC | 50 |
| Birds | Mycteria_leucocephala | Facebook | CR | 62 |
| Birds | Mycteria_leucocephala | GBIF | CR | 59 |
| Birds | Mycteria_leucocephala | Overall | CR | 121 |
| Birds | Myophonus_caeruleus | Facebook | LC | 34 |
| Birds | Myophonus_caeruleus | GBIF | LC | 18 |
| Birds | Myophonus_caeruleus | Overall | LC | 52 |
| Birds | Nectarinia_sperata | Facebook | LC | 32 |
| Birds | Nectarinia_sperata | GBIF | LC | 9 |
| Birds | Nectarinia_sperata | Overall | LC | 41 |
| Birds | Nectarinia_zeylonica | Facebook | LC | 91 |
| Birds | Nectarinia_zeylonica | Overall | LC | 91 |
| Birds | Neophron_percnopterus | Facebook | DD | 1 |
| Birds | Neophron_percnopterus | GBIF | DD | 1 |
| Birds | Neophron_percnopterus | Overall | DD | 2 |
| Birds | Netta_rufina | Facebook | LC | 97 |
| Birds | Netta_rufina | GBIF | LC | 51 |
| Birds | Netta_rufina | Overall | LC | 148 |
| Birds | Nettapus_coromandelianus | Facebook | LC | 99 |
| Birds | Nettapus_coromandelianus | GBIF | LC | 65 |
| Birds | Nettapus_coromandelianus | Overall | LC | 164 |
| Birds | Niltava_macgrigoriae | Facebook | LC | 8 |
| Birds | Niltava_macgrigoriae | GBIF | LC | 4 |
| Birds | Niltava_macgrigoriae | Overall | LC | 12 |
| Birds | Niltava_sundara | Facebook | LC | 8 |
| Birds | Niltava_sundara | GBIF | LC | 6 |
| Birds | Niltava_sundara | Overall | LC | 14 |
| Birds | Ninox_scutulata | Facebook | LC | 120 |
| Birds | Ninox_scutulata | GBIF | LC | 173 |
| Birds | Ninox_scutulata | Overall | LC | 293 |
| Birds | Nisaetus_cirrhatus | Facebook | LC | 96 |
| Birds | Nisaetus_cirrhatus | GBIF | LC | 64 |
| Birds | Nisaetus_cirrhatus | Overall | LC | 160 |
| Birds | Nisaetus_nipalensis | Facebook | VU | 7 |
| Birds | Nisaetus_nipalensis | GBIF | VU | 7 |
| Birds | Nisaetus_nipalensis | Overall | VU | 14 |
| Birds | Numenius_arquata | Facebook | NT | 98 |
| Birds | Numenius_arquata | GBIF | NT | 60 |
| Birds | Numenius_arquata | Overall | NT | 158 |
| Birds | Numenius_phaeopus | Facebook | LC | 83 |
| Birds | Numenius_phaeopus | GBIF | LC | 44 |
| Birds | Numenius_phaeopus | Overall | LC | 127 |
| Birds | Nycticorax_nycticorax | Facebook | LC | 95 |
| Birds | Nycticorax_nycticorax | GBIF | LC | 753 |
| Birds | Nycticorax_nycticorax | Overall | LC | 848 |
| Birds | Nyctyornis_athertoni | Facebook | LC | 150 |
| Birds | Nyctyornis_athertoni | GBIF | LC | 72 |
| Birds | Nyctyornis_athertoni | Overall | LC | 222 |
| Birds | Oriolus_chinensis | Facebook | LC | 47 |
| Birds | Oriolus_chinensis | GBIF | LC | 93 |
| Birds | Oriolus_chinensis | Overall | LC | 140 |
| Birds | Oriolus_oriolus | Facebook | LC | 37 |
| Birds | Oriolus_oriolus | Overall | LC | 37 |
| Birds | Oriolus_traillii | Facebook | LC | 14 |
| Birds | Oriolus_traillii | GBIF | LC | 10 |
| Birds | Oriolus_traillii | Overall | LC | 24 |
| Birds | Oriolus_xanthornus | Facebook | LC | 99 |
| Birds | Oriolus_xanthornus | GBIF | LC | 2222 |
| Birds | Oriolus_xanthornus | Overall | LC | 2321 |
| Birds | Orthotomus_atrogularis | Facebook | LC | 18 |
| Birds | Orthotomus_atrogularis | GBIF | LC | 45 |
| Birds | Orthotomus_atrogularis | Overall | LC | 63 |
| Birds | Orthotomus_cuculatus | Facebook | LC | 1 |
| Birds | Orthotomus_cuculatus | Overall | LC | 1 |
| Birds | Orthotomus_sutorius | Facebook | LC | 66 |
| Birds | Orthotomus_sutorius | GBIF | LC | 3678 |
| Birds | Orthotomus_sutorius | Overall | LC | 3744 |
| Birds | Otus_lettia | Facebook | LC | 89 |
| Birds | Otus_lettia | GBIF | LC | 139 |
| Birds | Otus_lettia | Overall | LC | 228 |
| Birds | Otus_spilocephalus | GBIF | DD | 5 |
| Birds | Otus_spilocephalus | Overall | DD | 5 |
| Birds | Otus_sunia | Facebook | LC | 35 |
| Birds | Otus_sunia | GBIF | LC | 30 |
| Birds | Otus_sunia | Overall | LC | 65 |
| Birds | Pandion_haliaetus | Facebook | LC | 98 |
| Birds | Pandion_haliaetus | GBIF | LC | 145 |
| Birds | Pandion_haliaetus | Overall | LC | 243 |
| Birds | Parus_major | Facebook | LC | 84 |
| Birds | Parus_major | GBIF | LC | 1 |
| Birds | Parus_major | Overall | LC | 85 |
| Birds | Passer_domesticus | Facebook | LC | 85 |
| Birds | Passer_domesticus | GBIF | LC | 5224 |
| Birds | Passer_domesticus | Overall | LC | 5309 |
| Birds | Passer_montanus | Facebook | LC | 29 |
| Birds | Passer_montanus | GBIF | LC | 32 |
| Birds | Passer_montanus | Overall | LC | 61 |
| Birds | Pelargopsis_amauroptera | Facebook | VU | 54 |
| Birds | Pelargopsis_amauroptera | GBIF | VU | 32 |
| Birds | Pelargopsis_amauroptera | Overall | VU | 86 |
| Birds | Pelargopsis_capensis | Facebook | LC | 299 |
| Birds | Pelargopsis_capensis | GBIF | LC | 615 |
| Birds | Pelargopsis_capensis | Overall | LC | 914 |
| Birds | Pelecanus_philippensis | Facebook | RE | 2 |
| Birds | Pelecanus_philippensis | Overall | RE | 2 |
| Birds | Pellorneum_albiventre | Facebook | DD | 2 |
| Birds | Pellorneum_albiventre | GBIF | DD | 1 |
| Birds | Pellorneum_albiventre | Overall | DD | 3 |
| Birds | Pellorneum_palustre | Facebook | LC | 1 |
| Birds | Pellorneum_palustre | Overall | LC | 1 |
| Birds | Pellorneum_ruficeps | Facebook | LC | 81 |
| Birds | Pellorneum_ruficeps | GBIF | LC | 198 |
| Birds | Pellorneum_ruficeps | Overall | LC | 279 |
| Birds | Pericrocotus_cantonensis | Facebook | LC | 41 |
| Birds | Pericrocotus_cantonensis | GBIF | LC | 41 |
| Birds | Pericrocotus_cantonensis | Overall | LC | 82 |
| Birds | Pericrocotus_cinnamomeus | Facebook | LC | 264 |
| Birds | Pericrocotus_cinnamomeus | GBIF | LC | 367 |
| Birds | Pericrocotus_cinnamomeus | Overall | LC | 631 |
| Birds | Pericrocotus_divaricatus | Facebook | LC | 28 |
| Birds | Pericrocotus_divaricatus | GBIF | LC | 16 |
| Birds | Pericrocotus_divaricatus | Overall | LC | 44 |
| Birds | Pericrocotus_flammeus | Facebook | LC | 93 |
| Birds | Pericrocotus_flammeus | GBIF | LC | 3 |
| Birds | Pericrocotus_flammeus | Overall | LC | 96 |
| Birds | Pericrocotus_roseus | Facebook | LC | 54 |
| Birds | Pericrocotus_roseus | GBIF | LC | 55 |
| Birds | Pericrocotus_roseus | Overall | LC | 109 |
| Birds | Pernis_ptilorhyncus | Facebook | LC | 100 |
| Birds | Pernis_ptilorhyncus | Overall | LC | 100 |
| Birds | Phaenicophaeus_tristis | Facebook | LC | 100 |
| Birds | Phaenicophaeus_tristis | GBIF | LC | 212 |
| Birds | Phaenicophaeus_tristis | Overall | LC | 312 |
| Birds | Phalacrocorax_carbo | Facebook | LC | 100 |
| Birds | Phalacrocorax_carbo | GBIF | LC | 280 |
| Birds | Phalacrocorax_carbo | Overall | LC | 380 |
| Birds | Phalacrocorax_fuscicollis | Facebook | LC | 60 |
| Birds | Phalacrocorax_fuscicollis | GBIF | LC | 91 |
| Birds | Phalacrocorax_fuscicollis | Overall | LC | 151 |
| Birds | Phoenicurus_ochruros | Facebook | LC | 54 |
| Birds | Phoenicurus_ochruros | GBIF | LC | 42 |
| Birds | Phoenicurus_ochruros | Overall | LC | 96 |
| Birds | Phylloscopus_affinis | Facebook | LC | 35 |
| Birds | Phylloscopus_affinis | GBIF | LC | 61 |
| Birds | Phylloscopus_affinis | Overall | LC | 96 |
| Birds | Phylloscopus_cantator | Facebook | LC | 7 |
| Birds | Phylloscopus_cantator | GBIF | LC | 11 |
| Birds | Phylloscopus_cantator | Overall | LC | 18 |
| Birds | Phylloscopus_collybita | Facebook | LC | 38 |
| Birds | Phylloscopus_collybita | GBIF | LC | 47 |
| Birds | Phylloscopus_collybita | Overall | LC | 85 |
| Birds | Phylloscopus_fuscatus | Facebook | LC | 98 |
| Birds | Phylloscopus_fuscatus | GBIF | LC | 500 |
| Birds | Phylloscopus_fuscatus | Overall | LC | 598 |
| Birds | Phylloscopus_inornatus | Facebook | LC | 47 |
| Birds | Phylloscopus_inornatus | GBIF | LC | 329 |
| Birds | Phylloscopus_inornatus | Overall | LC | 376 |
| Birds | Phylloscopus_occipitalis | Facebook | DD | 5 |
| Birds | Phylloscopus_occipitalis | GBIF | DD | 1 |
| Birds | Phylloscopus_occipitalis | Overall | DD | 6 |
| Birds | Phylloscopus_reguloides | GBIF | LC | 53 |
| Birds | Phylloscopus_reguloides | Overall | LC | 53 |
| Birds | Phylloscopus_trochiloides | Facebook | LC | 77 |
| Birds | Phylloscopus_trochiloides | GBIF | LC | 272 |
| Birds | Phylloscopus_trochiloides | Overall | LC | 349 |
| Birds | Picumnus_innominatus | Facebook | LC | 43 |
| Birds | Picumnus_innominatus | GBIF | LC | 18 |
| Birds | Picumnus_innominatus | Overall | LC | 61 |
| Birds | Picus_chlorolophus | Facebook | LC | 44 |
| Birds | Picus_chlorolophus | GBIF | LC | 65 |
| Birds | Picus_chlorolophus | Overall | LC | 109 |
| Birds | Picus_flavinucha | Facebook | LC | 48 |
| Birds | Picus_flavinucha | Overall | LC | 48 |
| Birds | Picus_guerini | Facebook | LC | 39 |
| Birds | Picus_guerini | Overall | LC | 39 |
| Birds | Picus_viridanus | Facebook | LC | 15 |
| Birds | Picus_viridanus | GBIF | LC | 5 |
| Birds | Picus_viridanus | Overall | LC | 20 |
| Birds | Picus_xanthopygaeus | Facebook | LC | 248 |
| Birds | Picus_xanthopygaeus | GBIF | LC | 175 |
| Birds | Picus_xanthopygaeus | Overall | LC | 423 |
| Birds | Pitta_brachyura | Facebook | LC | 208 |
| Birds | Pitta_brachyura | GBIF | LC | 23 |
| Birds | Pitta_brachyura | Overall | LC | 231 |
| Birds | Pitta_megarhyncha | Facebook | LC | 31 |
| Birds | Pitta_megarhyncha | GBIF | LC | 14 |
| Birds | Pitta_megarhyncha | Overall | LC | 45 |
| Birds | Pitta_nipalensis | Facebook | LC | 9 |
| Birds | Pitta_nipalensis | GBIF | LC | 23 |
| Birds | Pitta_nipalensis | Overall | LC | 32 |
| Birds | Pitta_sordida | Facebook | LC | 29 |
| Birds | Pitta_sordida | GBIF | LC | 21 |
| Birds | Pitta_sordida | Overall | LC | 50 |
| Birds | Platalea_leucorodia_Linnaeus | Facebook | CR | 20 |
| Birds | Platalea_leucorodia_Linnaeus | GBIF | CR | 5 |
| Birds | Platalea_leucorodia_Linnaeus | Overall | CR | 25 |
| Birds | Plegadis_falcinellus | Facebook | LC | 95 |
| Birds | Plegadis_falcinellus | GBIF | LC | 35 |
| Birds | Plegadis_falcinellus | Overall | LC | 130 |
| Birds | Ploceus_benghalensis | Facebook | LC | 87 |
| Birds | Ploceus_benghalensis | GBIF | LC | 37 |
| Birds | Ploceus_benghalensis | Overall | LC | 124 |
| Birds | Ploceus_manyar | Facebook | LC | 6 |
| Birds | Ploceus_manyar | GBIF | LC | 1 |
| Birds | Ploceus_manyar | Overall | LC | 7 |
| Birds | Ploceus_philippinus | Facebook | LC | 84 |
| Birds | Ploceus_philippinus | GBIF | LC | 494 |
| Birds | Ploceus_philippinus | Overall | LC | 578 |
| Birds | Pluvialis_fulva | Facebook | LC | 94 |
| Birds | Pluvialis_fulva | GBIF | LC | 62 |
| Birds | Pluvialis_fulva | Overall | LC | 156 |
| Birds | Pluvialis_squatarola | Facebook | LC | 39 |
| Birds | Pluvialis_squatarola | GBIF | LC | 50 |
| Birds | Pluvialis_squatarola | Overall | LC | 89 |
| Birds | Podiceps_cristatus | Facebook | LC | 71 |
| Birds | Podiceps_cristatus | GBIF | LC | 62 |
| Birds | Podiceps_cristatus | Overall | LC | 133 |
| Birds | Polyplectron_bicalcaratum | Facebook | VU | 4 |
| Birds | Polyplectron_bicalcaratum | GBIF | VU | 26 |
| Birds | Polyplectron_bicalcaratum | Overall | VU | 30 |
| Birds | Pomatorhinus_hypoleucos | Facebook | LC | 5 |
| Birds | Pomatorhinus_hypoleucos | GBIF | LC | 6 |
| Birds | Pomatorhinus_hypoleucos | Overall | LC | 11 |
| Birds | Pomatorhinus_mcclellandi | Facebook | RE | 1 |
| Birds | Pomatorhinus_mcclellandi | Overall | RE | 1 |
| Birds | Pomatorhinus_schisticeps | Facebook | NT | 14 |
| Birds | Pomatorhinus_schisticeps | GBIF | NT | 28 |
| Birds | Pomatorhinus_schisticeps | Overall | NT | 42 |
| Birds | Porphyrio_porphyrio | Facebook | LC | 93 |
| Birds | Porphyrio_porphyrio | GBIF | LC | 83 |
| Birds | Porphyrio_porphyrio | Overall | LC | 176 |
| Birds | Prinia_flaviventris | Facebook | LC | 21 |
| Birds | Prinia_flaviventris | GBIF | LC | 2 |
| Birds | Prinia_flaviventris | Overall | LC | 23 |
| Birds | Prinia_gracilis | Facebook | LC | 85 |
| Birds | Prinia_gracilis | GBIF | LC | 167 |
| Birds | Prinia_gracilis | Overall | LC | 252 |
| Birds | Prinia_hodgsonii | Facebook | LC | 60 |
| Birds | Prinia_hodgsonii | GBIF | LC | 119 |
| Birds | Prinia_hodgsonii | Overall | LC | 179 |
| Birds | Prinia_inornata | Facebook | LC | 126 |
| Birds | Prinia_inornata | GBIF | LC | 563 |
| Birds | Prinia_inornata | Overall | LC | 689 |
| Birds | Prinia_rufescens | Facebook | LC | 6 |
| Birds | Prinia_rufescens | GBIF | LC | 14 |
| Birds | Prinia_rufescens | Overall | LC | 20 |
| Birds | Psarisomus_dalhousiae | Facebook | DD | 19 |
| Birds | Psarisomus_dalhousiae | GBIF | DD | 7 |
| Birds | Psarisomus_dalhousiae | Overall | DD | 26 |
| Birds | Psilopogon_asiaticus | Facebook | LC | 362 |
| Birds | Psilopogon_asiaticus | GBIF | LC | 944 |
| Birds | Psilopogon_asiaticus | Overall | LC | 1306 |
| Birds | Psilopogon_asiaticus_ | Facebook | LC | 1 |
| Birds | Psilopogon_asiaticus_ | GBIF | LC | 944 |
| Birds | Psilopogon_asiaticus_ | Overall | LC | 945 |
| Birds | Psilopogon_australis | Facebook | LC | 20 |
| Birds | Psilopogon_australis | Overall | LC | 20 |
| Birds | Psilopogon_haemacephalus | Facebook | LC | 314 |
| Birds | Psilopogon_haemacephalus | GBIF | LC | 1459 |
| Birds | Psilopogon_haemacephalus | Overall | LC | 1773 |
| Birds | Psilopogon_lineatus | Facebook | LC | 217 |
| Birds | Psilopogon_lineatus | GBIF | LC | 1102 |
| Birds | Psilopogon_lineatus | Overall | LC | 1319 |
| Birds | Psilopogon_virens | Facebook | NT | 14 |
| Birds | Psilopogon_virens | GBIF | NT | 13 |
| Birds | Psilopogon_virens | Overall | NT | 27 |
| Birds | Psittacula_alexandri | Facebook | LC | 213 |
| Birds | Psittacula_alexandri | GBIF | LC | 283 |
| Birds | Psittacula_alexandri | Overall | LC | 496 |
| Birds | Psittacula_cyanocephala | Facebook | LC | 86 |
| Birds | Psittacula_cyanocephala | GBIF | LC | 27 |
| Birds | Psittacula_cyanocephala | Overall | LC | 113 |
| Birds | Psittacula_eupatria | Facebook | LC | 107 |
| Birds | Psittacula_eupatria | GBIF | LC | 187 |
| Birds | Psittacula_eupatria | Overall | LC | 294 |
| Birds | Psittacula_finschii | GBIF | VU | 2 |
| Birds | Psittacula_finschii | Overall | VU | 2 |
| Birds | Psittacula_krameri | Facebook | LC | 97 |
| Birds | Psittacula_krameri | GBIF | LC | 3495 |
| Birds | Psittacula_krameri | Overall | LC | 3592 |
| Birds | Psittacula_roseata | Facebook | NT | 97 |
| Birds | Psittacula_roseata | GBIF | NT | 93 |
| Birds | Psittacula_roseata | Overall | NT | 190 |
| Birds | Pycnonotus_atriceps | Facebook | LC | 93 |
| Birds | Pycnonotus_atriceps | GBIF | LC | 90 |
| Birds | Pycnonotus_atriceps | Overall | LC | 183 |
| Birds | Pycnonotus_cafer | Facebook | LC | 87 |
| Birds | Pycnonotus_cafer | GBIF | LC | 5331 |
| Birds | Pycnonotus_cafer | Overall | LC | 5418 |
| Birds | Pycnonotus_jocosus | Facebook | LC | 92 |
| Birds | Pycnonotus_jocosus | GBIF | LC | 535 |
| Birds | Pycnonotus_jocosus | Overall | LC | 627 |
| Birds | Pycnonotus_melanicterus | Facebook | LC | 93 |
| Birds | Pycnonotus_melanicterus | Overall | LC | 93 |
| Birds | Rallina_eurizonoides | Facebook | LC | 2 |
| Birds | Rallina_eurizonoides | GBIF | LC | 1 |
| Birds | Rallina_eurizonoides | Overall | LC | 3 |
| Birds | Rallus_aquaticus | Facebook | LC | 20 |
| Birds | Rallus_aquaticus | Overall | LC | 20 |
| Birds | Recurvirostra_avosetta | Facebook | LC | 98 |
| Birds | Recurvirostra_avosetta | GBIF | LC | 57 |
| Birds | Recurvirostra_avosetta | Overall | LC | 155 |
| Birds | Rhipidura_albicollis | Facebook | LC | 84 |
| Birds | Rhipidura_albicollis | GBIF | LC | 368 |
| Birds | Rhipidura_albicollis | Overall | LC | 452 |
| Birds | Rhyacornis_fuliginosa | Facebook | LC | 6 |
| Birds | Rhyacornis_fuliginosa | GBIF | LC | 1 |
| Birds | Rhyacornis_fuliginosa | Overall | LC | 7 |
| Birds | Rhyticeros_undulatus | Facebook | DD | 3 |
| Birds | Rhyticeros_undulatus | Overall | DD | 3 |
| Birds | Riparia_paludicola | Facebook | LC | 25 |
| Birds | Riparia_paludicola | Overall | LC | 25 |
| Birds | Riparia_riparia | Facebook | LC | 41 |
| Birds | Riparia_riparia | GBIF | LC | 136 |
| Birds | Riparia_riparia | Overall | LC | 177 |
| Birds | Rostratula_benghalensis | Facebook | LC | 92 |
| Birds | Rostratula_benghalensis | GBIF | LC | 74 |
| Birds | Rostratula_benghalensis | Overall | LC | 166 |
| Birds | Rynchops_albicollis | Facebook | CR | 49 |
| Birds | Rynchops_albicollis | GBIF | CR | 4 |
| Birds | Rynchops_albicollis | Overall | CR | 53 |
| Birds | Sarkidiornis_melanotos | Facebook | NT | 6 |
| Birds | Sarkidiornis_melanotos | GBIF | NT | 13 |
| Birds | Sarkidiornis_melanotos | Overall | NT | 19 |
| Birds | Sasia_ochracea | Facebook | LC | 17 |
| Birds | Sasia_ochracea | GBIF | LC | 10 |
| Birds | Sasia_ochracea | Overall | LC | 27 |
| Birds | Saxicola_caprata | Facebook | LC | 54 |
| Birds | Saxicola_caprata | GBIF | LC | 52 |
| Birds | Saxicola_caprata | Overall | LC | 106 |
| Birds | Saxicola_ferreus | Facebook | LC | 8 |
| Birds | Saxicola_ferreus | GBIF | LC | 6 |
| Birds | Saxicola_ferreus | Overall | LC | 14 |
| Birds | Saxicola_insignis | GBIF | DD | 1 |
| Birds | Saxicola_insignis | Overall | DD | 1 |
| Birds | Saxicola_jerdoni | GBIF | DD | 3 |
| Birds | Saxicola_jerdoni | Overall | DD | 3 |
| Birds | Saxicola_leucurus | Facebook | LC | 92 |
| Birds | Saxicola_leucurus | GBIF | LC | 91 |
| Birds | Saxicola_leucurus | Overall | LC | 183 |
| Birds | Saxicola_torquatus | Facebook | LC | 74 |
| Birds | Saxicola_torquatus | Overall | LC | 74 |
| Birds | Seicercus_burkii | Facebook | DD | 32 |
| Birds | Seicercus_burkii | GBIF | DD | 74 |
| Birds | Seicercus_burkii | Overall | DD | 106 |
| Birds | Seicercus_tephrocephalus | Facebook | DD | 2 |
| Birds | Seicercus_tephrocephalus | Overall | DD | 2 |
| Birds | Seicercus_whistleri | Facebook | DD | 3 |
| Birds | Seicercus_whistleri | GBIF | DD | 11 |
| Birds | Seicercus_whistleri | Overall | DD | 14 |
| Birds | Serilophus_lunatus | Facebook | LC | 13 |
| Birds | Serilophus_lunatus | GBIF | LC | 5 |
| Birds | Serilophus_lunatus | Overall | LC | 18 |
| Birds | Sitta_castanea | Facebook | LC | 6 |
| Birds | Sitta_castanea | Overall | LC | 6 |
| Birds | Sitta_frontalis | Facebook | LC | 85 |
| Birds | Sitta_frontalis | GBIF | LC | 41 |
| Birds | Sitta_frontalis | Overall | LC | 126 |
| Birds | Spatula_clypeata | Facebook | LC | 48 |
| Birds | Spatula_clypeata | GBIF | LC | 101 |
| Birds | Spatula_clypeata | Overall | LC | 149 |
| Birds | Spatula_querquedula | Facebook | LC | 76 |
| Birds | Spatula_querquedula | GBIF | LC | 111 |
| Birds | Spatula_querquedula | Overall | LC | 187 |
| Birds | Spilopelia_chinensis | GBIF | LC | 3386 |
| Birds | Spilopelia_chinensis | Overall | LC | 3386 |
| Birds | Spilopelia_suratensis* | Facebook | LC | 87 |
| Birds | Spilopelia_suratensis* | Overall | LC | 87 |
| Birds | Spilornis_cheela | Facebook | LC | 93 |
| Birds | Spilornis_cheela | GBIF | LC | 198 |
| Birds | Spilornis_cheela | Overall | LC | 291 |
| Birds | Stachyris_nigriceps | Facebook | LC | 22 |
| Birds | Stachyris_nigriceps | GBIF | LC | 27 |
| Birds | Stachyris_nigriceps | Overall | LC | 49 |
| Birds | Stachyris_rufifrons | Facebook | NT | 5 |
| Birds | Stachyris_rufifrons | Overall | NT | 5 |
| Birds | Sterna_acuticauda_ | Facebook | CR | 6 |
| Birds | Sterna_acuticauda_ | GBIF | CR | 2 |
| Birds | Sterna_acuticauda_ | Overall | CR | 8 |
| Birds | Sterna_albifrons | Facebook | LC | 94 |
| Birds | Sterna_albifrons | Overall | LC | 94 |
| Birds | Sterna_aurantia | Facebook | NT | 25 |
| Birds | Sterna_aurantia | GBIF | NT | 31 |
| Birds | Sterna_aurantia | Overall | NT | 56 |
| Birds | Sterna_hirundo | Facebook | LC | 21 |
| Birds | Sterna_hirundo | GBIF | LC | 7 |
| Birds | Sterna_hirundo | Overall | LC | 28 |
| Birds | Streptopelia_decaocto | Facebook | LC | 86 |
| Birds | Streptopelia_decaocto | GBIF | LC | 610 |
| Birds | Streptopelia_decaocto | Overall | LC | 696 |
| Birds | Streptopelia_orientalis | Facebook | LC | 19 |
| Birds | Streptopelia_orientalis | GBIF | LC | 56 |
| Birds | Streptopelia_orientalis | Overall | LC | 75 |
| Birds | Streptopelia_tranquebarica | Facebook | LC | 64 |
| Birds | Streptopelia_tranquebarica | GBIF | LC | 495 |
| Birds | Streptopelia_tranquebarica | Overall | LC | 559 |
| Birds | Strix_leptogrammica | Facebook | LC | 3 |
| Birds | Strix_leptogrammica | GBIF | LC | 5 |
| Birds | Strix_leptogrammica | Overall | LC | 8 |
| Birds | Sturnus_contra | Facebook | LC | 87 |
| Birds | Sturnus_contra | GBIF | LC | 13 |
| Birds | Sturnus_contra | Overall | LC | 100 |
| Birds | Sturnus_malabaricus | Facebook | LC | 94 |
| Birds | Sturnus_malabaricus | GBIF | LC | 8 |
| Birds | Sturnus_malabaricus | Overall | LC | 102 |
| Birds | Sturnus_pagodarum | Facebook | LC | 129 |
| Birds | Sturnus_pagodarum | Overall | LC | 129 |
| Birds | Sturnus_roseus | Facebook | LC | 20 |
| Birds | Sturnus_roseus | Overall | LC | 20 |
| Birds | Sturnus_vulgaris | Facebook | LC | 11 |
| Birds | Sturnus_vulgaris | GBIF | LC | 1 |
| Birds | Sturnus_vulgaris | Overall | LC | 12 |
| Birds | Surniculus_lugubris | Facebook | LC | 28 |
| Birds | Surniculus_lugubris | GBIF | LC | 46 |
| Birds | Surniculus_lugubris | Overall | LC | 74 |
| Birds | Tachybaptus_ruficollis | Facebook | LC | 97 |
| Birds | Tachybaptus_ruficollis | GBIF | LC | 276 |
| Birds | Tachybaptus_ruficollis | Overall | LC | 373 |
| Birds | Tadorna_ferruginea | Facebook | LC | 95 |
| Birds | Tadorna_ferruginea | GBIF | LC | 166 |
| Birds | Tadorna_ferruginea | Overall | LC | 261 |
| Birds | Tadorna_tadorna | Facebook | LC | 62 |
| Birds | Tadorna_tadorna | GBIF | LC | 34 |
| Birds | Tadorna_tadorna | Overall | LC | 96 |
| Birds | Tephrodornis_gularis | Facebook | LC | 33 |
| Birds | Tephrodornis_gularis | Overall | LC | 33 |
| Birds | Tephrodornis_pondicerianus | Facebook | LC | 93 |
| Birds | Tephrodornis_pondicerianus | GBIF | LC | 90 |
| Birds | Tephrodornis_pondicerianus | Overall | LC | 183 |
| Birds | Terpsiphone_paradisi | Facebook | LC | 94 |
| Birds | Terpsiphone_paradisi | GBIF | LC | 80 |
| Birds | Terpsiphone_paradisi | Overall | LC | 174 |
| Birds | Tesia_cyaniventer | Facebook | LC | 4 |
| Birds | Tesia_cyaniventer | GBIF | LC | 4 |
| Birds | Tesia_cyaniventer | Overall | LC | 8 |
| Birds | Thalasseus_bengalensis | Facebook | LC | 2 |
| Birds | Thalasseus_bengalensis | Overall | LC | 2 |
| Birds | Thalasseus_bergii | Facebook | LC | 13 |
| Birds | Thalasseus_bergii | GBIF | LC | 11 |
| Birds | Thalasseus_bergii | Overall | LC | 24 |
| Birds | Threskiornis_melanocephalus | Facebook | VU | 99 |
| Birds | Threskiornis_melanocephalus | GBIF | VU | 97 |
| Birds | Threskiornis_melanocephalus | Overall | VU | 196 |
| Birds | Timalia_pileata | Facebook | LC | 25 |
| Birds | Timalia_pileata | GBIF | LC | 16 |
| Birds | Timalia_pileata | Overall | LC | 41 |
| Birds | Todiramphus_chloris | Facebook | LC | 173 |
| Birds | Todiramphus_chloris | GBIF | LC | 93 |
| Birds | Todiramphus_chloris | Overall | LC | 266 |
| Birds | Treron_apicauda | Facebook | LC | 12 |
| Birds | Treron_apicauda | GBIF | LC | 7 |
| Birds | Treron_apicauda | Overall | LC | 19 |
| Birds | Treron_bicinctus | Facebook | LC | 62 |
| Birds | Treron_bicinctus | GBIF | LC | 41 |
| Birds | Treron_bicinctus | Overall | LC | 103 |
| Birds | Treron_curvirostra | Facebook | LC | 43 |
| Birds | Treron_curvirostra | GBIF | LC | 24 |
| Birds | Treron_curvirostra | Overall | LC | 67 |
| Birds | Treron_phayrei | Facebook | LC | 56 |
| Birds | Treron_phayrei | GBIF | LC | 67 |
| Birds | Treron_phayrei | Overall | LC | 123 |
| Birds | Treron_phoenicopterus | Facebook | LC | 93 |
| Birds | Treron_phoenicopterus | GBIF | LC | 542 |
| Birds | Treron_phoenicopterus | Overall | LC | 635 |
| Birds | Treron_sphenurus | Facebook | LC | 29 |
| Birds | Treron_sphenurus | GBIF | LC | 9 |
| Birds | Treron_sphenurus | Overall | LC | 38 |
| Birds | Trichastoma_tickelli | Facebook | EN | 2 |
| Birds | Trichastoma_tickelli | GBIF | EN | 1 |
| Birds | Trichastoma_tickelli | Overall | EN | 3 |
| Birds | Tringa_erythropus | Facebook | LC | 63 |
| Birds | Tringa_erythropus | GBIF | LC | 56 |
| Birds | Tringa_erythropus | Overall | LC | 119 |
| Birds | Tringa_glareola | Facebook | LC | 92 |
| Birds | Tringa_glareola | GBIF | LC | 265 |
| Birds | Tringa_glareola | Overall | LC | 357 |
| Birds | Tringa_guttife | Facebook | CR | 11 |
| Birds | Tringa_guttife | Overall | CR | 11 |
| Birds | Tringa_nebularia | Facebook | LC | 95 |
| Birds | Tringa_nebularia | GBIF | LC | 154 |
| Birds | Tringa_nebularia | Overall | LC | 249 |
| Birds | Tringa_ochropus | Facebook | LC | 86 |
| Birds | Tringa_ochropus | GBIF | LC | 375 |
| Birds | Tringa_ochropus | Overall | LC | 461 |
| Birds | Tringa_stagnatilis | Facebook | LC | 37 |
| Birds | Tringa_stagnatilis | GBIF | LC | 58 |
| Birds | Tringa_stagnatilis | Overall | LC | 95 |
| Birds | Tringa_totanus | Facebook | LC | 88 |
| Birds | Tringa_totanus | GBIF | LC | 90 |
| Birds | Tringa_totanus | Overall | LC | 178 |
| Birds | Turdoides_earlei | Facebook | LC | 91 |
| Birds | Turdoides_earlei | GBIF | LC | 210 |
| Birds | Turdoides_earlei | Overall | LC | 301 |
| Birds | Turdoides_striata | Facebook | LC | 93 |
| Birds | Turdoides_striata | GBIF | LC | 1066 |
| Birds | Turdoides_striata | Overall | LC | 1159 |
| Birds | Turdus_dissimilis | Facebook | LC | 9 |
| Birds | Turdus_dissimilis | GBIF | LC | 5 |
| Birds | Turdus_dissimilis | Overall | LC | 14 |
| Birds | Turdus_obscurus | Facebook | LC | 3 |
| Birds | Turdus_obscurus | GBIF | LC | 6 |
| Birds | Turdus_obscurus | Overall | LC | 9 |
| Birds | Turdus_ruficollis | Facebook | LC | 3 |
| Birds | Turdus_ruficollis | Overall | LC | 3 |
| Birds | Turdus_unicolor | Facebook | LC | 38 |
| Birds | Turdus_unicolor | GBIF | LC | 33 |
| Birds | Turdus_unicolor | Overall | LC | 71 |
| Birds | Turnix_suscitator | Facebook | LC | 37 |
| Birds | Turnix_suscitator | GBIF | LC | 21 |
| Birds | Turnix_suscitator | Overall | LC | 58 |
| Birds | Turnix_suscitator_ | Facebook | LC | 1 |
| Birds | Turnix_suscitator_ | GBIF | LC | 21 |
| Birds | Turnix_suscitator_ | Overall | LC | 22 |
| Birds | Tyto_alba | Facebook | LC | 50 |
| Birds | Tyto_alba | GBIF | LC | 127 |
| Birds | Tyto_alba | Overall | LC | 177 |
| Birds | Upupa_epops | Facebook | LC | 304 |
| Birds | Upupa_epops | GBIF | LC | 350 |
| Birds | Upupa_epops | Overall | LC | 654 |
| Birds | Urosphena_squameiceps | Facebook | LC | 4 |
| Birds | Urosphena_squameiceps | GBIF | LC | 9 |
| Birds | Urosphena_squameiceps | Overall | LC | 13 |
| Birds | Vanellus_cinereus | Facebook | LC | 97 |
| Birds | Vanellus_cinereus | GBIF | LC | 393 |
| Birds | Vanellus_cinereus | Overall | LC | 490 |
| Birds | Vanellus_duvaucelii | Facebook | NT | 96 |
| Birds | Vanellus_duvaucelii | GBIF | NT | 69 |
| Birds | Vanellus_duvaucelii | Overall | NT | 165 |
| Birds | Vanellus_indicus | Facebook | LC | 98 |
| Birds | Vanellus_indicus | GBIF | LC | 440 |
| Birds | Vanellus_indicus | Overall | LC | 538 |
| Birds | Vanellus_malabaricus | Facebook | NT | 95 |
| Birds | Vanellus_malabaricus | GBIF | NT | 23 |
| Birds | Vanellus_malabaricus | Overall | NT | 118 |
| Birds | Vanellus_vanellus | Facebook | LC | 24 |
| Birds | Vanellus_vanellus | GBIF | LC | 9 |
| Birds | Vanellus_vanellus | Overall | LC | 33 |
| Birds | Xenus_cinereus | Facebook | LC | 27 |
| Birds | Xenus_cinereus | GBIF | LC | 26 |
| Birds | Xenus_cinereus | Overall | LC | 53 |
| Birds | Yuhina_castaniceps | Facebook | DD | 5 |
| Birds | Yuhina_castaniceps | GBIF | DD | 4 |
| Birds | Yuhina_castaniceps | Overall | DD | 9 |
| Birds | Zapornia_fusca | Facebook | LC | 74 |
| Birds | Zapornia_fusca | GBIF | LC | 35 |
| Birds | Zapornia_fusca | Overall | LC | 109 |
| Birds | Zapornia_pusilla | Facebook | LC | 57 |
| Birds | Zapornia_pusilla | GBIF | LC | 21 |
| Birds | Zapornia_pusilla | Overall | LC | 78 |
| Birds | Zoothera_citrina | Facebook | LC | 322 |
| Birds | Zoothera_citrina | Overall | LC | 322 |
| Birds | Zoothera_dauma | Facebook | LC | 51 |
| Birds | Zoothera_dauma | GBIF | LC | 19 |
| Birds | Zoothera_dauma | Overall | LC | 70 |
| Birds | Zoothera_marginata | Facebook | DD | 1 |
| Birds | Zoothera_marginata | GBIF | DD | 4 |
| Birds | Zoothera_marginata | Overall | DD | 5 |
| Birds | Zosterops_palpebrosus | Facebook | LC | 91 |
| Birds | Zosterops_palpebrosus | GBIF | LC | 326 |
| Birds | Zosterops_palpebrosus | Overall | LC | 417 |
| Butterflies | Abisara_echerius | Facebook | EN | 9 |
| Butterflies | Abisara_echerius | Overall | EN | 9 |
| Butterflies | Acraea_violae | Facebook | LC | 52 |
| Butterflies | Acraea_violae | GBIF | LC | 2 |
| Butterflies | Acraea_violae | Overall | LC | 54 |
| Butterflies | Acytolepis_puspa | Facebook | VU | 21 |
| Butterflies | Acytolepis_puspa | Overall | VU | 21 |
| Butterflies | Aeromachus_pygmaeus | Facebook | VU | 27 |
| Butterflies | Aeromachus_pygmaeus | Overall | VU | 27 |
| Butterflies | Aeromachus_stigmata | Facebook | DD | 3 |
| Butterflies | Aeromachus_stigmata | Overall | DD | 3 |
| Butterflies | Amblypodia_anita | Facebook | EN | 7 |
| Butterflies | Amblypodia_anita | Overall | EN | 7 |
| Butterflies | Anthene_emolus | Facebook | VU | 56 |
| Butterflies | Anthene_emolus | GBIF | VU | 8 |
| Butterflies | Anthene_emolus | Overall | VU | 64 |
| Butterflies | Anthene_lycaenina | Facebook | EN | 45 |
| Butterflies | Anthene_lycaenina | GBIF | EN | 2 |
| Butterflies | Anthene_lycaenina | Overall | EN | 47 |
| Butterflies | Appias_albina | Facebook | EN | 26 |
| Butterflies | Appias_albina | Overall | EN | 26 |
| Butterflies | Appias_indra | Facebook | VU | 23 |
| Butterflies | Appias_indra | Overall | VU | 23 |
| Butterflies | Appias_lalage | Facebook | EN | 2 |
| Butterflies | Appias_lalage | Overall | EN | 2 |
| Butterflies | Appias_libythea | Facebook | LC | 78 |
| Butterflies | Appias_libythea | GBIF | LC | 10 |
| Butterflies | Appias_libythea | Overall | LC | 88 |
| Butterflies | Appias_lyncida | Facebook | LC | 51 |
| Butterflies | Appias_lyncida | GBIF | LC | 4 |
| Butterflies | Appias_lyncida | Overall | LC | 55 |
| Butterflies | Arhopala_amantes | Facebook | VU | 22 |
| Butterflies | Arhopala_amantes | Overall | VU | 22 |
| Butterflies | Arhopala_bazaloides | Facebook | DD | 3 |
| Butterflies | Arhopala_bazaloides | Overall | DD | 3 |
| Butterflies | Arhopala_centaurus | Facebook | LC | 47 |
| Butterflies | Arhopala_centaurus | Overall | LC | 47 |
| Butterflies | Arhopala_eumolphus | Facebook | VU | 22 |
| Butterflies | Arhopala_eumolphus | Overall | VU | 22 |
| Butterflies | Arhopala_silhetensis | Facebook | LC | 1 |
| Butterflies | Arhopala_silhetensis | Overall | LC | 1 |
| Butterflies | Ariadne_ariadne | Facebook | LC | 50 |
| Butterflies | Ariadne_ariadne | Overall | LC | 50 |
| Butterflies | Ariadne_merione | Facebook | LC | 51 |
| Butterflies | Ariadne_merione | GBIF | LC | 2 |
| Butterflies | Ariadne_merione | Overall | LC | 53 |
| Butterflies | Astictopterus_jama | Facebook | LC | 18 |
| Butterflies | Astictopterus_jama | GBIF | LC | 4 |
| Butterflies | Astictopterus_jama | Overall | LC | 22 |
| Butterflies | Athyma_asura | Facebook | DD | 5 |
| Butterflies | Athyma_asura | Overall | DD | 5 |
| Butterflies | Athyma_kanwa | Facebook | DD | 3 |
| Butterflies | Athyma_kanwa | Overall | DD | 3 |
| Butterflies | Athyma_nefte | Facebook | VU | 49 |
| Butterflies | Athyma_nefte | GBIF | VU | 1 |
| Butterflies | Athyma_nefte | Overall | VU | 50 |
| Butterflies | Athyma_perius | Facebook | LC | 44 |
| Butterflies | Athyma_perius | GBIF | LC | 2 |
| Butterflies | Athyma_perius | Overall | LC | 46 |
| Butterflies | Athyma_ranga | Facebook | VU | 27 |
| Butterflies | Athyma_ranga | Overall | VU | 27 |
| Butterflies | Athyma_selenophora | Facebook | DD | 8 |
| Butterflies | Athyma_selenophora | Overall | DD | 8 |
| Butterflies | Atrophaneura_varuna | Facebook | EN | 17 |
| Butterflies | Atrophaneura_varuna | GBIF | EN | 1 |
| Butterflies | Atrophaneura_varuna | Overall | EN | 18 |
| Butterflies | Badamia_exclamationis | Facebook | VU | 34 |
| Butterflies | Badamia_exclamationis | Overall | VU | 34 |
| Butterflies | Baoris_chapmani | Facebook | VU | 6 |
| Butterflies | Baoris_chapmani | Overall | VU | 6 |
| Butterflies | Baoris_unicolor | Facebook | EN | 7 |
| Butterflies | Baoris_unicolor | Overall | EN | 7 |
| Butterflies | Belenois_aurota | Facebook | EN | 18 |
| Butterflies | Belenois_aurota | Overall | EN | 18 |
| Butterflies | Bibasis_amara | Facebook | EN | 13 |
| Butterflies | Bibasis_amara | GBIF | EN | 1 |
| Butterflies | Bibasis_amara | Overall | EN | 14 |
| Butterflies | Bibasis_jaina | Facebook | DD | 1 |
| Butterflies | Bibasis_jaina | Overall | DD | 1 |
| Butterflies | Borbo_cinnara | Facebook | LC | 23 |
| Butterflies | Borbo_cinnara | Overall | LC | 23 |
| Butterflies | Caleta_decidia | Facebook | LC | 45 |
| Butterflies | Caleta_decidia | GBIF | LC | 3 |
| Butterflies | Caleta_decidia | Overall | LC | 48 |
| Butterflies | Caleta_elna | Facebook | EN | 3 |
| Butterflies | Caleta_elna | Overall | EN | 3 |
| Butterflies | Caltoris_cormasa | Facebook | EN | 4 |
| Butterflies | Caltoris_cormasa | Overall | EN | 4 |
| Butterflies | Caltoris_kumara | Facebook | EN | 4 |
| Butterflies | Caltoris_kumara | Overall | EN | 4 |
| Butterflies | Castalius_rosimon | Facebook | LC | 77 |
| Butterflies | Castalius_rosimon | GBIF | LC | 21 |
| Butterflies | Castalius_rosimon | Overall | LC | 98 |
| Butterflies | Catapaecilma_major | Facebook | EN | 27 |
| Butterflies | Catapaecilma_major | Overall | EN | 27 |
| Butterflies | Catochrysops_strabo | Facebook | VU | 40 |
| Butterflies | Catochrysops_strabo | GBIF | VU | 5 |
| Butterflies | Catochrysops_strabo | Overall | VU | 45 |
| Butterflies | Catopsilia_pomona | Facebook | LC | 66 |
| Butterflies | Catopsilia_pomona | GBIF | LC | 11 |
| Butterflies | Catopsilia_pomona | Overall | LC | 77 |
| Butterflies | Catopsilia_pyranthe | Facebook | LC | 68 |
| Butterflies | Catopsilia_pyranthe | GBIF | LC | 4 |
| Butterflies | Catopsilia_pyranthe | Overall | LC | 72 |
| Butterflies | Celaenorrhinus_aurivittata | Facebook | EN | 4 |
| Butterflies | Celaenorrhinus_aurivittata | Overall | EN | 4 |
| Butterflies | Celatoxia_albidisca | Facebook | DD | 2 |
| Butterflies | Celatoxia_albidisca | Overall | DD | 2 |
| Butterflies | Cephrenes_acalle | Facebook | VU | 40 |
| Butterflies | Cephrenes_acalle | Overall | VU | 40 |
| Butterflies | Cepora_nadina | Facebook | EN | 3 |
| Butterflies | Cepora_nadina | GBIF | EN | 1 |
| Butterflies | Cepora_nadina | Overall | EN | 4 |
| Butterflies | Cepora_nerissa | Facebook | LC | 55 |
| Butterflies | Cepora_nerissa | Overall | LC | 55 |
| Butterflies | Cethosia_cyane | Facebook | LC | 58 |
| Butterflies | Cethosia_cyane | GBIF | LC | 3 |
| Butterflies | Cethosia_cyane | Overall | LC | 61 |
| Butterflies | Charaxes_aristogiton | Facebook | DD | 2 |
| Butterflies | Charaxes_aristogiton | Overall | DD | 2 |
| Butterflies | Charaxes_marmax | Facebook | DD | 1 |
| Butterflies | Charaxes_marmax | Overall | DD | 1 |
| Butterflies | Charaxes_solon | Facebook | VU | 45 |
| Butterflies | Charaxes_solon | GBIF | VU | 1 |
| Butterflies | Charaxes_solon | Overall | VU | 46 |
| Butterflies | Cheritra_freja | Facebook | VU | 32 |
| Butterflies | Cheritra_freja | GBIF | VU | 2 |
| Butterflies | Cheritra_freja | Overall | VU | 34 |
| Butterflies | Chilades_lajus | Facebook | LC | 79 |
| Butterflies | Chilades_lajus | Overall | LC | 79 |
| Butterflies | Chilades_pandava | Facebook | LC | 66 |
| Butterflies | Chilades_pandava | Overall | LC | 66 |
| Butterflies | Chilades_parrhasius | Facebook | EN | 3 |
| Butterflies | Chilades_parrhasius | Overall | EN | 3 |
| Butterflies | Chilasa_clytia | Facebook | LC | 59 |
| Butterflies | Chilasa_clytia | GBIF | LC | 6 |
| Butterflies | Chilasa_clytia | Overall | LC | 65 |
| Butterflies | Chliaria_othona | Facebook | VU | 37 |
| Butterflies | Chliaria_othona | Overall | VU | 37 |
| Butterflies | Choaspes_benjaminii | Facebook | EN | 8 |
| Butterflies | Choaspes_benjaminii | Overall | EN | 8 |
| Butterflies | Cigaritis_elima | Facebook | DD | 7 |
| Butterflies | Cigaritis_elima | Overall | DD | 7 |
| Butterflies | Cirrochroa_tyche | Facebook | EN | 40 |
| Butterflies | Cirrochroa_tyche | GBIF | EN | 2 |
| Butterflies | Cirrochroa_tyche | Overall | EN | 42 |
| Butterflies | Cupha_erymanthis | Facebook | LC | 29 |
| Butterflies | Cupha_erymanthis | Overall | LC | 29 |
| Butterflies | Cupitha_purreea | Facebook | EN | 20 |
| Butterflies | Cupitha_purreea | GBIF | EN | 1 |
| Butterflies | Cupitha_purreea | Overall | EN | 21 |
| Butterflies | Curetis_bulis | Facebook | VU | 1 |
| Butterflies | Curetis_bulis | Overall | VU | 1 |
| Butterflies | Curetis_dentata | Facebook | DD | 6 |
| Butterflies | Curetis_dentata | Overall | DD | 6 |
| Butterflies | Curetis_saronis | Facebook | EN | 9 |
| Butterflies | Curetis_saronis | Overall | EN | 9 |
| Butterflies | Curetis_thetis | Facebook | LC | 42 |
| Butterflies | Curetis_thetis | Overall | LC | 42 |
| Butterflies | Cyrestis_thyodamas | Facebook | EN | 23 |
| Butterflies | Cyrestis_thyodamas | Overall | EN | 23 |
| Butterflies | Dacalana_penicilligera | Facebook | EN | 10 |
| Butterflies | Dacalana_penicilligera | Overall | EN | 10 |
| Butterflies | Danaus_chrysippus | Facebook | LC | 107 |
| Butterflies | Danaus_chrysippus | GBIF | LC | 15 |
| Butterflies | Danaus_chrysippus | Overall | LC | 122 |
| Butterflies | Danaus_genutia | Facebook | LC | 57 |
| Butterflies | Danaus_genutia | GBIF | LC | 7 |
| Butterflies | Danaus_genutia | Overall | LC | 64 |
| Butterflies | Danaus_melanippus | Facebook | EN | 30 |
| Butterflies | Danaus_melanippus | GBIF | EN | 1 |
| Butterflies | Danaus_melanippus | Overall | EN | 31 |
| Butterflies | Delias_descombesi | Facebook | LC | 64 |
| Butterflies | Delias_descombesi | GBIF | LC | 3 |
| Butterflies | Delias_descombesi | Overall | LC | 67 |
| Butterflies | Delias_eucharis | Facebook | LC | 80 |
| Butterflies | Delias_eucharis | GBIF | LC | 5 |
| Butterflies | Delias_eucharis | Overall | LC | 85 |
| Butterflies | Delias_hyparete | Facebook | LC | 54 |
| Butterflies | Delias_hyparete | GBIF | LC | 3 |
| Butterflies | Delias_hyparete | Overall | LC | 57 |
| Butterflies | Delias_pasithoe | Facebook | LC | 48 |
| Butterflies | Delias_pasithoe | GBIF | LC | 2 |
| Butterflies | Delias_pasithoe | Overall | LC | 50 |
| Butterflies | Discolampa_ethion | Facebook | VU | 49 |
| Butterflies | Discolampa_ethion | GBIF | VU | 3 |
| Butterflies | Discolampa_ethion | Overall | VU | 52 |
| Butterflies | Discophora_sondaica | Facebook | LC | 39 |
| Butterflies | Discophora_sondaica | GBIF | LC | 2 |
| Butterflies | Discophora_sondaica | Overall | LC | 41 |
| Butterflies | Dophla_evelina | Facebook | EN | 9 |
| Butterflies | Dophla_evelina | Overall | EN | 9 |
| Butterflies | Elymnias_hypermnestra | Facebook | LC | 75 |
| Butterflies | Elymnias_hypermnestra | GBIF | LC | 11 |
| Butterflies | Elymnias_hypermnestra | Overall | LC | 86 |
| Butterflies | Elymnias_malelas | Facebook | EN | 33 |
| Butterflies | Elymnias_malelas | GBIF | EN | 1 |
| Butterflies | Elymnias_malelas | Overall | EN | 34 |
| Butterflies | Elymnias_nesaea | Facebook | EN | 12 |
| Butterflies | Elymnias_nesaea | Overall | EN | 12 |
| Butterflies | Erionota_torus | Facebook | EN | 17 |
| Butterflies | Erionota_torus | Overall | EN | 17 |
| Butterflies | Euchrysops_Cnejus | Facebook | LC | 58 |
| Butterflies | Euchrysops_Cnejus | Overall | LC | 58 |
| Butterflies | Euploea_algea | Facebook | EN | 33 |
| Butterflies | Euploea_algea | GBIF | EN | 1 |
| Butterflies | Euploea_algea | Overall | EN | 34 |
| Butterflies | Euploea_core | Facebook | LC | 60 |
| Butterflies | Euploea_core | GBIF | LC | 12 |
| Butterflies | Euploea_core | Overall | LC | 72 |
| Butterflies | Euploea_crameri_nicevillei | Facebook | CR | 9 |
| Butterflies | Euploea_crameri_nicevillei | Overall | CR | 9 |
| Butterflies | Euploea_klugii | Facebook | VU | 56 |
| Butterflies | Euploea_klugii | Overall | VU | 56 |
| Butterflies | Euploea_midamus | Facebook | EN | 10 |
| Butterflies | Euploea_midamus | Overall | EN | 10 |
| Butterflies | Euploea_mulciber | Facebook | VU | 40 |
| Butterflies | Euploea_mulciber | GBIF | VU | 3 |
| Butterflies | Euploea_mulciber | Overall | VU | 43 |
| Butterflies | Euploea_sylvester | Facebook | EN | 4 |
| Butterflies | Euploea_sylvester | Overall | EN | 4 |
| Butterflies | Eurema_andersoni | Facebook | LC | 28 |
| Butterflies | Eurema_andersoni | Overall | LC | 28 |
| Butterflies | Eurema_blanda | Facebook | LC | 32 |
| Butterflies | Eurema_blanda | GBIF | LC | 1 |
| Butterflies | Eurema_blanda | Overall | LC | 33 |
| Butterflies | Eurema_hecabe | Facebook | LC | 91 |
| Butterflies | Eurema_hecabe | GBIF | LC | 17 |
| Butterflies | Eurema_hecabe | Overall | LC | 108 |
| Butterflies | Euripus_nyctelius | Facebook | EN | 33 |
| Butterflies | Euripus_nyctelius | GBIF | EN | 1 |
| Butterflies | Euripus_nyctelius | Overall | EN | 34 |
| Butterflies | Euthalia_aconthea | Facebook | LC | 64 |
| Butterflies | Euthalia_aconthea | GBIF | LC | 15 |
| Butterflies | Euthalia_aconthea | Overall | LC | 79 |
| Butterflies | Euthalia_lubentina | Facebook | EN | 38 |
| Butterflies | Euthalia_lubentina | GBIF | EN | 1 |
| Butterflies | Euthalia_lubentina | Overall | EN | 39 |
| Butterflies | Euthalia_monina | Facebook | EN | 42 |
| Butterflies | Euthalia_monina | GBIF | EN | 3 |
| Butterflies | Euthalia_monina | Overall | EN | 45 |
| Butterflies | Euthalia_phemius | Facebook | EN | 9 |
| Butterflies | Euthalia_phemius | Overall | EN | 9 |
| Butterflies | Everes_lacturnus | Facebook | EN | 1 |
| Butterflies | Everes_lacturnus | Overall | EN | 1 |
| Butterflies | Freyeria_putli | Facebook | DD | 5 |
| Butterflies | Freyeria_putli | Overall | DD | 5 |
| Butterflies | Gandaca_harina | Facebook | EN | 40 |
| Butterflies | Gandaca_harina | Overall | EN | 40 |
| Butterflies | Gangara_thyrsis | Facebook | VU | 22 |
| Butterflies | Gangara_thyrsis | Overall | VU | 22 |
| Butterflies | Gerosis_bhagava | Facebook | VU | 23 |
| Butterflies | Gerosis_bhagava | Overall | VU | 23 |
| Butterflies | Gerosis_phisara | Facebook | EN | 1 |
| Butterflies | Gerosis_phisara | Overall | EN | 1 |
| Butterflies | Graphium_agamemnon | Facebook | LC | 53 |
| Butterflies | Graphium_agamemnon | GBIF | LC | 3 |
| Butterflies | Graphium_agamemnon | Overall | LC | 56 |
| Butterflies | Graphium_doson | Facebook | LC | 58 |
| Butterflies | Graphium_doson | GBIF | LC | 8 |
| Butterflies | Graphium_doson | Overall | LC | 66 |
| Butterflies | Graphium_nomius | Facebook | EN | 36 |
| Butterflies | Graphium_nomius | GBIF | EN | 1 |
| Butterflies | Graphium_nomius | Overall | EN | 37 |
| Butterflies | Graphium_sarpedon | Facebook | VU | 36 |
| Butterflies | Graphium_sarpedon | Overall | VU | 36 |
| Butterflies | Graphium_xenocles | Facebook | EN | 16 |
| Butterflies | Graphium_xenocles | Overall | EN | 16 |
| Butterflies | Halpe_porus | Facebook | VU | 45 |
| Butterflies | Halpe_porus | Overall | VU | 45 |
| Butterflies | Hasora_badra | Facebook | VU | 43 |
| Butterflies | Hasora_badra | Overall | VU | 43 |
| Butterflies | Hasora_chromus | Facebook | EN | 20 |
| Butterflies | Hasora_chromus | Overall | EN | 20 |
| Butterflies | Hebomoia_glaucippe | Facebook | VU | 45 |
| Butterflies | Hebomoia_glaucippe | Overall | VU | 45 |
| Butterflies | Heliophorus_epicles | Facebook | VU | 16 |
| Butterflies | Heliophorus_epicles | Overall | VU | 16 |
| Butterflies | Hyarotis_adrastus | Facebook | VU | 49 |
| Butterflies | Hyarotis_adrastus | GBIF | VU | 2 |
| Butterflies | Hyarotis_adrastus | Overall | VU | 51 |
| Butterflies | Hypolimnas_bolina | Facebook | LC | 76 |
| Butterflies | Hypolimnas_bolina | GBIF | LC | 15 |
| Butterflies | Hypolimnas_bolina | Overall | LC | 91 |
| Butterflies | Hypolimnas_misippus | Facebook | VU | 39 |
| Butterflies | Hypolimnas_misippus | Overall | VU | 39 |
| Butterflies | Hypolycaena_erylus | Facebook | VU | 46 |
| Butterflies | Hypolycaena_erylus | GBIF | VU | 1 |
| Butterflies | Hypolycaena_erylus | Overall | VU | 47 |
| Butterflies | Iambrix_salsala | Facebook | LC | 66 |
| Butterflies | Iambrix_salsala | GBIF | LC | 7 |
| Butterflies | Iambrix_salsala | Overall | LC | 73 |
| Butterflies | Idea_agamarschana | GBIF | VU | 2 |
| Butterflies | Idea_agamarschana | Overall | VU | 2 |
| Butterflies | Ionolyce_helicon | Facebook | DD | 7 |
| Butterflies | Ionolyce_helicon | Overall | DD | 7 |
| Butterflies | Iraota_timoleon | Facebook | EN | 8 |
| Butterflies | Iraota_timoleon | Overall | EN | 8 |
| Butterflies | Iton_semamora | Facebook | EN | 28 |
| Butterflies | Iton_semamora | GBIF | EN | 2 |
| Butterflies | Iton_semamora | Overall | EN | 30 |
| Butterflies | Ixias_pyrene | Facebook | EN | 25 |
| Butterflies | Ixias_pyrene | Overall | EN | 25 |
| Butterflies | Jamides_alecto | Facebook | LC | 56 |
| Butterflies | Jamides_alecto | GBIF | LC | 7 |
| Butterflies | Jamides_alecto | Overall | LC | 63 |
| Butterflies | Jamides_bochus | Facebook | VU | 42 |
| Butterflies | Jamides_bochus | Overall | VU | 42 |
| Butterflies | Jamides_celeno | Facebook | LC | 54 |
| Butterflies | Jamides_celeno | GBIF | LC | 1 |
| Butterflies | Jamides_celeno | Overall | LC | 55 |
| Butterflies | Jamides_pura | Facebook | EN | 16 |
| Butterflies | Jamides_pura | Overall | EN | 16 |
| Butterflies | Junonia_almana | Facebook | LC | 93 |
| Butterflies | Junonia_almana | GBIF | LC | 24 |
| Butterflies | Junonia_almana | Overall | LC | 117 |
| Butterflies | Junonia_atlites | Facebook | LC | 85 |
| Butterflies | Junonia_atlites | GBIF | LC | 29 |
| Butterflies | Junonia_atlites | Overall | LC | 114 |
| Butterflies | Junonia_hierta | Facebook | LC | 46 |
| Butterflies | Junonia_hierta | GBIF | LC | 8 |
| Butterflies | Junonia_hierta | Overall | LC | 54 |
| Butterflies | Junonia_iphita | Facebook | LC | 52 |
| Butterflies | Junonia_iphita | GBIF | LC | 7 |
| Butterflies | Junonia_iphita | Overall | LC | 59 |
| Butterflies | Junonia_lemonias | Facebook | LC | 60 |
| Butterflies | Junonia_lemonias | GBIF | LC | 17 |
| Butterflies | Junonia_lemonias | Overall | LC | 77 |
| Butterflies | Junonia_orithya | Facebook | VU | 41 |
| Butterflies | Junonia_orithya | GBIF | VU | 3 |
| Butterflies | Junonia_orithya | Overall | VU | 44 |
| Butterflies | Kallima_inachus | Facebook | EN | 13 |
| Butterflies | Kallima_inachus | Overall | EN | 13 |
| Butterflies | Koruthaialos_rubecula | Facebook | EN | 15 |
| Butterflies | Koruthaialos_rubecula | Overall | EN | 15 |
| Butterflies | Lampides_boeticus | Facebook | LC | 66 |
| Butterflies | Lampides_boeticus | GBIF | LC | 5 |
| Butterflies | Lampides_boeticus | Overall | LC | 71 |
| Butterflies | Lamproptera_curius | Facebook | EN | 22 |
| Butterflies | Lamproptera_curius | GBIF | EN | 1 |
| Butterflies | Lamproptera_curius | Overall | EN | 23 |
| Butterflies | Lebadea_martha | Facebook | VU | 48 |
| Butterflies | Lebadea_martha | GBIF | VU | 11 |
| Butterflies | Lebadea_martha | Overall | VU | 59 |
| Butterflies | Leptosia_nina | Facebook | LC | 69 |
| Butterflies | Leptosia_nina | GBIF | LC | 13 |
| Butterflies | Leptosia_nina | Overall | LC | 82 |
| Butterflies | Leptotes_plinius | Facebook | LC | 51 |
| Butterflies | Leptotes_plinius | Overall | LC | 51 |
| Butterflies | Lethe_europa | Facebook | VU | 56 |
| Butterflies | Lethe_europa | GBIF | VU | 3 |
| Butterflies | Lethe_europa | Overall | VU | 59 |
| Butterflies | Lethe_mekara | Facebook | EN | 11 |
| Butterflies | Lethe_mekara | GBIF | EN | 1 |
| Butterflies | Lethe_mekara | Overall | EN | 12 |
| Butterflies | Lethe_vindhya | Facebook | VU | 1 |
| Butterflies | Lethe_vindhya | Overall | VU | 1 |
| Butterflies | Lexias_cyanipardus | Facebook | EN | 1 |
| Butterflies | Lexias_cyanipardus | Overall | EN | 1 |
| Butterflies | Lexias_dirtea | Facebook | EN | 9 |
| Butterflies | Lexias_dirtea | Overall | EN | 9 |
| Butterflies | Loxura_atymnus | Facebook | VU | 46 |
| Butterflies | Loxura_atymnus | GBIF | VU | 3 |
| Butterflies | Loxura_atymnus | Overall | VU | 49 |
| Butterflies | Mahathala_ameria | Facebook | VU | 36 |
| Butterflies | Mahathala_ameria | Overall | VU | 36 |
| Butterflies | Matapa_aria | Facebook | LC | 49 |
| Butterflies | Matapa_aria | GBIF | LC | 3 |
| Butterflies | Matapa_aria | Overall | LC | 52 |
| Butterflies | Matapa_druna | Facebook | EN | 3 |
| Butterflies | Matapa_druna | Overall | EN | 3 |
| Butterflies | Matapa_sasivarna | Facebook | VU | 6 |
| Butterflies | Matapa_sasivarna | Overall | VU | 6 |
| Butterflies | Megisba_malaya | Facebook | EN | 50 |
| Butterflies | Megisba_malaya | GBIF | EN | 9 |
| Butterflies | Megisba_malaya | Overall | EN | 59 |
| Butterflies | Melanitis_leda | Facebook | LC | 72 |
| Butterflies | Melanitis_leda | GBIF | LC | 27 |
| Butterflies | Melanitis_leda | Overall | LC | 99 |
| Butterflies | Miletus_chinensis | Facebook | EN | 4 |
| Butterflies | Miletus_chinensis | Overall | EN | 4 |
| Butterflies | Moduza_procris | Facebook | LC | 60 |
| Butterflies | Moduza_procris | GBIF | LC | 3 |
| Butterflies | Moduza_procris | Overall | LC | 63 |
| Butterflies | Mooreana_trichoneura | Facebook | EN | 6 |
| Butterflies | Mooreana_trichoneura | Overall | EN | 6 |
| Butterflies | Mycalesis_anaxias | Facebook | EN | 11 |
| Butterflies | Mycalesis_anaxias | GBIF | EN | 3 |
| Butterflies | Mycalesis_anaxias | Overall | EN | 14 |
| Butterflies | Mycalesis_gotama | Facebook | VU | 16 |
| Butterflies | Mycalesis_gotama | GBIF | VU | 2 |
| Butterflies | Mycalesis_gotama | Overall | VU | 18 |
| Butterflies | Mycalesis_malsara | GBIF | EN | 2 |
| Butterflies | Mycalesis_malsara | Overall | EN | 2 |
| Butterflies | Mycalesis_mineus | Facebook | LC | 50 |
| Butterflies | Mycalesis_mineus | GBIF | LC | 8 |
| Butterflies | Mycalesis_mineus | Overall | LC | 58 |
| Butterflies | Mycalesis_perseus | Facebook | VU | 34 |
| Butterflies | Mycalesis_perseus | GBIF | VU | 6 |
| Butterflies | Mycalesis_perseus | Overall | VU | 40 |
| Butterflies | Nacaduba_beroe | Facebook | LC | 2 |
| Butterflies | Nacaduba_beroe | Overall | LC | 2 |
| Butterflies | Neopithecops_zalmora | Facebook | LC | 67 |
| Butterflies | Neopithecops_zalmora | GBIF | LC | 6 |
| Butterflies | Neopithecops_zalmora | Overall | LC | 73 |
| Butterflies | Neptis_clinia | Facebook | VU | 11 |
| Butterflies | Neptis_clinia | GBIF | VU | 3 |
| Butterflies | Neptis_clinia | Overall | VU | 14 |
| Butterflies | Neptis_harita | Facebook | EN | 9 |
| Butterflies | Neptis_harita | Overall | EN | 9 |
| Butterflies | Neptis_hylas | Facebook | LC | 48 |
| Butterflies | Neptis_hylas | GBIF | LC | 5 |
| Butterflies | Neptis_hylas | Overall | LC | 53 |
| Butterflies | Neptis_jumbah | Facebook | LC | 46 |
| Butterflies | Neptis_jumbah | GBIF | LC | 2 |
| Butterflies | Neptis_jumbah | Overall | LC | 48 |
| Butterflies | Neptis_magadha | Facebook | EN | 1 |
| Butterflies | Neptis_magadha | Overall | EN | 1 |
| Butterflies | Neptis_soma | Facebook | VU | 7 |
| Butterflies | Neptis_soma | Overall | VU | 7 |
| Butterflies | Notocrypta_curvifascia | Facebook | EN | 25 |
| Butterflies | Notocrypta_curvifascia | Overall | EN | 25 |
| Butterflies | Notocrypta_paralysos | Facebook | LC | 22 |
| Butterflies | Notocrypta_paralysos | GBIF | LC | 1 |
| Butterflies | Notocrypta_paralysos | Overall | LC | 23 |
| Butterflies | Odontoptilum_angulata | Facebook | LC | 52 |
| Butterflies | Odontoptilum_angulata | GBIF | LC | 5 |
| Butterflies | Odontoptilum_angulata | Overall | LC | 57 |
| Butterflies | Oriens_gola | Facebook | LC | 41 |
| Butterflies | Oriens_gola | Overall | LC | 41 |
| Butterflies | Oriens_goloides | Facebook | VU | 21 |
| Butterflies | Oriens_goloides | Overall | VU | 21 |
| Butterflies | Orsotriaena_medus | Facebook | VU | 35 |
| Butterflies | Orsotriaena_medus | GBIF | VU | 4 |
| Butterflies | Orsotriaena_medus | Overall | VU | 39 |
| Butterflies | Pachliopta_aristolochiae | Facebook | LC | 71 |
| Butterflies | Pachliopta_aristolochiae | GBIF | LC | 4 |
| Butterflies | Pachliopta_aristolochiae | Overall | LC | 75 |
| Butterflies | Pachliopta_hector | Facebook | EN | 19 |
| Butterflies | Pachliopta_hector | Overall | EN | 19 |
| Butterflies | Pantoporia_hordonia | Facebook | VU | 42 |
| Butterflies | Pantoporia_hordonia | GBIF | VU | 2 |
| Butterflies | Pantoporia_hordonia | Overall | VU | 44 |
| Butterflies | Pantoporia_paraka | Facebook | EN | 3 |
| Butterflies | Pantoporia_paraka | Overall | EN | 3 |
| Butterflies | Papilio_castor | Facebook | EN | 4 |
| Butterflies | Papilio_castor | Overall | EN | 4 |
| Butterflies | Papilio_demoleus | Facebook | LC | 89 |
| Butterflies | Papilio_demoleus | GBIF | LC | 13 |
| Butterflies | Papilio_demoleus | Overall | LC | 102 |
| Butterflies | Papilio_helenus | Facebook | VU | 44 |
| Butterflies | Papilio_helenus | GBIF | VU | 2 |
| Butterflies | Papilio_helenus | Overall | VU | 46 |
| Butterflies | Papilio_memnon | Facebook | LC | 55 |
| Butterflies | Papilio_memnon | GBIF | LC | 7 |
| Butterflies | Papilio_memnon | Overall | LC | 62 |
| Butterflies | Papilio_nephelus | Facebook | VU | 46 |
| Butterflies | Papilio_nephelus | GBIF | VU | 2 |
| Butterflies | Papilio_nephelus | Overall | VU | 48 |
| Butterflies | Papilio_polymnestor | Facebook | LC | 61 |
| Butterflies | Papilio_polymnestor | GBIF | LC | 4 |
| Butterflies | Papilio_polymnestor | Overall | LC | 65 |
| Butterflies | Papilio_polytes | Facebook | LC | 105 |
| Butterflies | Papilio_polytes | GBIF | LC | 16 |
| Butterflies | Papilio_polytes | Overall | LC | 121 |
| Butterflies | Parantica_aglea | Facebook | VU | 38 |
| Butterflies | Parantica_aglea | GBIF | VU | 2 |
| Butterflies | Parantica_aglea | Overall | VU | 40 |
| Butterflies | Parantica_melaneus | Facebook | EN | 9 |
| Butterflies | Parantica_melaneus | Overall | EN | 9 |
| Butterflies | Pareronia_ceylanica | Facebook | LC | 2 |
| Butterflies | Pareronia_ceylanica | Overall | LC | 2 |
| Butterflies | Pareronia_hippia | Facebook | VU | 46 |
| Butterflies | Pareronia_hippia | Overall | VU | 46 |
| Butterflies | Parnara_bada | Facebook | EN | 22 |
| Butterflies | Parnara_bada | Overall | EN | 22 |
| Butterflies | Parnara_guttatus | Facebook | LC | 36 |
| Butterflies | Parnara_guttatus | Overall | LC | 36 |
| Butterflies | Parthenos_sylvia | Facebook | VU | 54 |
| Butterflies | Parthenos_sylvia | GBIF | VU | 2 |
| Butterflies | Parthenos_sylvia | Overall | VU | 56 |
| Butterflies | Pathysa_antiphates | Facebook | VU | 48 |
| Butterflies | Pathysa_antiphates | Overall | VU | 48 |
| Butterflies | Pelopidas_agna | Facebook | LC | 21 |
| Butterflies | Pelopidas_agna | GBIF | LC | 1 |
| Butterflies | Pelopidas_agna | Overall | LC | 22 |
| Butterflies | Pelopidas_assamensis | Facebook | EN | 5 |
| Butterflies | Pelopidas_assamensis | Overall | EN | 5 |
| Butterflies | Pelopidas_conjuncta | Facebook | LC | 27 |
| Butterflies | Pelopidas_conjuncta | Overall | LC | 27 |
| Butterflies | Pelopidas_mathias | Facebook | VU | 11 |
| Butterflies | Pelopidas_mathias | Overall | VU | 11 |
| Butterflies | Petrelaea_dana | Facebook | EN | 1 |
| Butterflies | Petrelaea_dana | Overall | EN | 1 |
| Butterflies | Phalanta_phalantha | Facebook | LC | 61 |
| Butterflies | Phalanta_phalantha | GBIF | LC | 5 |
| Butterflies | Phalanta_phalantha | Overall | LC | 66 |
| Butterflies | Pieris_canidia | Facebook | LC | 51 |
| Butterflies | Pieris_canidia | GBIF | LC | 3 |
| Butterflies | Pieris_canidia | Overall | LC | 54 |
| Butterflies | Polytremis_lubricans | Facebook | EN | 3 |
| Butterflies | Polytremis_lubricans | GBIF | EN | 3 |
| Butterflies | Polytremis_lubricans | Overall | EN | 6 |
| Butterflies | Polyura_arja | Facebook | EN | 16 |
| Butterflies | Polyura_arja | Overall | EN | 16 |
| Butterflies | Polyura_athamas | Facebook | LC | 44 |
| Butterflies | Polyura_athamas | GBIF | LC | 2 |
| Butterflies | Polyura_athamas | Overall | LC | 46 |
| Butterflies | Polyura_delphis | Facebook | EN | 41 |
| Butterflies | Polyura_delphis | GBIF | EN | 1 |
| Butterflies | Polyura_delphis | Overall | EN | 42 |
| Butterflies | Polyura_schreiber | Facebook | EN | 1 |
| Butterflies | Polyura_schreiber | Overall | EN | 1 |
| Butterflies | Poritia_hewitsoni | Facebook | EN | 3 |
| Butterflies | Poritia_hewitsoni | Overall | EN | 3 |
| Butterflies | Prosotas_dubiosa | Facebook | VU | 49 |
| Butterflies | Prosotas_dubiosa | GBIF | VU | 2 |
| Butterflies | Prosotas_dubiosa | Overall | VU | 51 |
| Butterflies | Prosotas_lutea | Facebook | EN | 38 |
| Butterflies | Prosotas_lutea | GBIF | EN | 2 |
| Butterflies | Prosotas_lutea | Overall | EN | 40 |
| Butterflies | Prosotas_nora | Facebook | LC | 51 |
| Butterflies | Prosotas_nora | GBIF | LC | 1 |
| Butterflies | Prosotas_nora | Overall | LC | 52 |
| Butterflies | Pseudergolis_wedah | Facebook | EN | 1 |
| Butterflies | Pseudergolis_wedah | Overall | EN | 1 |
| Butterflies | Pseudocoladenia_dan | Facebook | EN | 40 |
| Butterflies | Pseudocoladenia_dan | GBIF | EN | 2 |
| Butterflies | Pseudocoladenia_dan | Overall | EN | 42 |
| Butterflies | Pseudozizeeria_maha | Facebook | LC | 75 |
| Butterflies | Pseudozizeeria_maha | GBIF | LC | 11 |
| Butterflies | Pseudozizeeria_maha | Overall | LC | 86 |
| Butterflies | Psolos_fuligo | Facebook | EN | 29 |
| Butterflies | Psolos_fuligo | GBIF | EN | 1 |
| Butterflies | Psolos_fuligo | Overall | EN | 30 |
| Butterflies | Rachana_jalindra | Facebook | EN | 1 |
| Butterflies | Rachana_jalindra | Overall | EN | 1 |
| Butterflies | Rapala_dieneces | Facebook | EN | 6 |
| Butterflies | Rapala_dieneces | Overall | EN | 6 |
| Butterflies | Rapala_iarbus | Facebook | VU | 23 |
| Butterflies | Rapala_iarbus | Overall | VU | 23 |
| Butterflies | Rapala_manea | Facebook | LC | 53 |
| Butterflies | Rapala_manea | GBIF | LC | 2 |
| Butterflies | Rapala_manea | Overall | LC | 55 |
| Butterflies | Rapala_pheretima | Facebook | VU | 46 |
| Butterflies | Rapala_pheretima | Overall | VU | 46 |
| Butterflies | Rapala_varuna | Facebook | VU | 28 |
| Butterflies | Rapala_varuna | Overall | VU | 28 |
| Butterflies | Rathinda_amor | Facebook | VU | 45 |
| Butterflies | Rathinda_amor | Overall | VU | 45 |
| Butterflies | Remelana_jangala | Facebook | VU | 46 |
| Butterflies | Remelana_jangala | Overall | VU | 46 |
| Butterflies | Rohana_parisatis | Facebook | EN | 14 |
| Butterflies | Rohana_parisatis | Overall | EN | 14 |
| Butterflies | Sarangesa_dasahara | Facebook | VU | 49 |
| Butterflies | Sarangesa_dasahara | GBIF | VU | 1 |
| Butterflies | Sarangesa_dasahara | Overall | VU | 50 |
| Butterflies | Sebastonyma_dolopia | Facebook | EN | 5 |
| Butterflies | Sebastonyma_dolopia | Overall | EN | 5 |
| Butterflies | Spalgis_epius | Facebook | EN | 52 |
| Butterflies | Spalgis_epius | Overall | EN | 52 |
| Butterflies | Spialia_galba | Facebook | LC | 54 |
| Butterflies | Spialia_galba | Overall | LC | 54 |
| Butterflies | Spindasis_ictis | Facebook | EN | 31 |
| Butterflies | Spindasis_ictis | Overall | EN | 31 |
| Butterflies | Spindasis_lohita | Facebook | VU | 41 |
| Butterflies | Spindasis_lohita | Overall | VU | 41 |
| Butterflies | Spindasis_syama | Facebook | VU | 43 |
| Butterflies | Spindasis_syama | Overall | VU | 43 |
| Butterflies | Spindasis_vulcanus | Facebook | LC | 49 |
| Butterflies | Spindasis_vulcanus | Overall | LC | 49 |
| Butterflies | Stibochiona_nicea | Facebook | VU | 25 |
| Butterflies | Stibochiona_nicea | GBIF | VU | 3 |
| Butterflies | Stibochiona_nicea | Overall | VU | 28 |
| Butterflies | Suastus_gremius | Facebook | EN | 15 |
| Butterflies | Suastus_gremius | GBIF | EN | 1 |
| Butterflies | Suastus_gremius | Overall | EN | 16 |
| Butterflies | Surendra_quercetorum | Facebook | EN | 44 |
| Butterflies | Surendra_quercetorum | Overall | EN | 44 |
| Butterflies | Symbrenthia_lilaea | Facebook | EN | 15 |
| Butterflies | Symbrenthia_lilaea | Overall | EN | 15 |
| Butterflies | Tagiades_gana | Facebook | VU | 12 |
| Butterflies | Tagiades_gana | Overall | VU | 12 |
| Butterflies | Tagiades_japetus | Facebook | VU | 55 |
| Butterflies | Tagiades_japetus | GBIF | VU | 5 |
| Butterflies | Tagiades_japetus | Overall | VU | 60 |
| Butterflies | Tagiades_litigiosa | Facebook | EN | 23 |
| Butterflies | Tagiades_litigiosa | GBIF | EN | 1 |
| Butterflies | Tagiades_litigiosa | Overall | EN | 24 |
| Butterflies | Tajuria_cippus | Facebook | EN | 49 |
| Butterflies | Tajuria_cippus | Overall | EN | 49 |
| Butterflies | Tajuria_jehana | Facebook | DD | 2 |
| Butterflies | Tajuria_jehana | Overall | DD | 2 |
| Butterflies | Tanaecia_jahnu | Facebook | EN | 7 |
| Butterflies | Tanaecia_jahnu | Overall | EN | 7 |
| Butterflies | Tanaecia_julii | Facebook | VU | 46 |
| Butterflies | Tanaecia_julii | GBIF | VU | 4 |
| Butterflies | Tanaecia_julii | Overall | VU | 50 |
| Butterflies | Tanaecia_lepidea | Facebook | VU | 46 |
| Butterflies | Tanaecia_lepidea | GBIF | VU | 2 |
| Butterflies | Tanaecia_lepidea | Overall | VU | 48 |
| Butterflies | Tarucus_callinara | Facebook | EN | 5 |
| Butterflies | Tarucus_callinara | Overall | EN | 5 |
| Butterflies | Tarucus_nara | Facebook | EN | 23 |
| Butterflies | Tarucus_nara | Overall | EN | 23 |
| Butterflies | Taxila_haquinus | Facebook | EN | 15 |
| Butterflies | Taxila_haquinus | GBIF | EN | 1 |
| Butterflies | Taxila_haquinus | Overall | EN | 16 |
| Butterflies | Telicota_bambusae | Facebook | VU | 38 |
| Butterflies | Telicota_bambusae | Overall | VU | 38 |
| Butterflies | Thaumantis_diores | Facebook | EN | 3 |
| Butterflies | Thaumantis_diores | Overall | EN | 3 |
| Butterflies | Tirumala_limniace | Facebook | LC | 52 |
| Butterflies | Tirumala_limniace | GBIF | LC | 5 |
| Butterflies | Tirumala_limniace | Overall | LC | 57 |
| Butterflies | Tirumala_septentrionis | Facebook | VU | 16 |
| Butterflies | Tirumala_septentrionis | Overall | VU | 16 |
| Butterflies | Troides_aeacus | Facebook | EN | 1 |
| Butterflies | Troides_aeacus | Overall | EN | 1 |
| Butterflies | Troides_helena | Facebook | VU | 41 |
| Butterflies | Troides_helena | GBIF | VU | 3 |
| Butterflies | Troides_helena | Overall | VU | 44 |
| Butterflies | Udaspes_folus | Facebook | LC | 58 |
| Butterflies | Udaspes_folus | GBIF | LC | 6 |
| Butterflies | Udaspes_folus | Overall | LC | 64 |
| Butterflies | Vagrans_egista | Facebook | VU | 33 |
| Butterflies | Vagrans_egista | Overall | VU | 33 |
| Butterflies | Vanessa_cardui | Facebook | EN | 41 |
| Butterflies | Vanessa_cardui | Overall | EN | 41 |
| Butterflies | Vindula_erota | Facebook | EN | 16 |
| Butterflies | Vindula_erota | Overall | EN | 16 |
| Butterflies | Virachola_isocrates | Facebook | EN | 17 |
| Butterflies | Virachola_isocrates | GBIF | EN | 1 |
| Butterflies | Virachola_isocrates | Overall | EN | 18 |
| Butterflies | Ypthima_baldus | Facebook | VU | 43 |
| Butterflies | Ypthima_baldus | GBIF | VU | 3 |
| Butterflies | Ypthima_baldus | Overall | VU | 46 |
| Butterflies | Ypthima_huebneri | Facebook | LC | 48 |
| Butterflies | Ypthima_huebneri | GBIF | LC | 18 |
| Butterflies | Ypthima_huebneri | Overall | LC | 66 |
| Butterflies | Ypthima_inica | Facebook | EN | 3 |
| Butterflies | Ypthima_inica | Overall | EN | 3 |
| Butterflies | Zeltus_amasa | Facebook | EN | 20 |
| Butterflies | Zeltus_amasa | Overall | EN | 20 |
| Butterflies | Zemeros_flegyas | Facebook | LC | 53 |
| Butterflies | Zemeros_flegyas | GBIF | LC | 9 |
| Butterflies | Zemeros_flegyas | Overall | LC | 62 |
| Butterflies | Zizeeria_karsandra | Facebook | LC | 46 |
| Butterflies | Zizeeria_karsandra | GBIF | LC | 18 |
| Butterflies | Zizeeria_karsandra | Overall | LC | 64 |
| Butterflies | Zizina_otis | Facebook | LC | 55 |
| Butterflies | Zizina_otis | GBIF | LC | 5 |
| Butterflies | Zizina_otis | Overall | LC | 60 |
| Butterflies | Zizula_hylax | Facebook | LC | 37 |
| Butterflies | Zizula_hylax | GBIF | LC | 2 |
| Butterflies | Zizula_hylax | Overall | LC | 39 |
| Crustaceans | Metapenaeus_affinis | GBIF | DD | 2 |
| Crustaceans | Metapenaeus_affinis | Overall | DD | 2 |
| Crustaceans | Penaeus_indicus | GBIF | LC | 6 |
| Crustaceans | Penaeus_indicus | Overall | LC | 6 |
| Crustaceans | Penaeus_semisulcatus | GBIF | LC | 21 |
| Crustaceans | Penaeus_semisulcatus | Overall | LC | 21 |
| Crustaceans | Solenocera_melantho | GBIF | DD | 2 |
| Crustaceans | Solenocera_melantho | Overall | DD | 2 |
| Fishes | Amblyceps_laticeps | GBIF | VU | 2 |
| Fishes | Amblyceps_laticeps | Overall | VU | 2 |
| Fishes | Anabas_cobojius | GBIF | DD | 2 |
| Fishes | Anabas_cobojius | Overall | DD | 2 |
| Fishes | Badis_chittagongis | GBIF | DD | 16 |
| Fishes | Badis_chittagongis | Overall | DD | 16 |
| Fishes | Balitora_brucei | GBIF | DD | 3 |
| Fishes | Balitora_brucei | Overall | DD | 3 |
| Fishes | Barilius_barila | GBIF | DD | 6 |
| Fishes | Barilius_barila | Overall | DD | 6 |
| Fishes | Danio_annulosus | GBIF | DD | 7 |
| Fishes | Danio_annulosus | Overall | DD | 7 |
| Fishes | Labeo_dyocheilus | GBIF | DD | 1 |
| Fishes | Labeo_dyocheilus | Overall | DD | 1 |
| Fishes | Microphis_deocata | GBIF | VU | 1 |
| Fishes | Microphis_deocata | Overall | VU | 1 |
| Fishes | Nangra_bucculenta | GBIF | DD | 1 |
| Fishes | Nangra_bucculenta | Overall | DD | 1 |
| Fishes | Salmostoma_sardinella | GBIF | DD | 4 |
| Fishes | Salmostoma_sardinella | Overall | DD | 4 |
| Mammals | Aonyx_cinerea | Facebook | EN | 13 |
| Mammals | Aonyx_cinerea | Overall | EN | 13 |
| Mammals | Arctictis_binturong | Facebook | VU | 4 |
| Mammals | Arctictis_binturong | Overall | VU | 4 |
| Mammals | Arctonyx_collaris | Facebook | VU | 8 |
| Mammals | Arctonyx_collaris | Overall | VU | 8 |
| Mammals | Atherurus_macrourus | Facebook | DD | 3 |
| Mammals | Atherurus_macrourus | Overall | DD | 3 |
| Mammals | Axis_axis | Facebook | LC | 15 |
| Mammals | Axis_axis | GBIF | LC | 12 |
| Mammals | Axis_axis | Overall | LC | 27 |
| Mammals | Bandicota_bengalensis | Facebook | LC | 2 |
| Mammals | Bandicota_bengalensis | GBIF | LC | 3 |
| Mammals | Bandicota_bengalensis | Overall | LC | 5 |
| Mammals | Bandicota_indica | Facebook | LC | 4 |
| Mammals | Bandicota_indica | Overall | LC | 4 |
| Mammals | Bos_gaurus | Facebook | CR | 3 |
| Mammals | Bos_gaurus | Overall | CR | 3 |
| Mammals | Callosciurus_erythraeus | Facebook | LC | 4 |
| Mammals | Callosciurus_erythraeus | GBIF | LC | 7 |
| Mammals | Callosciurus_erythraeus | Overall | LC | 11 |
| Mammals | Callosciurus_pygerythrus | Facebook | LC | 15 |
| Mammals | Callosciurus_pygerythrus | GBIF | LC | 31 |
| Mammals | Callosciurus_pygerythrus | Overall | LC | 46 |
| Mammals | Canis_aureus | Facebook | LC | 26 |
| Mammals | Canis_aureus | GBIF | LC | 12 |
| Mammals | Canis_aureus | Overall | LC | 38 |
| Mammals | Capricornis_rubidus | Facebook | EN | 4 |
| Mammals | Capricornis_rubidus | Overall | EN | 4 |
| Mammals | Catopuma_temminckii | Facebook | VU | 7 |
| Mammals | Catopuma_temminckii | GBIF | VU | 1 |
| Mammals | Catopuma_temminckii | Overall | VU | 8 |
| Mammals | Cuon_alpinus | Facebook | EN | 6 |
| Mammals | Cuon_alpinus | GBIF | EN | 1 |
| Mammals | Cuon_alpinus | Overall | EN | 7 |
| Mammals | Cynopterus_sphinx | Facebook | LC | 3 |
| Mammals | Cynopterus_sphinx | GBIF | LC | 2 |
| Mammals | Cynopterus_sphinx | Overall | LC | 5 |
| Mammals | Dremomys_lokriah | Facebook | LC | 4 |
| Mammals | Dremomys_lokriah | Overall | LC | 4 |
| Mammals | Elephas_maximus | Facebook | CR | 41 |
| Mammals | Elephas_maximus | GBIF | CR | 1 |
| Mammals | Elephas_maximus | Overall | CR | 42 |
| Mammals | Eonycteris_spelaea | Facebook | DD | 1 |
| Mammals | Eonycteris_spelaea | Overall | DD | 1 |
| Mammals | Felis_chaus | Facebook | NT | 22 |
| Mammals | Felis_chaus | GBIF | NT | 8 |
| Mammals | Felis_chaus | Overall | NT | 30 |
| Mammals | Funambulus_pennantii | Facebook | LC | 7 |
| Mammals | Funambulus_pennantii | GBIF | LC | 15 |
| Mammals | Funambulus_pennantii | Overall | LC | 22 |
| Mammals | Helarctos_malayanus | Facebook | CR | 5 |
| Mammals | Helarctos_malayanus | Overall | CR | 5 |
| Mammals | Herpestes_auropunctatus | Facebook | LC | 8 |
| Mammals | Herpestes_auropunctatus | GBIF | LC | 2 |
| Mammals | Herpestes_auropunctatus | Overall | LC | 10 |
| Mammals | Herpestes_edwardsii | Facebook | LC | 3 |
| Mammals | Herpestes_edwardsii | Overall | LC | 3 |
| Mammals | Herpestes_urva | Facebook | NT | 9 |
| Mammals | Herpestes_urva | Overall | NT | 9 |
| Mammals | Hipposideros_larvatus | Facebook | LC | 5 |
| Mammals | Hipposideros_larvatus | Overall | LC | 5 |
| Mammals | Hoolock_hoolock | Facebook | CR | 17 |
| Mammals | Hoolock_hoolock | GBIF | CR | 5 |
| Mammals | Hoolock_hoolock | Overall | CR | 22 |
| Mammals | Hylopetes_alboniger | Facebook | EN | 11 |
| Mammals | Hylopetes_alboniger | Overall | EN | 11 |
| Mammals | Hystrix_indica | Facebook | LC | 1 |
| Mammals | Hystrix_indica | Overall | LC | 1 |
| Mammals | Lepus_nigricollis | Facebook | EN | 18 |
| Mammals | Lepus_nigricollis | Overall | EN | 18 |
| Mammals | Lutrogale_perspicillata | Facebook | CR | 3 |
| Mammals | Lutrogale_perspicillata | GBIF | CR | 1 |
| Mammals | Lutrogale_perspicillata | Overall | CR | 4 |
| Mammals | Lyroderma_lyra | Facebook | LC | 2 |
| Mammals | Lyroderma_lyra | Overall | LC | 2 |
| Mammals | Macaca_fascicularis | Facebook | CR | 1 |
| Mammals | Macaca_fascicularis | Overall | CR | 1 |
| Mammals | Macaca_leonina | Facebook | EN | 17 |
| Mammals | Macaca_leonina | GBIF | EN | 7 |
| Mammals | Macaca_leonina | Overall | EN | 24 |
| Mammals | Macaca_mulatta | Facebook | VU | 43 |
| Mammals | Macaca_mulatta | GBIF | VU | 22 |
| Mammals | Macaca_mulatta | Overall | VU | 65 |
| Mammals | Manis_pentadactyla | Facebook | CR | 2 |
| Mammals | Manis_pentadactyla | Overall | CR | 2 |
| Mammals | Martes_flavigula | Facebook | VU | 5 |
| Mammals | Martes_flavigula | GBIF | VU | 1 |
| Mammals | Martes_flavigula | Overall | VU | 6 |
| Mammals | Millardia_meltada | Facebook | LC | 1 |
| Mammals | Millardia_meltada | Overall | LC | 1 |
| Mammals | Muntiacus_muntjak | Facebook | EN | 30 |
| Mammals | Muntiacus_muntjak | Overall | EN | 30 |
| Mammals | Mus_musculus | Facebook | LC | 2 |
| Mammals | Mus_musculus | GBIF | LC | 371 |
| Mammals | Mus_musculus | Overall | LC | 373 |
| Mammals | Neofelis_nebulosa | Facebook | CR | 10 |
| Mammals | Neofelis_nebulosa | GBIF | CR | 1 |
| Mammals | Neofelis_nebulosa | Overall | CR | 11 |
| Mammals | Neophocaena_phocaenoides | Facebook | NT | 1 |
| Mammals | Neophocaena_phocaenoides | Overall | NT | 1 |
| Mammals | Nesokia_indica | Facebook | DD | 1 |
| Mammals | Nesokia_indica | Overall | DD | 1 |
| Mammals | Nycticebus_bengalensis | Facebook | EN | 24 |
| Mammals | Nycticebus_bengalensis | GBIF | EN | 2 |
| Mammals | Nycticebus_bengalensis | Overall | EN | 26 |
| Mammals | Orcaella_brevirostris | Facebook | NT | 5 |
| Mammals | Orcaella_brevirostris | GBIF | NT | 1 |
| Mammals | Orcaella_brevirostris | Overall | NT | 6 |
| Mammals | Paguma_larvata | Facebook | VU | 26 |
| Mammals | Paguma_larvata | Overall | VU | 26 |
| Mammals | Panthera_pardus | Facebook | CR | 8 |
| Mammals | Panthera_pardus | GBIF | CR | 1 |
| Mammals | Panthera_pardus | Overall | CR | 9 |
| Mammals | Panthera_tigris | Facebook | CR | 7 |
| Mammals | Panthera_tigris | GBIF | CR | 3 |
| Mammals | Panthera_tigris | Overall | CR | 10 |
| Mammals | Paradoxurus_hermaphroditus | Facebook | LC | 6 |
| Mammals | Paradoxurus_hermaphroditus | GBIF | LC | 1 |
| Mammals | Paradoxurus_hermaphroditus | Overall | LC | 7 |
| Mammals | Pardofelis_marmorata | Facebook | DD | 3 |
| Mammals | Pardofelis_marmorata | GBIF | DD | 1 |
| Mammals | Pardofelis_marmorata | Overall | DD | 4 |
| Mammals | Petaurista_magnificus | Facebook | NT | 2 |
| Mammals | Petaurista_magnificus | Overall | NT | 2 |
| Mammals | Petaurista_petaurista | Facebook | DD | 2 |
| Mammals | Petaurista_petaurista | Overall | DD | 2 |
| Mammals | Pipistrellus_tenuis | GBIF | LC | 22 |
| Mammals | Pipistrellus_tenuis | Overall | LC | 22 |
| Mammals | Platanista_gangetica | Facebook | VU | 41 |
| Mammals | Platanista_gangetica | GBIF | VU | 7 |
| Mammals | Platanista_gangetica | Overall | VU | 48 |
| Mammals | Prionailurus_bengalensis | Facebook | NT | 19 |
| Mammals | Prionailurus_bengalensis | GBIF | NT | 2 |
| Mammals | Prionailurus_bengalensis | Overall | NT | 21 |
| Mammals | Prionailurus_viverrinus | Facebook | EN | 66 |
| Mammals | Prionailurus_viverrinus | GBIF | EN | 3 |
| Mammals | Prionailurus_viverrinus | Overall | EN | 69 |
| Mammals | Pteropus_giganteus | Facebook | LC | 9 |
| Mammals | Pteropus_giganteus | GBIF | LC | 17 |
| Mammals | Pteropus_giganteus | Overall | LC | 26 |
| Mammals | Rattus_rattus | Facebook | LC | 1 |
| Mammals | Rattus_rattus | GBIF | LC | 347 |
| Mammals | Rattus_rattus | Overall | LC | 348 |
| Mammals | Ratufa_bicolor | Facebook | VU | 7 |
| Mammals | Ratufa_bicolor | Overall | VU | 7 |
| Mammals | Rhinolophus_lepidus | Facebook | LC | 1 |
| Mammals | Rhinolophus_lepidus | Overall | LC | 1 |
| Mammals | Rousettus_leschenaultii | GBIF | LC | 1 |
| Mammals | Rousettus_leschenaultii | Overall | LC | 1 |
| Mammals | Rusa_unicolor | Facebook | CR | 2 |
| Mammals | Rusa_unicolor | GBIF | CR | 1 |
| Mammals | Rusa_unicolor | Overall | CR | 3 |
| Mammals | Saccolaimus_saccolaimus | Facebook | DD | 2 |
| Mammals | Saccolaimus_saccolaimus | Overall | DD | 2 |
| Mammals | Semnopithecus_entellus | Facebook | EN | 6 |
| Mammals | Semnopithecus_entellus | GBIF | EN | 1 |
| Mammals | Semnopithecus_entellus | Overall | EN | 7 |
| Mammals | Sousa_chinensis | Facebook | LC | 1 |
| Mammals | Sousa_chinensis | Overall | LC | 1 |
| Mammals | Suncus_murinus | Facebook | LC | 2 |
| Mammals | Suncus_murinus | GBIF | LC | 5 |
| Mammals | Suncus_murinus | Overall | LC | 7 |
| Mammals | Sus_scrofa | Facebook | LC | 10 |
| Mammals | Sus_scrofa | GBIF | LC | 5 |
| Mammals | Sus_scrofa | Overall | LC | 15 |
| Mammals | Trachypithecus_phayrei | Facebook | CR | 26 |
| Mammals | Trachypithecus_phayrei | GBIF | CR | 6 |
| Mammals | Trachypithecus_phayrei | Overall | CR | 32 |
| Mammals | Trachypithecus_pileatus | Facebook | EN | 23 |
| Mammals | Trachypithecus_pileatus | GBIF | EN | 7 |
| Mammals | Trachypithecus_pileatus | Overall | EN | 30 |
| Mammals | Tupaia_glis | Facebook | NT | 1 |
| Mammals | Tupaia_glis | Overall | NT | 1 |
| Mammals | Ursus_thibetanus | Facebook | CR | 3 |
| Mammals | Ursus_thibetanus | GBIF | CR | 1 |
| Mammals | Ursus_thibetanus | Overall | CR | 4 |
| Mammals | Vandeleuria_oleracea | Facebook | LC | 3 |
| Mammals | Vandeleuria_oleracea | Overall | LC | 3 |
| Mammals | Viverra_zibetha | Facebook | NT | 12 |
| Mammals | Viverra_zibetha | Overall | NT | 12 |
| Mammals | Viverricula_indica | Facebook | NT | 5 |
| Mammals | Viverricula_indica | Overall | NT | 5 |
| Mammals | Vulpes_bengalensis | Facebook | VU | 4 |
| Mammals | Vulpes_bengalensis | Overall | VU | 4 |
| Reptiles | Ahaetulla_nasuta | Facebook | LC | 2 |
| Reptiles | Ahaetulla_nasuta | Overall | LC | 2 |
| Reptiles | Ahaetulla_prasina | Facebook | LC | 9 |
| Reptiles | Ahaetulla_prasina | Overall | LC | 9 |
| Reptiles | Amphiesma_platyceps | Facebook | DD | 1 |
| Reptiles | Amphiesma_platyceps | Overall | DD | 1 |
| Reptiles | Amphiesma_stolatum | Facebook | LC | 8 |
| Reptiles | Amphiesma_stolatum | GBIF | LC | 8 |
| Reptiles | Amphiesma_stolatum | Overall | LC | 16 |
| Reptiles | Argyrogena_fasciolata | Facebook | LC | 2 |
| Reptiles | Argyrogena_fasciolata | Overall | LC | 2 |
| Reptiles | Argyrophis_diardii | Facebook | LC | 2 |
| Reptiles | Argyrophis_diardii | GBIF | LC | 1 |
| Reptiles | Argyrophis_diardii | Overall | LC | 3 |
| Reptiles | Atretium_schistosum | Facebook | LC | 4 |
| Reptiles | Atretium_schistosum | Overall | LC | 4 |
| Reptiles | Blythia_reticulata | Facebook | DD | 1 |
| Reptiles | Blythia_reticulata | Overall | DD | 1 |
| Reptiles | Boiga_cyanea | Facebook | LC | 2 |
| Reptiles | Boiga_cyanea | GBIF | LC | 5 |
| Reptiles | Boiga_cyanea | Overall | LC | 7 |
| Reptiles | Boiga_gokool | Facebook | NT | 2 |
| Reptiles | Boiga_gokool | Overall | NT | 2 |
| Reptiles | Boiga_ochracea | Facebook | NT | 2 |
| Reptiles | Boiga_ochracea | GBIF | NT | 3 |
| Reptiles | Boiga_ochracea | Overall | NT | 5 |
| Reptiles | Boiga_siamensis | Facebook | EN | 2 |
| Reptiles | Boiga_siamensis | GBIF | EN | 1 |
| Reptiles | Boiga_siamensis | Overall | EN | 3 |
| Reptiles | Bungarus_caeruleus | Facebook | LC | 6 |
| Reptiles | Bungarus_caeruleus | GBIF | LC | 1 |
| Reptiles | Bungarus_caeruleus | Overall | LC | 7 |
| Reptiles | Bungarus_fasciatus | Facebook | LC | 23 |
| Reptiles | Bungarus_fasciatus | GBIF | LC | 4 |
| Reptiles | Bungarus_fasciatus | Overall | LC | 27 |
| Reptiles | Bungarus_lividus | Facebook | NT | 1 |
| Reptiles | Bungarus_lividus | Overall | NT | 1 |
| Reptiles | Bungarus_niger | Facebook | NT | 5 |
| Reptiles | Bungarus_niger | GBIF | NT | 2 |
| Reptiles | Bungarus_niger | Overall | NT | 7 |
| Reptiles | Bungarus_walli | Facebook | NT | 1 |
| Reptiles | Bungarus_walli | Overall | NT | 1 |
| Reptiles | Calotes_emma | Facebook | LC | 4 |
| Reptiles | Calotes_emma | GBIF | LC | 3 |
| Reptiles | Calotes_emma | Overall | LC | 7 |
| Reptiles | Calotes_jerdoni | Facebook | DD | 1 |
| Reptiles | Calotes_jerdoni | Overall | DD | 1 |
| Reptiles | Cerberus_rynchops | Facebook | LC | 5 |
| Reptiles | Cerberus_rynchops | GBIF | LC | 10 |
| Reptiles | Cerberus_rynchops | Overall | LC | 15 |
| Reptiles | Chrysopelea_ornata | Facebook | LC | 9 |
| Reptiles | Chrysopelea_ornata | Overall | LC | 9 |
| Reptiles | Coelognathus_helena | Facebook | LC | 1 |
| Reptiles | Coelognathus_helena | Overall | LC | 1 |
| Reptiles | Coelognathus_radiatus | Facebook | LC | 10 |
| Reptiles | Coelognathus_radiatus | GBIF | LC | 1 |
| Reptiles | Coelognathus_radiatus | Overall | LC | 11 |
| Reptiles | Crocodylus_porosus | Facebook | EN | 1 |
| Reptiles | Crocodylus_porosus | GBIF | EN | 9 |
| Reptiles | Crocodylus_porosus | Overall | EN | 10 |
| Reptiles | Cuora_mouhotii | Facebook | CR | 1 |
| Reptiles | Cuora_mouhotii | GBIF | CR | 1 |
| Reptiles | Cuora_mouhotii | Overall | CR | 2 |
| Reptiles | Cyclemys_gemeli | Facebook | VU | 1 |
| Reptiles | Cyclemys_gemeli | GBIF | VU | 1 |
| Reptiles | Cyclemys_gemeli | Overall | VU | 2 |
| Reptiles | Cyrtodactylus_ayeyarwadyensis | Facebook | LC | 3 |
| Reptiles | Cyrtodactylus_ayeyarwadyensis | Overall | LC | 3 |
| Reptiles | Daboia_russelii | Facebook | NT | 13 |
| Reptiles | Daboia_russelii | GBIF | NT | 1 |
| Reptiles | Daboia_russelii | Overall | NT | 14 |
| Reptiles | Dendrelaphis_cyanochloris | Facebook | DD | 1 |
| Reptiles | Dendrelaphis_cyanochloris | Overall | DD | 1 |
| Reptiles | Dendrelaphis_pictus | Facebook | LC | 9 |
| Reptiles | Dendrelaphis_pictus | GBIF | LC | 3 |
| Reptiles | Dendrelaphis_pictus | Overall | LC | 12 |
| Reptiles | Dendrelaphis_tristis | Facebook | LC | 2 |
| Reptiles | Dendrelaphis_tristis | GBIF | LC | 1 |
| Reptiles | Dendrelaphis_tristis | Overall | LC | 3 |
| Reptiles | Draco_blanfordii | Facebook | DD | 1 |
| Reptiles | Draco_blanfordii | Overall | DD | 1 |
| Reptiles | Draco_maculatus | Facebook | EN | 1 |
| Reptiles | Draco_maculatus | Overall | EN | 1 |
| Reptiles | Enhydris_enhydris | Facebook | LC | 6 |
| Reptiles | Enhydris_enhydris | GBIF | LC | 33 |
| Reptiles | Enhydris_enhydris | Overall | LC | 39 |
| Reptiles | Eryx_conicus | Facebook | DD | 10 |
| Reptiles | Eryx_conicus | Overall | DD | 10 |
| Reptiles | Eutropis_dissimilis | Facebook | LC | 8 |
| Reptiles | Eutropis_dissimilis | Overall | LC | 8 |
| Reptiles | Eutropis_macularia | Facebook | LC | 12 |
| Reptiles | Eutropis_macularia | GBIF | LC | 1 |
| Reptiles | Eutropis_macularia | Overall | LC | 13 |
| Reptiles | Eutropis_multifasciata | Facebook | LC | 5 |
| Reptiles | Eutropis_multifasciata | GBIF | LC | 6 |
| Reptiles | Eutropis_multifasciata | Overall | LC | 11 |
| Reptiles | Fordonia_leucobalia | Facebook | NT | 1 |
| Reptiles | Fordonia_leucobalia | GBIF | NT | 2 |
| Reptiles | Fordonia_leucobalia | Overall | NT | 3 |
| Reptiles | Gavialis_gangeticus | Facebook | CR | 3 |
| Reptiles | Gavialis_gangeticus | Overall | CR | 3 |
| Reptiles | Gerarda_prevostiana | Facebook | LC | 11 |
| Reptiles | Gerarda_prevostiana | GBIF | LC | 1 |
| Reptiles | Gerarda_prevostiana | Overall | LC | 12 |
| Reptiles | Hemidactylus_bowringii | Facebook | LC | 4 |
| Reptiles | Hemidactylus_bowringii | Overall | LC | 4 |
| Reptiles | Hemidactylus_brookii | Facebook | LC | 1 |
| Reptiles | Hemidactylus_brookii | Overall | LC | 1 |
| Reptiles | Hemidactylus_flaviviridis | Facebook | LC | 1 |
| Reptiles | Hemidactylus_flaviviridis | GBIF | LC | 4 |
| Reptiles | Hemidactylus_flaviviridis | Overall | LC | 5 |
| Reptiles | Hemidactylus_garnotii | Facebook | LC | 1 |
| Reptiles | Hemidactylus_garnotii | Overall | LC | 1 |
| Reptiles | Heosemys_depressa | Facebook | CR | 1 |
| Reptiles | Heosemys_depressa | Overall | CR | 1 |
| Reptiles | Hydrophis_obscurus | Facebook | LC | 1 |
| Reptiles | Hydrophis_obscurus | Overall | LC | 1 |
| Reptiles | Indotestudo_elongata | Facebook | CR | 14 |
| Reptiles | Indotestudo_elongata | GBIF | CR | 4 |
| Reptiles | Indotestudo_elongata | Overall | CR | 18 |
| Reptiles | Indotyphlops_braminus | Facebook | LC | 7 |
| Reptiles | Indotyphlops_braminus | GBIF | LC | 5 |
| Reptiles | Indotyphlops_braminus | Overall | LC | 12 |
| Reptiles | Indotyphlops_porrectus | GBIF | NE | 1 |
| Reptiles | Indotyphlops_porrectus | Overall | NE | 1 |
| Reptiles | Lissemys_punctata | Facebook | LC | 7 |
| Reptiles | Lissemys_punctata | GBIF | LC | 2 |
| Reptiles | Lissemys_punctata | Overall | LC | 9 |
| Reptiles | Lycodon_aulicus | Facebook | LC | 14 |
| Reptiles | Lycodon_aulicus | GBIF | LC | 3 |
| Reptiles | Lycodon_aulicus | Overall | LC | 17 |
| Reptiles | Lycodon_jara | Facebook | LC | 9 |
| Reptiles | Lycodon_jara | GBIF | LC | 2 |
| Reptiles | Lycodon_jara | Overall | LC | 11 |
| Reptiles | Lycodon_zawi | Facebook | LC | 1 |
| Reptiles | Lycodon_zawi | GBIF | LC | 5 |
| Reptiles | Lycodon_zawi | Overall | LC | 6 |
| Reptiles | Lygosoma_lineolatum | Facebook | NT | 2 |
| Reptiles | Lygosoma_lineolatum | Overall | NT | 2 |
| Reptiles | Malayopython_reticulatus | Facebook | CR | 2 |
| Reptiles | Malayopython_reticulatus | GBIF | CR | 1 |
| Reptiles | Malayopython_reticulatus | Overall | CR | 3 |
| Reptiles | Manouria_emys | Facebook | LC | 1 |
| Reptiles | Manouria_emys | Overall | LC | 1 |
| Reptiles | Naja_kaouthia | Facebook | NT | 17 |
| Reptiles | Naja_kaouthia | GBIF | NT | 6 |
| Reptiles | Naja_kaouthia | Overall | NT | 23 |
| Reptiles | Naja_naja | Facebook | NT | 12 |
| Reptiles | Naja_naja | GBIF | NT | 2 |
| Reptiles | Naja_naja | Overall | NT | 14 |
| Reptiles | Nilssonia_hurum | Facebook | LC | 1 |
| Reptiles | Nilssonia_hurum | Overall | LC | 1 |
| Reptiles | Oligodon_albocinctus | Facebook | LC | 5 |
| Reptiles | Oligodon_albocinctus | GBIF | LC | 1 |
| Reptiles | Oligodon_albocinctus | Overall | LC | 6 |
| Reptiles | Oligodon_arnensis | Facebook | DD | 2 |
| Reptiles | Oligodon_arnensis | Overall | DD | 2 |
| Reptiles | Oligodon_cyclurus | Facebook | LC | 2 |
| Reptiles | Oligodon_cyclurus | GBIF | LC | 1 |
| Reptiles | Oligodon_cyclurus | Overall | LC | 3 |
| Reptiles | Oligodon_dorsalis | Facebook | LC | 3 |
| Reptiles | Oligodon_dorsalis | Overall | LC | 3 |
| Reptiles | Ophiophagus_hannah | Facebook | VU | 6 |
| Reptiles | Ophiophagus_hannah | Overall | VU | 6 |
| Reptiles | Oreocryptophis_porphyraceus | Facebook | DD | 1 |
| Reptiles | Oreocryptophis_porphyraceus | Overall | DD | 1 |
| Reptiles | Pangshura_tecta | Facebook | LC | 2 |
| Reptiles | Pangshura_tecta | GBIF | LC | 1 |
| Reptiles | Pangshura_tecta | Overall | LC | 3 |
| Reptiles | Pangshura_tentoria | Facebook | NT | 3 |
| Reptiles | Pangshura_tentoria | Overall | NT | 3 |
| Reptiles | Pareas_monticola | Facebook | LC | 1 |
| Reptiles | Pareas_monticola | GBIF | LC | 2 |
| Reptiles | Pareas_monticola | Overall | LC | 3 |
| Reptiles | Psammodynastes_pulverulentus | Facebook | LC | 6 |
| Reptiles | Psammodynastes_pulverulentus | GBIF | LC | 5 |
| Reptiles | Psammodynastes_pulverulentus | Overall | LC | 11 |
| Reptiles | Ptyas_korros | Facebook | NT | 3 |
| Reptiles | Ptyas_korros | GBIF | NT | 3 |
| Reptiles | Ptyas_korros | Overall | NT | 6 |
| Reptiles | Ptyas_mucosa | Facebook | LC | 13 |
| Reptiles | Ptyas_mucosa | GBIF | LC | 3 |
| Reptiles | Ptyas_mucosa | Overall | LC | 16 |
| Reptiles | Ptyas_nigromarginata | Facebook | VU | 1 |
| Reptiles | Ptyas_nigromarginata | Overall | VU | 1 |
| Reptiles | Ptyctolaemus_gularis | Facebook | EN | 2 |
| Reptiles | Ptyctolaemus_gularis | GBIF | EN | 1 |
| Reptiles | Ptyctolaemus_gularis | Overall | EN | 3 |
| Reptiles | Python_bivittatus | Facebook | VU | 16 |
| Reptiles | Python_bivittatus | GBIF | VU | 5 |
| Reptiles | Python_bivittatus | Overall | VU | 21 |
| Reptiles | Python_molurus | Facebook | DD | 3 |
| Reptiles | Python_molurus | Overall | DD | 3 |
| Reptiles | Rhabdophis_himalayanus | Facebook | VU | 6 |
| Reptiles | Rhabdophis_himalayanus | GBIF | VU | 2 |
| Reptiles | Rhabdophis_himalayanus | Overall | VU | 8 |
| Reptiles | Rhabdophis_subminiatus | Facebook | NT | 20 |
| Reptiles | Rhabdophis_subminiatus | GBIF | NT | 3 |
| Reptiles | Rhabdophis_subminiatus | Overall | NT | 23 |
| Reptiles | Riopa_albopunctata | Facebook | LC | 1 |
| Reptiles | Riopa_albopunctata | GBIF | LC | 4 |
| Reptiles | Riopa_albopunctata | Overall | LC | 5 |
| Reptiles | Riopa_punctata | Facebook | EN | 2 |
| Reptiles | Riopa_punctata | Overall | EN | 2 |
| Reptiles | Scincella_reevesii | Facebook | LC | 1 |
| Reptiles | Scincella_reevesii | Overall | LC | 1 |
| Reptiles | Sphenomorphus_maculatus | Facebook | LC | 2 |
| Reptiles | Sphenomorphus_maculatus | GBIF | LC | 1 |
| Reptiles | Sphenomorphus_maculatus | Overall | LC | 3 |
| Reptiles | Takydromus_khasiensis | Facebook | LC | 2 |
| Reptiles | Takydromus_khasiensis | GBIF | LC | 3 |
| Reptiles | Takydromus_khasiensis | Overall | LC | 5 |
| Reptiles | Trimeresurus_albolabris | Facebook | LC | 25 |
| Reptiles | Trimeresurus_albolabris | GBIF | LC | 1 |
| Reptiles | Trimeresurus_albolabris | Overall | LC | 26 |
| Reptiles | Trimeresurus_erythrurus | Facebook | LC | 7 |
| Reptiles | Trimeresurus_erythrurus | GBIF | LC | 11 |
| Reptiles | Trimeresurus_erythrurus | Overall | LC | 18 |
| Reptiles | Trimeresurus_popeiorum | Facebook | VU | 1 |
| Reptiles | Trimeresurus_popeiorum | Overall | VU | 1 |
| Reptiles | Tropidophorus_assamensis | Facebook | VU | 2 |
| Reptiles | Tropidophorus_assamensis | Overall | VU | 2 |
| Reptiles | Varanus_bengalensis | Facebook | NT | 7 |
| Reptiles | Varanus_bengalensis | GBIF | NT | 13 |
| Reptiles | Varanus_bengalensis | Overall | NT | 20 |
| Reptiles | Varanus_flavescens | Facebook | NT | 5 |
| Reptiles | Varanus_flavescens | GBIF | NT | 5 |
| Reptiles | Varanus_flavescens | Overall | NT | 10 |
| Reptiles | Varanus_salvator | Facebook | VU | 4 |
| Reptiles | Varanus_salvator | GBIF | VU | 10 |
| Reptiles | Varanus_salvator | Overall | VU | 14 |
| Reptiles | Xenochrophis_cerasogaster | Facebook | LC | 18 |
| Reptiles | Xenochrophis_cerasogaster | GBIF | LC | 3 |
| Reptiles | Xenochrophis_cerasogaster | Overall | LC | 21 |
| Reptiles | Xenochrophis_piscator | Facebook | LC | 39 |
| Reptiles | Xenochrophis_piscator | GBIF | LC | 58 |
| Reptiles | Xenochrophis_piscator | Overall | LC | 97 |

**Figure S1**: Density of the geospatial records for Bangladeshi taxa, obtained from GBIF and Facebook.


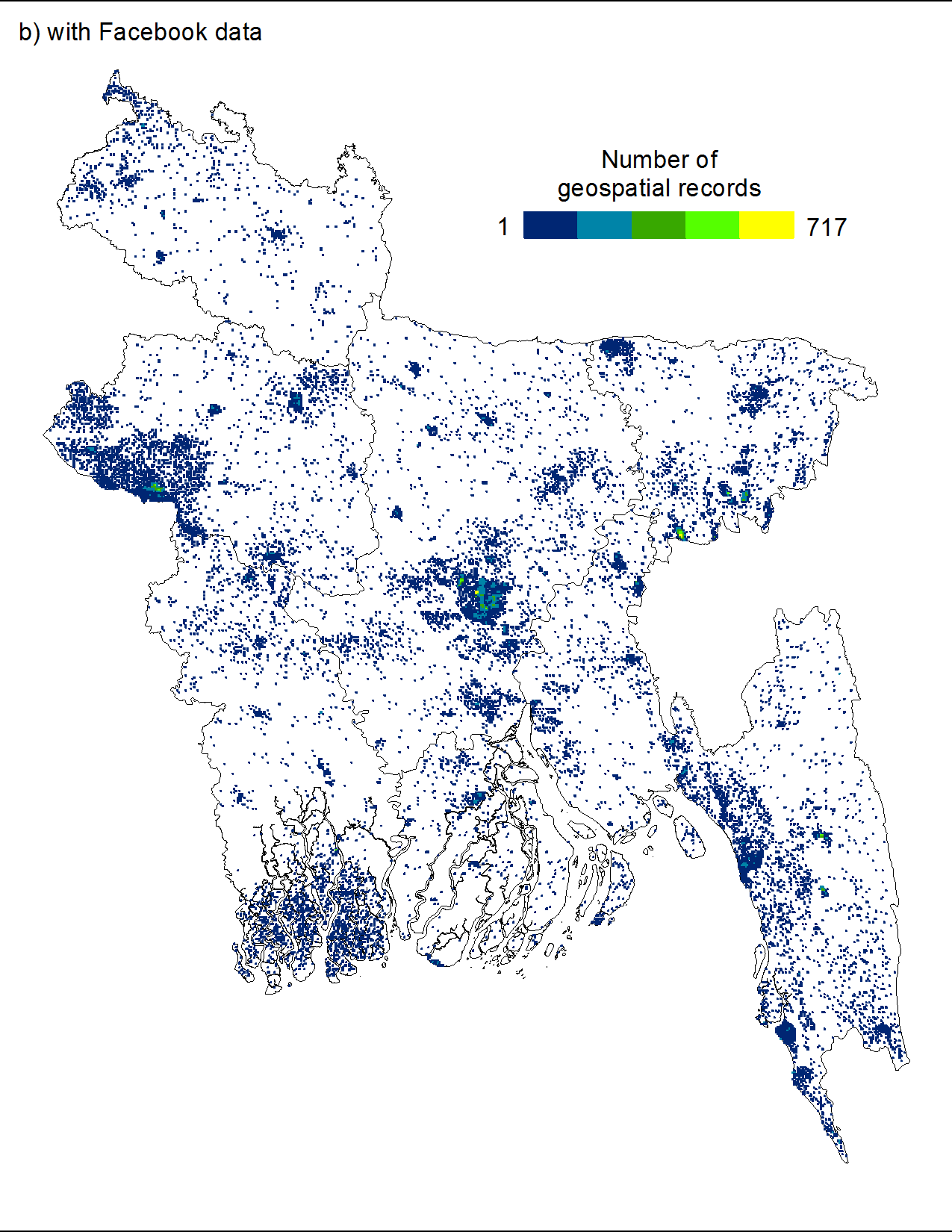

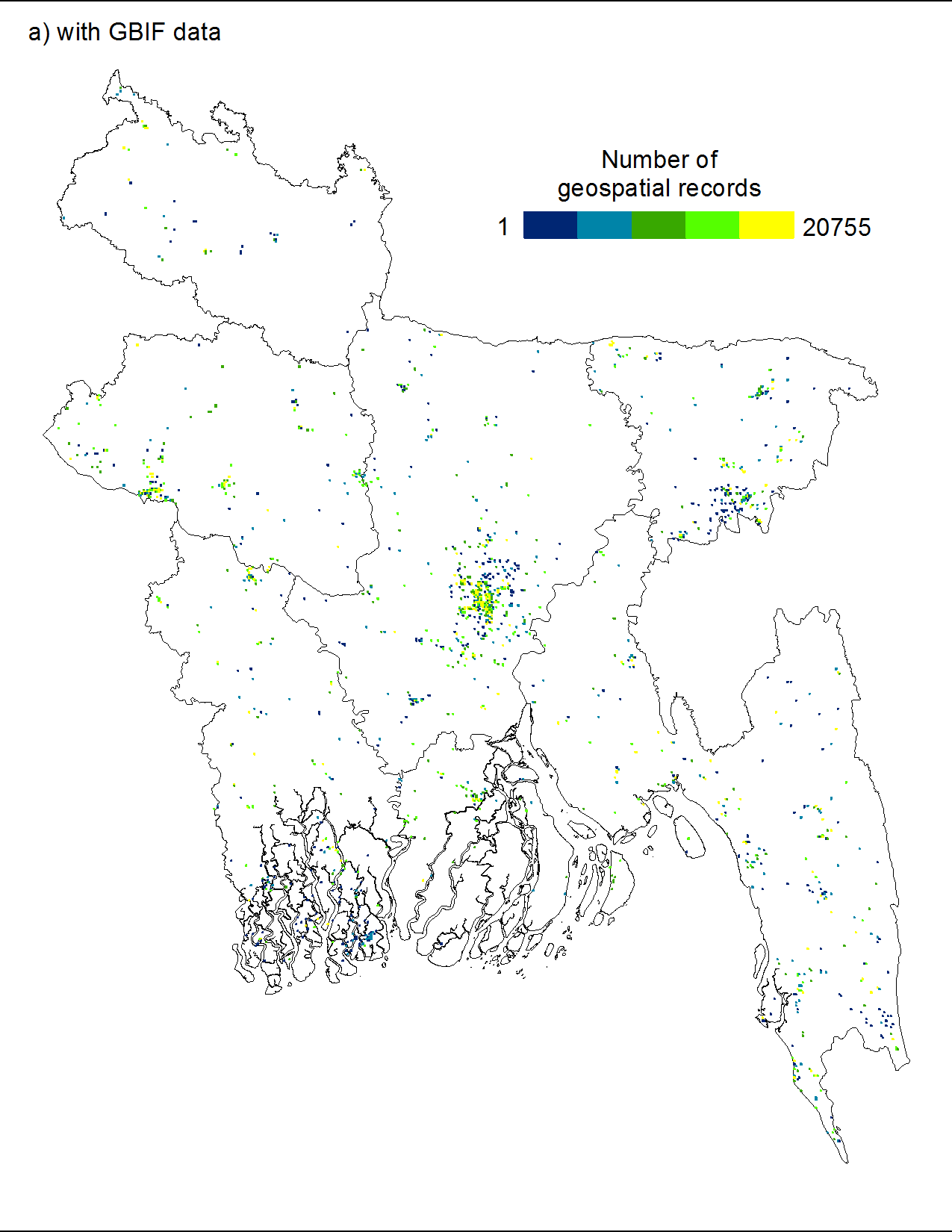

Supplement: biad042_Supplemental_File [file biad042_supplemental_file.docx]
